# Auxin-inducible degron 2 system deciphers functions of CTCF domains in transcriptional regulation

**DOI:** 10.1186/s13059-022-02843-3

**Published:** 2023-01-26

**Authors:** Judith Hyle, Mohamed Nadhir Djekidel, Justin Williams, Shaela Wright, Ying Shao, Beisi Xu, Chunliang Li

**Affiliations:** 1 Department of Tumor Cell Biology, St. Jude Children’s Research Hospital, 262 Danny Thomas Place, Memphis, TN 38105 USA; 2 Center for Applied Bioinformatics, St. Jude Children’s Research Hospital, 262 Danny Thomas Place, Memphis, TN 38105 USA; 3 Department of Computational Biology, St. Jude Children’s Research Hospital, 262 Danny Thomas Place, Memphis, TN 38105 USA

**Keywords:** CTCF, Transcription, Auxin-inducible degron, CRISPR

## Abstract

**Background:**

CTCF is a well-established chromatin architectural protein that also plays various roles in transcriptional regulation. While CTCF biology has been extensively studied, how the domains of CTCF function to regulate transcription remains unknown. Additionally, the original auxin-inducible degron 1 (AID1) system has limitations in investigating the function of CTCF.

**Results:**

We employ an improved auxin-inducible degron technology, AID2, to facilitate the study of acute depletion of CTCF while overcoming the limitations of the previous AID system. As previously observed through the AID1 system and steady-state RNA analysis, the new AID2 system combined with SLAM-seq confirms that CTCF depletion leads to modest nascent and steady-state transcript changes. A CTCF domain sgRNA library screening identifies the zinc finger (ZF) domain as the region within CTCF with the most functional relevance, including ZFs 1 and 10. Removal of ZFs 1 and 10 reveals genomic regions that independently require these ZFs for DNA binding and transcriptional regulation. Notably, loci regulated by either ZF1 or ZF10 exhibit unique CTCF binding motifs specific to each ZF.

**Conclusions:**

By extensively comparing the AID1 and AID2 systems for CTCF degradation in SEM cells, we confirm that AID2 degradation is superior for achieving miniAID-tagged protein degradation without the limitations of the AID1 system. The model we create that combines AID2 depletion of CTCF with exogenous overexpression of CTCF mutants allows us to demonstrate how peripheral ZFs intricately orchestrate transcriptional regulation in a cellular context for the first time.

**Supplementary Information:**

The online version contains supplementary material available at 10.1186/s13059-022-02843-3.

## Background

CTCF is a multi-functional protein that organizes chromatin architecture and plays varied roles in transcriptional regulation by acting as both a transcriptional activator and repressor [1]. CTCF represses transcription through its well-described role as an insulator at topologically associated domain (TAD) boundaries or as an enhancer blocker [2–8]. Transcriptional activation occurs when CTCF acts as a transcription factor at promoters or as a promoter-enhancer looping factor [9–12]. Well established as an integral component of chromatin architectural organization [13–16], CTCF’s loss results in TAD integrity collapse, abolished chromatin looping, and genome-wide changes to chromatin accessibility [12, 17, 18]. Despite these large-scale changes, transcriptional dysregulation following CTCF loss is modest [12, 18, 19].

CTCF principally functions through its interaction with DNA, which occurs via its conserved 11 repeat Cys2His2 (C2H2) zinc-finger (ZF) domain [1, 13, 14, 20]. Biochemistry and structural studies previously showed that ZFs 2–9 exhibited DNA sequence-specific interactions, while ZFs 1 and 10 lacked functional binding [21, 22]. ZFs 3–7 act as the anchor region of the domain that directly interacts with CTCF’s core consensus sequence motif [21–24]. Loss of ZFs in this region caused significant disruptions to DNA binding and chromatin organization [22]. Motifs up and downstream of the core sequence have been predicted to stabilize CTCF binding via interactions with ZFs at the periphery of the ZF domain [22, 25–27]. In addition, Saldana-Meyer et al. partially disrupted ZFs 1 and 10, demonstrating these regions function through interactions with RNA rather than DNA, which stabilized chromatin binding and had some effects on gene expression and chromatin organization [28]. In addition to the ZF domain, an RNA binding region (RBR) located at the C-terminus supports CTCF dimerization and orchestrates RNA-dependent chromatin organization [29, 30]. Although much has been discovered about how the different domains within CTCF work, previous domain studies were conducted in vitro or in vivo by overexpressing domain mutants while endogenous CTCF remained intact. Therefore, it is challenging to evaluate the crosstalk and impact between the ectopic and endogenous forms. To overcome these limitations, we developed a model to easily switch from endogenous CTCF expression to induced exogenous expression of domain mutants.

Compared with loss of function studies designed to disrupt DNA sequences or RNA transcripts, protein degradation studies, including the auxin-inducible degron (AID) system, offer the benefit of directly and reversibly removing the protein of interest without any off-target effects on the genome [31, 32]. However, the AID system does have some limitations, including leaky degradation in the absence of auxin, poor constitutive expression of OsTIR1, and, in particular, the need to use high concentrations of indole-3-acetic acid (auxin) to achieve degradation, which could cause cytotoxicity [33, 34]. To overcome these limitations, Yesbolatova et al. developed a new AID system, AID2, which used a mutated OsTIR1, OsTIR1(F74G), and auxin analog, 5-phenyl-indole-3-acetic acid (5-Ph-IAA), to achieve rapid and efficient protein degradation using hundreds fold less drug [33].

In this study, we demonstrated the AID2 system improved transcriptional studies following acute degradation of CTCF in the B-ALL SEM cell line by facilitating more rapid degradation of CTCF without cytotoxic effects. As was previously shown, despite causing global chromatin architecture changes, acute loss of CTCF had surprisingly little effects on transcription. To identify regions within CTCF connected with its transcriptional response, we employed a CTCF domain sgRNA library screen that identified the ZF domain as CTCF’s most functional domain. Furthermore, by combining the new AID2 system with induced expression of CTCF ZF mutants, we created a model that can switch from endogenous CTCF expression to induced mutant expression to study specific effects of mutant loss. Here, we revealed that ZF1 and ZF10 were required for binding CTCF to mutually exclusive regions within the genome that exhibited characteristic CTCF binding motifs and were correlated with transcriptional regulation of a subset of genes regulated by CTCF.

## Results

### AID2 facilitated rapid degradation of CTCF with reduced cellular toxicity

**Fig. 1:**
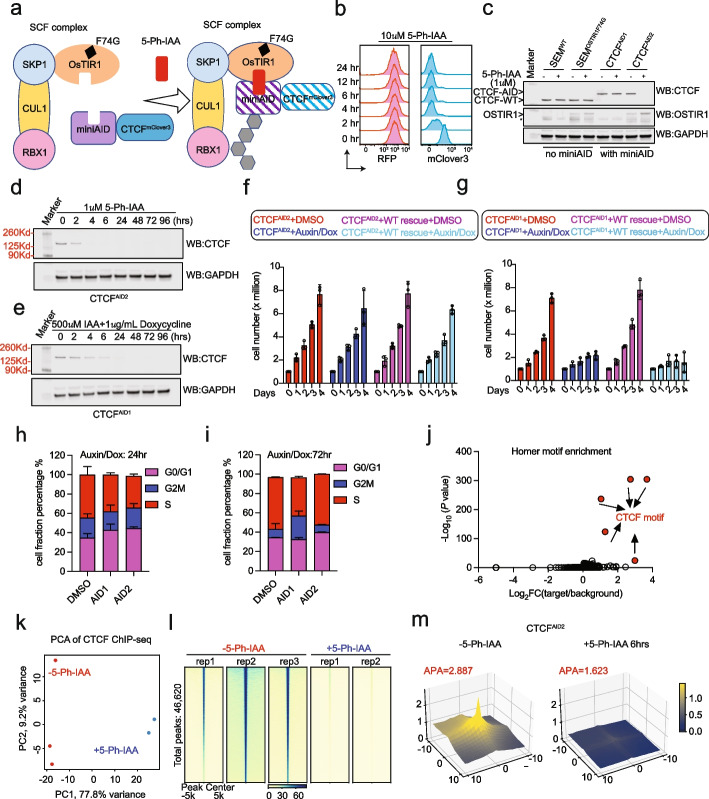
The AID2 system promoted rapid CTCF degradation, reduced cellular toxicity, and genome-wide loss of CTCF binding and chromatin looping. **a** Diagram illustrating how the AID2 system works. The 5-Ph-IAA auxin analog binds to the miniAID tag and recruits the SCF complex by binding to the OsTIR1(F74G) protein. **b** Flow cytometry analysis was performed on CTCF^AID2^ cells following 10 μM 5-Ph-IAA treatment over a time course from 0 to 24 h. RFP fluorescence represents exogenous expression from lentiviral integration of the pCDH-MND-OsTIR1(F74G)-P2A-EGFP^AID2^-EF1a-RFP construct. Endogenous CTCF was expressed in frame with a miniAID tag and mClover3 (CTCF^AIDmClover3^), and exogenous EGFP from the pCDH-MND-OsTIR1(F74G)-P2A-EGFP^AID2^-EF1a-RFP construct was expressed in frame with a miniAID tag (EGFP^AID2^). The fluorescence of both CTCF^AIDmClover3^ and EGFP^AID2^ was detected in the mCLover3 panel. **c** Immunoblot analysis from SEM^WT^, SEM^OsTIR1(F74G)^, CTCF^AID1^, and CTCF^AID2^ cells following 24 h of DMSO or 1 μM 5-Ph-IAA treatment. The lower CTCF band represents untagged endogenous CTCF (CTCF-WT). The higher CTCF band represents CTCF^AIDmClover3^ (CTCF-AID). GAPDH was included as a loading control. **d** Immunoblot analysis of CTCF^AID2^ cells treated with AID2 conditions over a 4-day time course. CTCF antibody was used to detect CTCF^AIDmClover3^. **e** Immunoblot analysis of CTCF^AID2^ cells treated with AID1 conditions over a 4-day time course. CTCF antibody was used to detect CTCF^AIDmClover3^. **f** Growth assay of CTCF^AID2^ cells treated with AID2 conditions over 4 days. Samples were set up in triplicate and cell counts for each replicate were collected daily. Bars represent cell numbers (millions). **g** Growth assay of CTCF^AID2^ cells treated with AID1 conditions over 4 days. Samples were set up in triplicate and cell counts for each replicate were collected daily. Bars represent cell numbers (millions). **h** Cell cycle analysis was performed on samples from the 24-h time points from **f** and **g** and plotted for cell fraction percentage according to cell cycle phase. **i** Cell cycle analysis was performed on samples from the 72-h time points from **f** and **g** and plotted for cell fraction percentage according to cell cycle phase. **j** Homer motif enrichment analysis illustrated the overall distribution of motifs identified in CTCF ChIP-seq of CTCF^AID2^ cells without auxin treatment. Enriched CTCF motifs were shown as red circles. **k** Principal component analysis from CTCF ChIP-seq of CTCF^AID2^ cells with (2 replicates) or without (3 replicates) 10 μM 5-Ph-IAA treatment for 24 h. **l** Genomic heatmap centered at reproducible CTCF peak summits from CTCF^AID2^ cells with (2 replicates) or without (3 replicates) 10 μM 5-Ph-IAA treatment for 24 h. **m** Aggregate peak analysis (APA) plots from CTCF HiChIP of CTCF^AID2^ cells with or without 10 μM 5-Ph-IAA treatment for 6 h (2 replicates each, merged after confirmation of reproducibility). 7220 loops were called in CTCF HiChIP without 5-Ph-IAA. The *x*/*y*-axis was centered at loop anchors spanning from − 10 to + 10 windows (window size 10 kb), and the *z*-axis was normalized aggregated contact frequency

The new CTCF^AID2^ cell line constitutively expresses OsTIR1(F74G)-P2A-EGFP^AID2^ in a previously derived SEM B−ALL cell line that contains the endogenous CTCF^AIDmClover3^ fusion protein and doxycycline-inducible wild-type (WT) OsTIR1 (CTCF^AID1^) [12], allowing for the comparison between AID1 and AID2 in the same cellular context. Upon the addition of 5-Ph-IAA, the 5-Ph-IAA ligand binds specifically to OsTIR1(F74G) to direct the Skp, Cullin, F-box (SCF) complex to AID-tagged proteins for ubiquitination and degradation (Fig. 1a) [33]. CTCF^AID2^ cells were treated with 10 μM 5-Ph-IAA for 24 h and assessed for mClover3 fluorescence (Fig. 1b). Control RFP expression remained constant, demonstrating that OsTIR1(F74G) was constitutively expressed (Fig. 1b). At 2 h post-treatment, mClover3 fluorescence, which represented CTCF^AID2^ and EGFP^AID2^ expression, was markedly reduced after 4 h corresponding to immunoblot analysis that showed CTCF^AID2^ was undetectable after 4 h of treatment and EGFP^AID2^ after 2 h (Additional file 1: Fig. S1a). To examine the sensitivity of CTCF^AID2^ degradation, CTCF^AID2^ cells were treated with a titration of 5-Ph-IAA from 0.001 to 10 μM over a 6-h time course. Immunoblotting analysis showed CTCF^AID2^ was undetectable at concentrations as low as 0.01 μM (Additional file 1: Fig. S1b). Concurrently treating cells with 5-Ph-IAA and the proteasome inhibitor MG132 rescued CTCF auxin-induced degradation (Additional file 1: Fig. S1c).

To examine whether requirements of the auxin degradation system, including miniAID tagging of CTCF and OsTIR1 expression, might affect CTCF expression prior to auxin-induced degradation, we examined CTCF expression levels in various cell lines and AID treatment settings. First, SEM^WT^ and CTCF^AID1^ cells were treated with 500 μM IAA and 1 μg/mL doxycycline, individually and combined for 24 h. As expected, endogenous CTCF expression in SEM^WT^ cells was unaffected by all treatment conditions (Additional file 1: Fig. S1d). Tagged CTCF expression in CTCF^AID1^ cells was comparable to SEM^WT^ levels without treatment and with IAA alone. However, induction of OsTIR1 by doxycycline caused leaky degradation of CTCF, a known limitation of the AID system using WT OsTIR1 [33–35]. Next, we examined CTCF expression levels in the AID2 system by comparing CTCF expression between SEM^WT^, SEM^OsTIR1(F74G)^ [SEM^WT^ cells expressing OsTIR1(F74G)], CTCF^AID1^, and CTCF^AID2^ cells with and without 1 μM 5PhIAA treatment for 24 h (Fig. 1c). CTCF expression was consistent between SEM^WT^, SEM^OsTIR1(F74G)^, and CTCF^AID1^ regardless of OsTIR1(F74G) expression, tagging of CTCF, or 5-Ph-IAA. In addition, no leaky degradation of CTCF was observed in CTCF^AID2^ cells, which have tagged CTCF and OsTIR1(F74G). Degradation was only observed after adding 5-Ph-IAA, supporting the AID2 degradation system overcomes the limitation of leaky degradation observed in the previous AID1 system.

To compare the degradation efficiency between AID1 and AID2, CTCF^AID2^ cells were treated with either 1 μM 5-Ph-IAA (AID2) or 500 μM IAA + 1 μg/mL doxycycline (AID1) for 96 h (Fig. 1d, e). While CTCF^AID2^ showed undetectable levels of CTCF by 4 h post-treatment, CTCF^AID1^ required 24 h and five 100-fold more drug to achieve similar degradation. Both systems showed degradation was stable over time with no CTCF^AID^ recovery observed by 96 h.

Next, a growth assay was performed to compare cell proliferation over 4 days using the AID1 and AID2 systems. CTCF^AID1^ and CTCF^AID2^ cells carrying doxycycline-inducible WT CTCF were included as rescue controls. Cells treated with the AID2 system only showed a very slight decrease in cell number by the end of the assay (Fig. 1f). However, a striking growth retardation was observed following the AID1 treatment regimen that could not be rescued by the induced expression of WT CTCF (Fig. 1g). In addition, cell growth arrest increased in the AID1-treated cells, which paused at the G_2_-M checkpoint and exhibited a shortened S phase (Fig. 1h, i). To further examine whether treatment conditions, CTCF degradation, or a combination of both were responsible for the severe growth restriction observed using the AID1 system, SEM^WT^ cells and CTCF^AID1^ cells were treated with 500 μM IAA and 1 μg/mL doxycycline, individually and combined, over a 4-day growth assay. High auxin treatment alone significantly reduced proliferation in SEM^WT^ cells, which was compounded in CTCF^AID1^ cells following CTCF degradation (Additional file 1: Fig. S1e-f). In contrast, SEM^WT^, SEM^OsTIR1(F74G)^, CTCF^AID1^, and CTCF^AID2^ cells showed no growth defects following 5-Ph-IAA treatment (Additional file 1: Fig. S1g). Taken together, these data support the AID2 system was superior for rapidly degrading CTCF with reduced cellular toxicity.

### CTCF^AID2^ degradation led to a genome-wide loss of CTCF DNA binding and chromatin looping

As was previously observed following AID1 depletion of CTCF [12, 18], CTCF ChIP-seq showed 5-Ph-IAA treatment globally removed CTCF DNA binding. As expected, the Homer motif analysis identified the CTCF consensus binding motif as the top enriched transcription factor motif (Fig. 1j). CTCF binding peaks were assigned to loci that exhibited CTCF motif enrichment and high confidence reproducibility between replicates. Principal component analysis (PCA) confirmed peaks assigned in the CTCF^AID2^ treatment groups were highly correlated with significant variation observed between 5-Ph-IAA-treated and untreated cells (Fig. 1k). When reproducible peaks were combined from both treated and untreated conditions, a total of 46,620 peaks were called (Fig. 1l). Upon 24-h treatment with 10 μM 5-Ph-IAA, 27,262 CTCF-bound peaks were significantly reduced, accompanied by a complete loss of 19,291 peaks after CTCF degradation. An additional 67 peaks were called in 5-Ph-IAA-treated cells but with low confidence (Additional file 1: Fig. S2a).

Although CTCF appears completely degraded following 5-Ph-IAA treatment by immunoblot analysis, a small fraction of CTCF remained, which can be seen at the 27,262 peaks that were retained following 5-Ph-IAA treatment. Persistent CTCF binding following CTCF depletion by either auxin or RNAi has been observed before by us and others [12, 18, 19, 36]. When we further examined the retained peaks, we observed that, prior to treatment, they exhibited a much greater peak intensity than lost peaks (Additional file 1: Fig. S2b). No significant genomic distribution differences were observed when comparing the total population of lost and significantly reduced peaks (Additional file 1: Fig. S2c). However, the genomic distribution between the most significantly reduced or lost CTCF peaks (FDR ≤ 0.05, FC ≥ 2) showed that the significantly reduced CTCF peaks were predominantly located at promoters. In contrast, the lost peaks were found in non-promoter regions (Additional file 1: Fig. S2d). Additionally, while the significantly reduced peaks (FDR ≤ 0.05, FC ≥ 2) were located slightly closer to a TAD boundary, there was no statistical significance when compared to total lost peaks (Kolmogorov–Smirnov test) (Additional file 1: Fig. S2e). As CTCF is a survival essential gene in SEM cells [37], and we did not notice a significant change in cell proliferation following 5-Ph-IAA treatment (Fig. 1f; Additional file 1: Fig. S1g), the persistent binding of CTCF at a subset of CTCF binding sites may help support survival.

In addition, CTCF HiChIP improved the capture of CTCF-dependent loops compared to HiC and showed a global reduction in CTCF anchored chromatin loops following 6 h of 5-Ph-IAA treatment, with a strong correlation observed between replicates (Fig. 1m; Additional file 1: Fig. S3a-b). Untreated cells exhibited 7220 chromatin loops, of which 6852 were lost following CTCF degradation (Additional file 1: Fig. S3b). A small set of loops were either retained or gained; however, the gained, or “new,” loops had low aggregate peak analysis (APA) signals, and the percentage of both anchors overlapping CTCF peaks was much lower (~ 30%) compared to others (> 60%).

To determine how loop anchors colocalized, CTCF HiChIP loop anchors were compared to H3K27ac data for SEM from GEO (GSM1934089). Based on the common nomenclature, the peaks called for H3K27ac that were not associated with a TSS were considered enhancers (E). Anchors overlapping both 2 kb + / − a TSS and an H3K27ac peak were assigned to promoter (P). All anchors not associated with a TSS or H3K27ac were assigned to CTCF. Notably, there was a similar distribution of P-P (*p* = 0.066) and E-E (*p* = 0.045) loops between the retained and lost loops. However, lost loops had significantly fewer P-E loops (*p* = 0.001245, odds ratio = 1.636, Fisher’s exact test) (Additional file 1: Fig. S3c). It is worth noting that although statistically significant, the total loop number is low. Therefore, the biological significance of these comparisons requires extensive investigation in the future.

When loop anchor locations were compared to CTCF ChIP-seq, 72.1% of lost loop anchors overlapped CTCF peaks, while 72.4% of retained loop anchors overlapped CTCF peaks, demonstrating the loop anchors colocalized with CTCF binding sites. When we correlated CTCF binding status to loop anchor regions lost after treatment, we observed that 65.4% of lost loop anchors overlapped CTCF retained peaks, while only 11.3% overlapped CTCF lost peaks. Although the small percentage of lost loops that overlapped lost CTCF peaks may initially seem unexpected, as we previously reported, retained CTCF binding after 5-Ph-IAA treatment was observed but with significantly reduced peak intensity compared to untreated samples (Fig. 1l; Additional file 1: Fig. S2a). To access the correlation of binding intensity to loop anchor loss, we compiled the log_2_ (fold change) for CTCF peaks and separated them into two groups depending on whether they overlapped lost loop anchors or overlapped retained loop anchors. The results indicated that all CTCF binding was decreased, while the decreasing magnitude of binding at CTCF peaks overlapping the anchors for lost loops was significantly lower when compared to the peaks overlapping retained loops (Additional file 1: Fig. S3d). Therefore, complete abrogation of CTCF binding was not necessary to disrupt CTCF-mediated chromatin looping.

### AID2 improved gene expression analysis by SLAM-seq following CTCF acute depletion

**Fig. 2:**
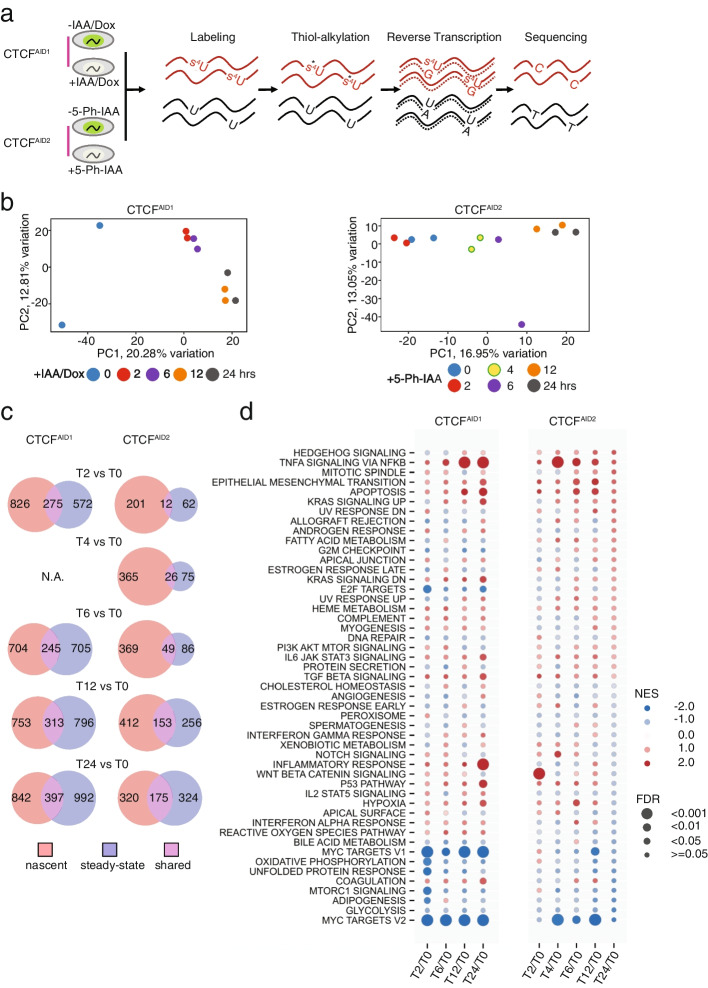
AID2 improved gene expression analysis by SLAM-seq. **a** Schematic diagram illustrating the SLAM-seq experiment. **b** Unsupervised data dimensionality reduction by PCA of the top 3000 most variably expressed nascent transcripts (summarized at the gene level) as determined by median absolute deviation. SLAM-seq quantified nascent gene expression from CTCF^AID1^ and CTCF.^AID2^ cells that underwent AID1 or AID2 auxin treatment conditions over a 24-h time course. Time 0 samples were untreated. RNA was collected following 0, 2, 6, 12, and 24 h of AID1 treatment and after 0, 2, 4, 6, 12, and 24 h of AID2 treatment. Samples for each time point were performed in duplicate. **c** Venn diagrams highlight the comparison of T > C converted reads (termed nascent and colored red) and all or total reads (termed steady-state and colored blue) of DE genes identified in each corresponding AID1 or AID2 auxin depletion SLAM-seq time-course experiment. DE genes determined in both nascent and steady states are colored purple. Genes passing thresholds of *p* value ≤ 0.05 and |log_2_(FC)|≥ 1 were determined to be differentially expressed. T0 = no AID treatment; T2 = 2-h AID treatment; T4 = 4-h AID treatment; T6 = 6-h AID treatment; T12 = 12-h AID treatment; T24 = 24-h AID treatment. **d** Gene set enrichment analysis of nascent transcripts summarized at the gene level corresponding to AID1 or AID2 auxin depletion SLAM-seq time-course experiments. Up-regulation and down-regulation by normalized enrichment score (NES) of hallmark gene sets compared to T0 are denoted in red and blue, respectively. Circle size indicates the false discovery rate (FDR)

Using the AID1 system, we previously showed that CTCF depletion for 48 h minimally impacted genome-wide transcription despite the global loss of chromatin architectural integrity and accessibility [12, 17]. To address whether modest transcriptional changes previously observed after CTCF loss were due to the examination of steady-state RNA levels versus nascent transcriptional changes, we used thiol (SH)-linked alkylation for the metabolic sequencing of RNA (SLAM-seq) to compare nascent RNA transcription changes using the AID1 and AID2 systems. SLAM-Seq allows for the quantification of newly transcribed mRNAs by incorporating a 4-thiouridine (4sU) into the RNA that will go on to be identified as a thymine-to-cytosine (T > C) conversion in 3′-end mRNA-sequencing (Fig. 2a) [38]. CTCF^AID1^ and CTCF^AID2^ cells were treated with either 500 μM IAA, 1 μg/mL doxycycline (AID1), or 10 μM 5-Ph-IAA (AID2) for a total of 24 h with collection time points throughout. T > C conversion was successful and showed no strand bias for both AID1 and AID2 treatment groups (Additional file 1: Fig. S4a). PCA of SLAM-seq data revealed that PC1 generally correlated with the time of treatment in PCA plots (Fig. 2b). However, for AID1, this correlation was predominantly treatment dependent as indicated by the clustering of untreated samples (on the left) versus treated samples (clustered together on the right), indicating the nascent gene expression contributing to PC1 were in response to + IAA/Dox, not decreasing levels of CTCF (Fig. 2b). However, the treatment samples did not cluster together in AID2, indicating those genes contributing to PC1 were more correlated with treatment time and subsequent CTCF degradation instead of a binary response to + 5-Ph-IAA. As expected, the AID2 system resulted in a time-dependent accumulation of differential nascent RNA transcript populations in response to early CTCF loss starting at 2 h post-5-Ph-IAA treatment (T2) and with a peak of nascent transcript differential expression (DE) observed at 12 h post-treatment (T12) (*p* ≤ 0.05, |log_2_FC|≥ 1) (Fig. 2c). Steady-state (the superset of reads including T > C converted and unconverted reads) DE appeared at T2 and increased at T12 and T24. In contrast, AID1 cells accumulated 1101 nascent transcript and 847 steady-state RNA transcript changes following 2 h of IAA treatment (T2) (Fig. 2c), which did not correspond to a significant reduction in CTCF protein (Fig. 1e), suggesting AID1 treatment conditions could affect transcriptional response.

To determine to what extent AID1 treatment conditions contributed to transcriptional dysregulation, differential gene expression was analyzed between SEM^WT^ and CTCF^AID1^ cells following AID1 treatment and CTCF^AID2^ cells following AID2 treatment. Total RNA was collected after 24 h of treatment and total-stranded RNA-seq was performed. When comparing SEM^WT^ and CTCF^AID1^ cells following AID1 treatment, 196 genes (82 up, 114 down) were differentially expressed, including genes associated with amino acid metabolism, apoptosis, and ER stress (Additional file 1: Fig. S5a-b). Pathways enriched for DE following treatment included the krige amino acid deprivation, hallmark TNFA signaling, and hallmark unfolded protein response (Additional file 1: Fig. S5c). Spearman’s correlation between AID1-treated SEM^WT^ and CTCF^AID1^ cells showed some correlation in DE genes, suggesting dysregulation of these genes resulted from auxin treatment alone (Additional file 1: Fig. S5d). When SEM^WT^ cells treated with AID1 conditions were compared to CTCF^AID2^ cells treated with AID2 conditions, no significant correlation was observed, highlighting that CTCF^AID2^ cells treated with low auxin did not develop transcriptional dysregulation attributed to high auxin toxicity (Additional file 1: Fig. S5e). CTCF^AID1^ and CTCF^AID2^ cells treated with either high auxin and doxycycline (AID1) or 5-Ph-IAA (AID2) demonstrated a correlation of DE genes that corresponded to those genes dysregulated by CTCF loss (Additional file 1: Fig. S5f). Taken together, these data suggest the genes DE following AID1 treatment could be attributed to genes dysregulated by high auxin toxicity, CTCF degradation, or a combined effect of both conditions. Therefore, the AID2 system, which does not have auxin toxicity, was preferred for studying acute CTCF degradation effects on transcription.

Previously, we showed acute depletion of CTCF in SEM cells resulted in dysregulation of *MYC* transcription pathways [12]. Gene set enrichment analysis (GSEA) of nascent transcript populations showed early enrichment of decreased DE in hallmark MYC targets V1 and V2 following AID1 treatment, confirming observations from the previous study (Fig. 2d). AID2 also showed a decrease in DE enrichment in the MYC targets V1 and V2 pathways, but the enrichment peaked after 4 h of treatment for MYC targets V2 and 12 h for MYC targets V1, corresponding with undetectable levels of CTCF (Fig. 1d). These data confirmed that *MYC* was a direct target of CTCF. However, the early response observed for MYC targets using the AID1 system could not be supported by CTCF degradation alone since CTCF was not fully depleted by immunoblot analysis until after 24 h of treatment (Fig. 1e), nor could it be attributed to auxin toxicity since MYC targets were not affected by AID1 treatment in SEM^WT^ cells, suggesting undetermined effects of combined high concentration auxin treatment and CTCF depletion could destabilize MYC targets.

AID2 SLAM-seq revealed additional pathways enriched for increased DE of nascent transcripts, including the nuclear factor (NF)-Ka signaling via NF-Kb pathway and WNT beta-catenin signaling pathway suggesting targets within these pathways were directly regulated by CTCF, possibly by its insulation properties (Fig. 2d). Overall, global nascent transcription changes following CTCF loss with the AID2 system were limited, which supported previous observations that CTCF loss minimally impacted global steady-state RNA expression [12, 18, 19].

### CTCF domain sgRNA library screen identified functional domains of CTCF

**Fig. 3:**
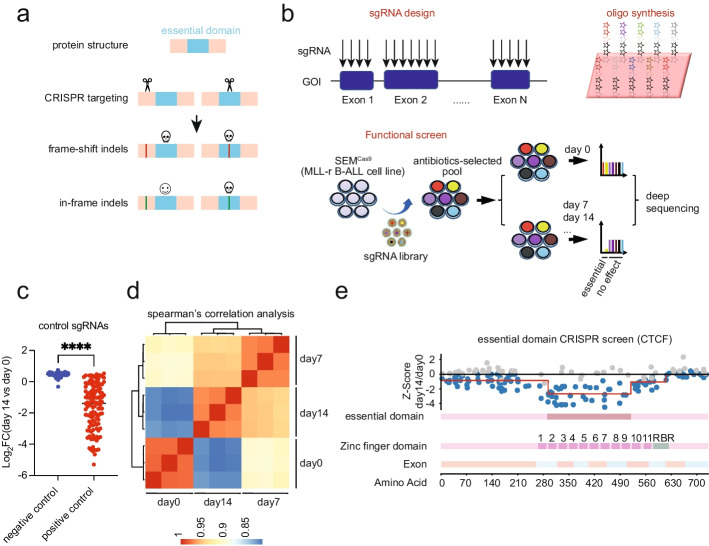
CTCF domain sgRNA library screen. **a** Schematic diagram illustrating CRISPR sgRNA targeting of essential domains. Skulls represent the loss of function caused by frameshift indels either before or within essential domains and in-frame indels within essential domains. The smiley face represents retained function with in-frame indels that do not disrupt essential domains. **b** Diagram illustrating CRISPR sgRNA domain library design and sgRNA drop-out library screen. **c** Log_2_ fold change of positive and negative control sgRNA representation at day 14 vs. day 0. “****” represents FDR ≤ 0.05. **d** Heat map illustrating the Spearman correlation coefficient of a pairwise comparison of sgRNA distribution between sgRNA cell populations collected at day 0, day 7, and day 14. The Spearman correlation coefficient was calculated on normalized sgRNA counts from the MAGeCK analysis. **e** The *z*-score plot of the CTCF domain sgRNAs with their location across the CTCF exons. Gray dots represent filtered sgRNAs after weak signals were removed and outlier adjustments that did not pass a significance test (FDR ≤ 0.05). Exons were colored differently to indicate the boundary between exons

Despite showing a limited impact on global transcription following CTCF loss, we hypothesized certain domains of CTCF, in addition to the well-characterized ZF core binding domain, could sensitize CTCF’s transcriptional response. To identify regions within CTCF that could be targeted to study its transcriptional impact and chromatin binding without fully disrupting the most survival essential domains, we developed a CRISPR (clustered regularly interspaced short palindromic repeats) functional domain single guide RNA (sgRNA) library screen for CTCF. Domain libraries are designed by tiling sgRNAs across a gene’s coding sequence [39]. Nucleic acid disruptions caused by sgRNAs that shift the reading frame in essential genes would drop out of survival screens when targeted before or within essential domains. In contrast, sgRNAs that cause in-frame mutations only drop out when targeting essential domains (Fig. 3a). In the CTCF domain CRISPR sgRNA library, 512 sgRNAs were designed spanning the coding exons of CTCF, 120 sgRNAs were included as positive controls, and an additional 100 non-targeting sgRNAs were included as negative controls. The CTCF sgRNA domain library was cloned into a lentiviral vector with puromycin resistance and CFP fluorescence followed by infection at a low M.O.I. (less than 0.3) into a SEM B-ALL cell line stably expressing Cas9. Following puromycin selection, cells were collected at days 0, 7, and 14 and sequenced for sgRNA distribution (Fig. 3b). The differentially represented sgRNAs were calculated by MAGeCK analysis. As expected, positive control sgRNAs dropped out of the screen while negative control sgRNAs were stably represented on day 14 (Fig. 3c). The Spearman’s correlation coefficient was ≥ 0.9 between the different groups, confirming time-dependent variations in sgRNA distribution (Fig. 3d). Survival dependency was only observed in the ZF domain, with the most significant reduction of sgRNAs seen in ZFs 2–9 (FDR ≤ 0.05), which corresponded to the region of the domain previously shown to control DNA sequence-specific interactions [21] (Fig. 3e). In addition, ZFs 1 and 10 were also enriched for sgRNA loss suggesting these peripheral ZFs were functionally relevant.

### ZF1 and ZF10 mutants exhibited disrupted chromatin binding

**Fig. 4:**
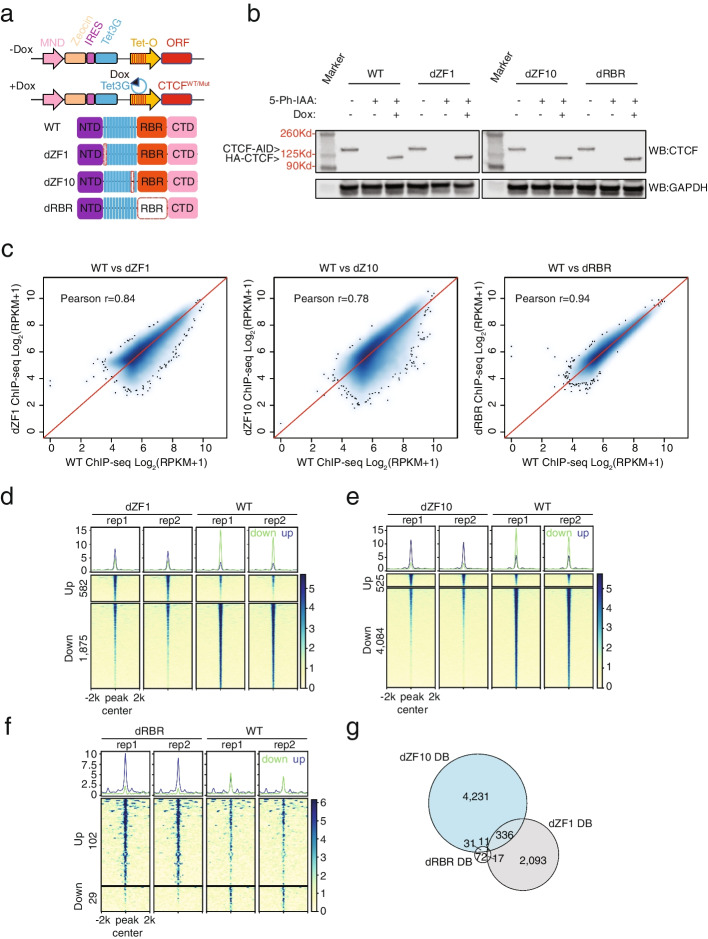
Disrupted chromatin binding of ZF1 and ZF10 mutants. **a** Design of the inducible CTCF HA-tagged constructs. **b** Immunoblot analysis of endogenous (CTCF^AID2^) and induced exogenous (HA-CTCF) expression of CTCF using an antibody for CTCF. CTCF^AID2^ expression can be seen in all untreated samples (− , −). After 6 h of 10 μM 5-Ph-IAA treatment, CTCF^AID2^ expression is depleted (+ , −). Exogenous HA-CTCF expression comparable to endogenous CTCF was only observed following 18 h of 1 μg/mL doxycycline (+ , +). **c** Pearson correlation scatterplot comparing the log_2_ mean RPM enrichment of mutant HA CTCF ChIP-seq peaks to the log_2_ mean RPM enrichment of WT CTCF ChIP-seq peaks. **d** Heatmap of normalized CTCF signal centered at differential CTCF peaks from 2 replicates of HA ChIP-seq of CTCF^AID2/WT^ and CTCF^AID2/dZF1^ cells following 10 μM 5-Ph-IAA treatment for 24 h and 18 h of 1 μg/mL doxycycline. Differential ChIP-seq peak analysis respectively identified 582 and 1875 significantly enriched (up) or depleted (down) peaks (criteria: |log_2_(FC)|≥ 1 and FDR ≤ 0.05, using Limma-Voom analysis) in CTCF^AID2/dZF1^ samples. **e** Heatmap of normalized CTCF signal centered at differential CTCF peaks from 2 replicates of HA ChIP-seq of CTCF^AID2/WT^ and CTCF^AID2/dZF10^ cells following 10 μM 5-Ph-IAA treatment for 24 h and 18 h of 1 μg/mL doxycycline. Differential ChIP-seq peaks analysis respectively identified 525 and 4084 significantly increased (up) or depleted (down) peaks (cutoff: |log_2_(FC)|≥ 1 and FDR ≤ 0.05, using Limma-Voom analysis) in CTCF^AID2/dZF10^ HA samples. **f** Heatmap of normalized CTCF signal centered at differential CTCF peaks from 2 replicates of HA ChIP-seq of CTCF^AID2/WT^ and CTCF^AID2/dRBR^ cells following 10 μM 5-Ph-IAA treatment for 24 h and 18 h of 1 μg/mL doxycycline. Differential ChIP-seq peaks analysis respectively identified 102 and 29 significantly increased (up) or depleted (down) peaks (cutoff: |log_2_(FC)|≥ 1 and FDR ≤ 0.05, using Limma-Voom analysis) in CTCF^AID2/dRBR^ HA samples. **g** Venn diagram illustrating the differential binding profiles of CTCF^AID2/dZF1^, CTCF^AID2/dRBR^, and CTCF^AID2/dZF10^ had little overlap

Since disruption of the core ZF domain globally perturbs CTCF/DNA interactions [22], we decided to examine how peripheral ZFs 1 and 10, which have not previously been shown to exhibit essential properties, contribute to CTCF function. We designed a model that allowed for cells to switch from endogenous CTCF expression to induced exogenous CTCF expression by combining the CTCF^AID2^ cell line with induced overexpression of HA-tagged CTCF-WT and CTCF mutants for ZF1 (dZF1), ZF10 (dZF10), and RBR (dRBR), which was included as a negative control since this region was minimally enriched in the domain screen and does not bind DNA (Fig. 4a). Immunoblot analysis confirmed complete degradation of endogenous CTCF^AID2^ and induced CTCF-HA expression comparable to endogenous for all constructs after induction by doxycycline (Fig. 4b).

The DNA binding affinity of CTCF^AID2/dZF1^, CTCF^AID2/dZF10^, and CTCF^AID2/dRBR^ was assessed by performing ChIP-seq using HA-conjugated beads to pull down the induced HA-tagged CTCFs in CTCF^AID2/WT^, CTCF^AID2/dZF1^, CTCF^AID2/dZF10^, and CTCF^AID2/dRBR^ cell lines depleted for endogenous CTCF. Overall, the global binding intensity among the mutants was similar to CTCF^AID2/WT^ (Additional file 1: Fig. S6a). Pearson’s correlation showed that CTCF^AID2/dZF10^ had the most significant amount of variance in DNA binding when compared to CTCF^AID2/WT^ (Fig. 4c). Heatmaps generated by deepTools [40] also showed that CTCF^AID2/dZF10^ exhibited the most differential binding (DB) among the groups when compared to CTCF^AID2/WT^ (4084 lost/525 gained) (Fig. 4d). CTCF^AID2/dZF1^ also showed DB compared to CTCF^AID2/WT^ (1875 lost/582 gained) (Fig. 4e), but less than was observed for CTCF^AID2/dZF10^. As expected, CTCF^AID2/dRBR^ showed minimal variance in binding when compared to CTCF^AID2/WT^ (Pearson *r* = 0.94) (Fig. 4c), and DB of peaks was not significant (29 lost/102 gained) (Fig. 4f). Genomic distribution of the CTCF binding peaks lost and gained in CTCF^AID2/dZF10^ and CTCF^AID2/dZF1^ was similar to CTCF^AID2/WT^ (Additional file 1: Fig. S6b–h), with a slight increase in promoter localization among gained peaks that were previously observed following acute depletion of CTCF [12]. Notably, the DB profiles between CTCF^AID2/dZF1^ and CTCF^AID2/dZF10^ were distinct with little overlap suggesting these ZFs were independently required for CTCF binding at select genomic locations (Fig. 4g).

### Correlation observed between differential gene expression and disrupted CTCF binding of ZF mutants

**Fig. 5:**
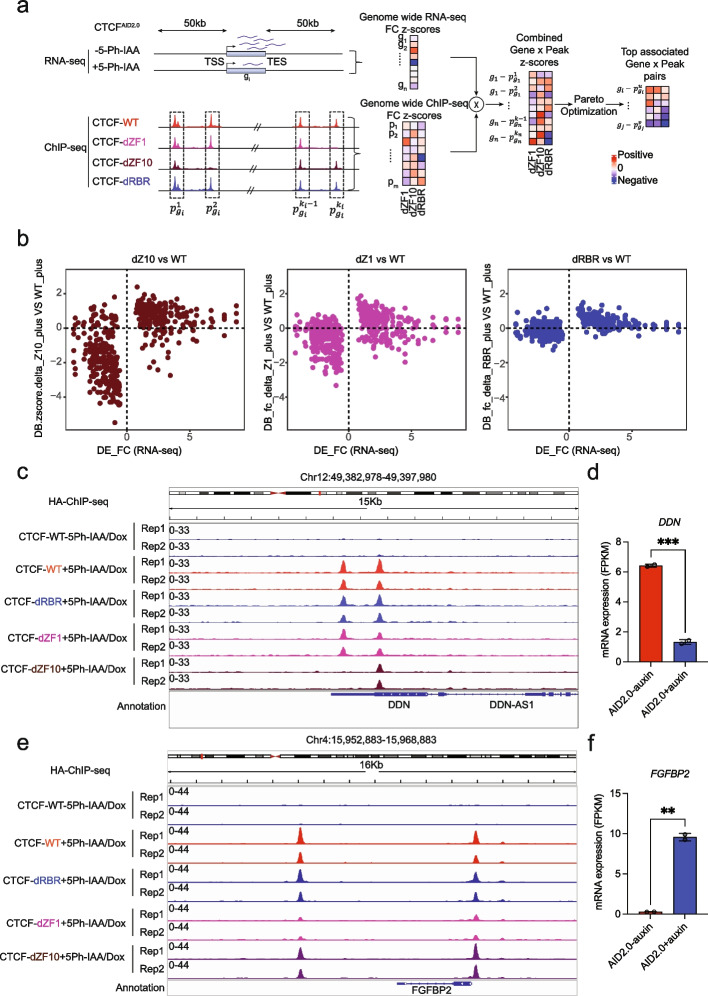
Correlation between differential gene expression and disrupted CTCF binding of ZF mutants. **a** Schematic diagram of the integrative analysis of RNA-seq and differential binding of HA-CTCF-ZF mutants ChIP-seq. For each gene, initially, we considered all the peaks within [TSS − 50 kb, TES + 50 kb]. The RNA-seq and ChIP-seq fold changes were converted to *z*-score, then multiplied together for each gene-peak pair to get a combined score. Pareto optimization was performed to determine the most correlated peak-gene pairs. **b** Scatterplot representing the correlated distribution of DE genes and differentially bound peaks identified using Pareto optimization for ZF1, ZF10, and RBR mutants compared to CTCF WT. The *x*-axis shows the expression fold change of the differentially expressed genes. The *y*-axis represents the binding fold change of the gene-associated peaks. **c** HA ChIP-seq tracks from CTCF WT and mutants at the *DDN* locus showing disrupted CTCF binding in CTCF^AID2/dZF10^ cells. **d** Differential expression of the *DDN* gene by RNA-seq (FKPM) in response to CTCF depletion. ***p ≤ 0.001 calculated by unpaired *t*-test. **e** HA ChIP-seq tracks from CTCF WT and mutants at the *FGFBP2* locus showing disrupted CTCF binding in CTCF^AID2/dZF1^ cells. **f** Differential expression of the *FGFBP2* gene by RNA-seq (FKPM) in response to CTCF depletion. **p ≤ 0.01 calculated by unpaired *t*-test

Since each CTCF ZF mutant exhibited unique dependencies for DNA binding, we hypothesized that loci with disrupted CTCF interactions might correlate with differential gene expression. CTCF binding can regulate transcription either proximally to the transcription start site (TSS) or distally through enhancer regions within the TAD but distinct from TAD boundaries [14, 41–45]. Therefore, the 50-kb region upstream of the TSS through the 50-kb region downstream of the transcription end site (TES) was considered when assigning CTCF binding to DE genes. Steady-state RNA-seq was performed on CTCF^AID2^ cells following treatment with 10 μM 5-Ph-IAA. A total of 753 DE genes were identified with a stringent cutoff (log_2_fold change ≥ 1, adjust *P* ≤ 0.05, CPM ≥ 1) between treated and untreated groups and correlated with genome-wide HA ChIP-seq from above to create a combined Gene/Peak profile. Pareto optimization identified the top associated Gene/Peak pairs (Fig. 5a). The DB profile of CTCF^AID2/dZF10^ exhibited the most correlation with DE (Fig. 5b; Additional file 1: Fig. S7a, d), with a strong correlation between decreased DE and DB. In total, 167 genes differentially expressed after CTCF loss exhibited DB in CTCF^AID2/dZF10^ cells. CTCF^AID2/dZF1^ also showed DE correlated with DB, with 84 genes DE in regions with DB following ZF1 loss (Fig. 5b; Additional file 1: Fig. S7b, d). As expected, the correlation of DE and DB was not seen in the CTCF^AID2/dRBR^ mutant (Fig. 5b; Additional file 1: Fig. S7c, d).

A comparison of the loci correlated with DE and DB from CTCF^AID2/dZF1^ and CTCF^AID2/dZF10^ showed these ZFs regulated mutually exclusive gene sets (Additional file 1: Fig. S7e). As an example, the ChIP-seq track for *DDN* showed two unaltered CTCF binding peaks for CTCF in CTCF^AID2/WT^, CTCF^AID2/dZF1^, and CTCF^AID2/dRBR^. However, the CTCF^AID2/dZF10^ mutant showed a loss of binding at one of the CTCF peaks, which was associated with decreased gene expression (Fig. 5c, d). Similarly, the ChIP-seq track for *FGFBP2* showed reduced CTCF binding was limited to CTCF^AID2/dZF1^, and gene expression increased upon reduced binding (Fig. 5e, f). These examples highlight the unique specificity ZF1 and ZF10 play in regulating the transcription of a subset of genes controlled by CTCF.

### Loci regulated by ZF1 or ZF10 were enriched for a CTCF binding motif associated with its respective binding

**Fig. 6:**
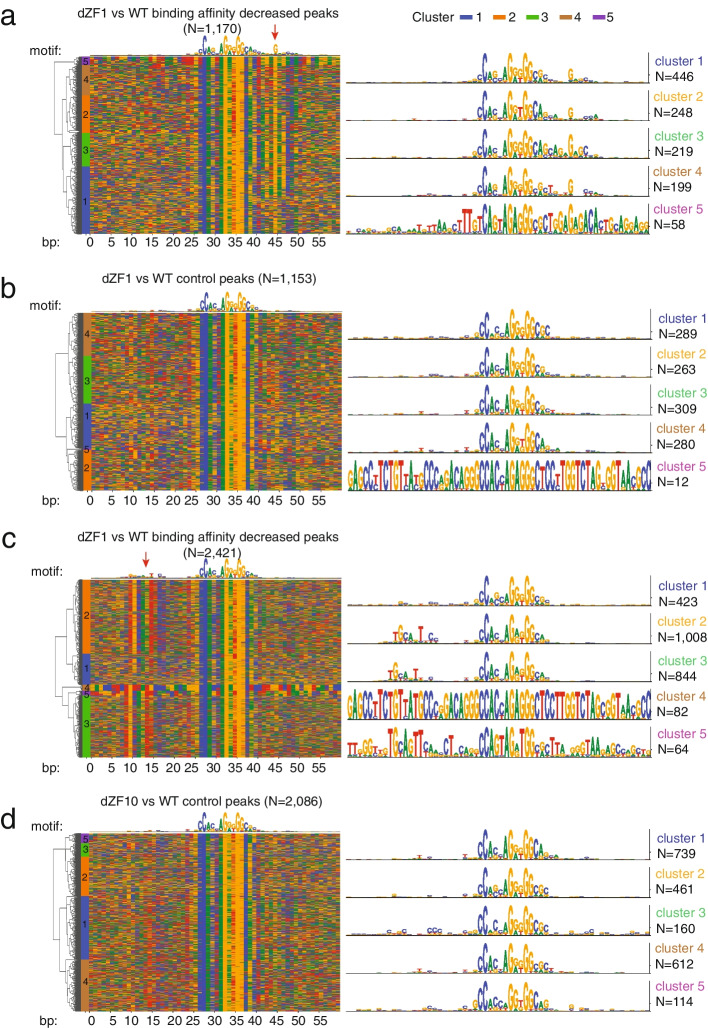
ZF1 and ZF10 differential peaks enriched for specific CTCF binding motifs. **a** Heatmap of motif analysis of CTCF^AID2/dZF1^ decreased peaks identifying conserved DNA signatures including a G (red arrow) positioned 4 bp proximal from the 20-bp consensus sequence motif [46]. **b** Heatmap of motif analysis of CTCF^AID2/dZF1^ control peaks showing no enrichment of downstream DNA signatures. **c** Heatmap of motif analysis of CTCF^AID2/dZF10^ decreased peaks identifying conserved DNA signatures (red arrow) positioned upstream from the 20-bp consensus sequence motif. **d** Heatmap of motif analysis of CTCF^AID2/dZF10^ control peaks showing no enrichment of upstream DNA signatures. For all, green, blue, yellow, and red indicated A, C, G, and T bases, respectively. The right panel shows the motif consensus logo in information bits for five clusters assigned by unsupervised hierarchical clustering using hamming distance

We hypothesized that genes whose regulation by CTCF was dependent on ZF1 or ZF10 for chromatin binding might require additional DNA signatures unique to each ZF, in addition to the core CTCF consensus sequence motif, for CTCF binding. Motif enrichment analysis for ZF1 and ZF10 mutants was performed on the loci identified with DB from the previous analysis. Loci with decreased CTCF binding in CTCF^AID2/dZF1^ cells were enriched to include the 20 base−pair CTCF consensus sequence motif and an additional DNA sequence overlapping and 3′ of the consensus sequence. The motif clustering automatically identified 5 clusters with a slight difference at CTCF consensus core motifs. Strikingly, all 5 clusters were enriched for a dominant G nucleotide positioned at position + 4 proximal to the core motif. In total, the G signature represented more than 82% of decreased CTCF-bound sites (Fig. 6a) [46]. The G signature was not enriched in control peaks, but only in CTCF^AID2/dZF1^ with decreased binding, highlighting it was specifically enriched in loci dependent on ZF1 for binding (Fig. 6b). While the interaction between ZF1 and a motif downstream the consensus sequence motif has been speculated [22], this is the first direct in vivo evidence that ZF1 interacts with a specific DNA signature.

Like observations for ZF1, loci that exhibited decreased CTCF binding upon ZF10 loss were enriched for a sequence 5′ of the core consensus sequence motif that shared similarity to the previously identified and predicted upstream (U) motif [22, 25, 26, 47, 48]. When CTCF^AID2/dZF10^ DB peaks were compared to CTCF^AID2/WT^, the upstream sequence motif was observed in 79.1% of decreased CTCF-bound sites (Fig. 6c). The consensus sequences fell into 5 clusters with the majority in clusters 2 and 3. As was seen for ZF1, there was no enrichment of the upstream motif in control peaks that do not change when comparing CTCF^AID2/dZF10^ against CTCF^AID2/WT^ (Fig. 6d).

Moreover, we also identified a few interesting clusters that demonstrated conserved sequence signature in CTCF^AID2/dZF1^ decreased site cluster 5 (Fig. 6a) and CTCF^AID2/dZF10^ decreased site clusters 4 and 5 (Fig. 6c). The consensus region was too long to be associated with transcription factor motifs. Instead, almost all regions from these clusters could be assigned to a transposable element. Out of 58 sites for CTCF^AID2/dZF1^ decreased site cluster 5, 40 were LTR41 and 18 were LTR41B. All 82 sites for CTCF^AID2/dZF10^ decreased site cluster 4 were LTR13. And 63 out of 64 sites for CTCF^AID2/dZF10^ decreased site cluster 5 could be assigned to LINE L1. It has been reported that LTR13/LTR41/LINE elements were associated with CTCF [49]. Our study confirmed this observation and further suggested specifical ZFs can be related to the individual transposable element type. The functional correlation between these motifs and CTCF protein remains an interesting topic for further investigation.

## Discussion

In this study, we demonstrated the new AID2 degradation system is a powerful and superior tool to degrade endogenous CTCF quickly and efficiently with hundreds of folds less auxin, overcoming combined off-target effects of high auxin concentrations and CTCF depletion. As was previously observed by us and others using the AID1 system [12, 18, 19], CTCF degradation using the AID2 system led to a genome-wide loss of CTCF chromatin binding and looping with a modest effect on genome-wide transcription regulation. A main benefit observed using the new AID2 system was reduced cellular toxicity following CTCF depletion. In addition, SLAM-seq analysis revealed degradation of CTCF using the AID1 system caused a significant early shift in nascent and steady-state gene expression that would not be anticipated due to retained CTCF expression at early time points, which was not observed using AID2, further supporting the AID2 degradation system is superior for transcriptional studies.

Although the mechanism for CTCF interacting with a core consensus DNA sequence via its ZF domain, specifically ZFs 3–7, has been well characterized, the functional significance of the peripheral regions of the ZF domain is less clear. Rhee and Pugh used ChIP-exo to identify a four-part module for CTCF binding, including module 1, which resides upstream of the CTCF binding core motif; modules 2 and 3, which cover the core motif; and module 4, which resides downstream of the core motif [47]. More than half of CTCF binding events required only modules 2 and 3, with the remaining binding events utilizing a combination of 3 or four modules. The upstream motif has also been confirmed by others [25, 26]. Nakahashi et al. developed a model to study CTCF ZF interactions with DNA by mutating histidine residues that coordinate zinc binding [22]. Their study supported that ZFs 4–7 were critical for binding to the core motif and suggested that the peripheral ZFs stabilized CTCF occupancy with a stronger association with binding attributed to ZFs closer to the core motif. Further studies of the crystal structure of CTCF confirmed ZFs 3–7 interact with the core motif and supported ZFs 2–9 make DNA-specific contacts [21], and ZFs 9–11 were shown to interact with module 1, which is observed in 15% of CTCF binding sites [48]. However, these studies’ limitations rely on ectopically expressed CTCF mutants in the presence of an endogenous CTCF background or biochemistry characterization in vitro, which is not ideal for revealing cellular regulation in physiological conditions.

Soochit et al. have generated a similar swapping system using neomycin-resistant lentiviral constructs containing green fluorescent protein (GFP)-tagged wild-type Ctcf or mutants with deletions of individual ZFs together with an IRES-driven Cre in mouse embryonic stem cells (ESCs). These ESC lines carrying a loxP-flanked Ctcf allele were infected with these lentiviruses to delete endogenous Ctcf protein and were rescued by constitutively expressed Ctcf mutants at various expression levels. They found that mouse ESCs expressing Ctcf mutants lacking ZF1 (del1) and ZF10 (del10) are viable with reduced proliferation rates. They also identified the identical flanking sequences associated with del1 and del 10, as described in our study [50]. Of note, while our manuscript was under peer review, a similar study that utilized the ZF1 mutant knockin MCF7 cell line identified the same conserved G associated with ZF1 binding in CTCF’s binding motif identified in this study [51]. Their data, which was highly consistent with ours, supports and strengthens our findings, since independent approaches were used in each study. Moreover, our elegant AID2 system also allows the expression and functional study to shift from endogenous CTCF to any mutant forms, including those affecting cellular viability. We believe this new model system will significantly advance the understanding of CTCF biology in the future.

## Conclusions

By combining AID2 degradation of CTCF with induced exogenous expression of CTCF ZF1 and ZF10 mutants, we developed a new cellular tool to study the impact of CTCF domain mutants on CTCF function. Our findings provide the first direct support that ZFs 1 and 10 bind loci through the recognition of individual consensus sequence motifs highlighting loci regulated by CTCF contain different CTCF binding motifs dependent on how the ZF domain interacts with the DNA, supporting the “CTCF code” model of multivalent binding [52]. Moreover, this binding controls the transcription of a subset of genes regulated by CTCF, uncovering functional roles of the less conserved upstream and downstream motifs, and defining CTCF binding motifs for loci regulated by peripheral ZF binding. Future studies mutating additional ZFs and ZF combinations would further define CTCF’s binding specificities. While not performed in this study, utilization of the cellular model developed here could be combined with chromatin studies to examine how ZF mutants affect chromatin architecture and organization.

## Methods

### Vector construction

The pCDH-MND-OsTIR1(F74G)-P2A-EGFP^AID2^-EF1a-RFP construct was made by Gibson assembly [53]. The OsTIR1(F74G)-P2A-EGFP^AID2^ fragment was amplified from the pAAV-hSyn-OsTIR1(F74G) vector (Addgene, 140,730) and cloned into the EcoR1 site of pCDH-EF1a-RFP. After cloning, the CMV promoter was replaced by Gibson assembly with an MND promoter to overcome promoter silencing [54]. The inducible CTCF series was created by cloning the WT CTCF and CTCF dZF1, dZF10, and dRBR (− 120 bps following ZF11) into a Tet-on-3G-inducible vector. Primers were designed to amplify CTCF from a pT2K-CTCF-HA construct we had previously cloned. The mutants were generated by designing primers to amplify two fragments that flanked either ZF1, ZF10, or the RBR and would exclude these features after Gibson assembly. Snapgene software was used to design all primers used for cloning. The PCR reactions to amplify all products for cloning were performed using CloneAmp polymerase (Clontech) and the cycling parameters were 98 °C for 5 min, followed by 40 cycles of 98 °C for 15 s, 55 °C for 20 s, and 72 °C for 20 s. Gibson assembly reaction mix was made as previously described [53], and all reactions were carried out at 50 °C for 20 min. All primer information was included in the Additional file 2: Table S1.

### Generation of the CTCF^AID2^ cell line and cell culture

The CTCF^AID2^ cell line was created by infecting the pCDH-MND-OsTIR1(F74G)-P2A-EGFP^AID2^ EF1a RFP construct containing an EGFP AID-tagged control [33] into a previously derived SEM B-ALL cell line expressing the endogenous CTCF^AIDmClover3^ fusion protein and doxycycline-inducible wild-type (WT) OsTIR1 in the AAVS1 safe harbor locus (CTCF^AID1^) [12]. The SEM^OsTIR1(F74G)^ cell line was created by infecting the pCDH-MND-OsTIR1(F74G)-P2A-EGFP^AID2^ EF1a RFP construct containing an EGFP AID-tagged control into SEM^WT^ cells.

The SEM^WT^, SEM^OsTIR1(F74G)^, CTCF^AID1^, and CTCF^AID2^ cell lines were maintained in RPMI-1640 medium (Lonza) containing 10% fetal bovine serum (FBS) (Hyclone), 2 mM glutamine (Sigma), and 1% penicillin/streptomycin (Thermo Fisher Scientific). All cells were maintained at 37 °C in a 5% CO_2_ atmosphere and 95% humidity. Cells were tested negative for mycoplasma infection. The cell identity of SEM was confirmed by short tandem repeat (STR) analysis.

Growth assays were performed by plating 1 million cells in 3-mL RPMI medium supplemented with either DMSO or AID1 (500 μM IAA and 1 μg/mL doxycycline) or AID2 (10 μM 5-Ph-IAA) treatment conditions in a 6-well tissue culture plate. Samples were set up in triplicate. Cells were counted using a Countess II automated cell counter (Invitrogen) daily for 4 days. At each count, cells were submitted for cell cycle analysis.

### Auxin-induced degradation

CTCF^AID1^ cells were cultured in a medium supplemented with doxycycline (1 μg/mL) to induce the expression of OsTIR1 and 500 μM IAA (natural auxin) (Abcam) to induce degradation of CTCF. CTCF^AID2^ cells were cultured in a medium supplemented with 0.001–10 μM 5-Ph-IAA (MedChemExpress) to induce the degradation of CTCF. CTCF^AID2/WT^, CTCF^AID2/dZF1^, CTCF^AID2/dZF10^, and CTCF^AID2/dRBR^ cells were cultured in a medium supplemented with 10 μM 5-Ph-IAA to induce endogenous CTCF degradation for 6 h and 1 μg/mL doxycycline was added for 18 h to induce exogenous HA-tagged CTCF expression.

### Flow cytometry

To determine the percentage of RFP or mClover3-positive, suspension-cultured CTCF^AID2^ cells were collected and filtered through a 70-micron cell strainer before flow cytometry sorting. DAPI was added to the cell suspension to exclude dead cells. Fluorescence from mClover3 was detected using the same FL1/FITC channel as GFP.

### Immunoblotting

Cell lysates were prepared by using RIPA buffer. Lysates were run on an SDS-PAGE (Thermo Fisher Scientific) gel and transferred to a PVDF membrane according to the manufacturer’s protocols (Bio-Rad) at 100 V for 1 h. After blocking incubation with 5% non-fat milk in TBS-T (10 mM Tris, pH 8.0, 150 mM NaCl, 0.5% Tween-20) for 1 h at room temperature, membranes were incubated with antibodies against GAPDH (Thermo Fisher Scientific, AM4300, 1:10,000), AID (MBL, M3-214–3, 1:2000), OsTIR1 (MBL, PD048, 1:1000), or CTCF (Santa Cruz, sc-271514, 1:200) at 4 °C overnight with gentle shaking. Membranes were washed three times for 10 min each with TBS-T and incubated with a 1:2000 (CTCF), 1:5000 (AID), or 1:20,000 (GAPDH) dilution of sheep anti-mouse IgG HRP (GE Healthcare, NA931) or 1:5000 (OsTIR1) dilution of donkey anti-rabbit IgG HRP (GE Healthcare, NA340) for 1 h at room temperature. Blots were washed with TBS-T three times for 10 min each and developed with the ECL system (Perkin Elmer) according to the manufacturer’s protocol. Uncropped raw data were provided in Additional file 4.

### ChIP-seq

For CTCF ChIP-seq, CTCF^AID2^ cells were treated with 1 μM 5-Ph-IAA for 24 h. For HA ChIP-seq, CTCF^AID2^ cells with induced exogenous expression of HA-CTCF WT, HA-CTCF-dZF1, HA-CTCF-dZF10, and HA-CTCF-dRBR were treated for 24 h with 10 μM 5-Ph-IAA and 18 h with 1 μg/mL doxycycline. Treated and untreated samples were collected for all in duplicate. For each sample, 20 million cells were fixed with 1% formaldehyde for 5 min at room temperature using the Covaris TruChIP Chromatin Shearing Kit (Covaris, 520,154). Nuclei were prepared according to the TruChIP protocol and chromatin was sheared in a Covaris milli tube using the Covaris M220 ultrasonicator set at a duty factor of 10 and 200 cycles/burst for 10 min at set point 6 °C. Sheared chromatin was centrifuged for 10 min at 8000 × g and clarified chromatin was moved to a new 1.5-mL Eppendorf tube. Chromatin was amended to a final concentration of 50 mM Tris–HCL pH 7.4, 100 mM NaCl, 1 mM EDTA, 1% NP-40, 0.1% SDS, and 0.5% Na deoxycholate plus protease inhibitors (PI). For CTCF ChIP, 8 μg CTCF antibody (Diagenode, c15410210-50) was added to the chromatin and incubated overnight at 4 °C with gentle rotation. Spike-in chromatin and antibody were added to the chromatin according to the manufacturer’s protocol (Active Motif). The next day, protein-G magnetic beads (Pierce) were washed and added for 4 h incubation at 4 °C with gentle rotation. For HA ChIP, anti-HA magnetic beads (Pierce) were washed, added to the chromatin, and incubated at 4 °C for 4 h with gentle rotation (no spike-in included). Samples were placed on a magnetic stand, unbound chromatin was removed, and beads were washed 2 times with wash buffer 1 (50 mM Tris–HCL pH 7.4, 1 M NaCl, 1 mM EDTA, 1% NP-40, 0.1% SDS, 0.5% Na deoxycholate plus PI) and 1 time with wash buffer 2 (20 mM Tris–HCL pH 7.4, 10 mM MgCl_2_, 0.2% Tween-20 plus PI). The beads were resuspended in wash buffer 2 and transferred to a new 1.5-mL Eppendorf tube. Samples were placed on a magnetic stand to remove the wash buffer. DNA was eluted and de-crosslinked in 1X TE plus 1% SDS, proteinase K, and 400 mM NaCl at 65 °C for 4 h. DNA was precipitated by phenol, chloroform, and isopropyl alcohol. Libraries were constructed by NEBNext Ultra II NEB Library Prep Kit and NEBNext Multiplex oligos for Illumina.

### HiChIP

The HiChIP protocol was adapted from Mumbach et al. [55]. Ten million CTCF^AID2^ cells were treated with DMSO or 10 μM 5-Ph-IAA for 6 h. Cells were fixed with 2% formaldehyde for 10 min at room temperature. Fixed pellets were lysed in 500 μL of ice-cold HiC lysis buffer with PI (10 mM Tris–HCl pH 8.0, 10 mM NaCl, 0.2% Igepal CA630, 1X cOmplete PI) at 4 °C with rotation for 20 min followed by centrifugation at 2500 × g for 5 min at 4 °C. The pelleted nuclei were washed once with 500 μL of ice-cold HiC lysis buffer + PI, resuspended in 100 μL of 0.5% SDS, and incubated at 62 °C for 10 min with no shaking. After incubation, 285 μL of water and 50 μL of 10% Triton X-100 (Sigma, 93,443) were added and incubated at 37 °C for 15 min. Fifty microliters of 10X NEBuffer2 and 15 μL of 25 U/μL MboI restriction enzyme (375U) (NEB, R0147M) were added, and chromatin was digested on the Eppendorf thermomixer for 2 h at 37 °C with 900 rpm and a heated cover. To inactivate MboI, the reaction was incubated at 62 °C for 20 min, then cooled to room temperature. Restriction fragment overhangs were filled in and marked with biotin by adding 54 μL of fill-in master mix [37.5 μL of 0.4 mM biotin-14-dATP (Life Technologies, 19,524–016), 1.5 μL of 10 mM dCTP (Thermo Fisher, R0181), 1.5 μL of 10 mM dGTP (Thermo Fisher, R0181), 1.5 μL of 10 mM dTTP (Thermo Fisher, R0181), 12 μL of 5 U/μL DNA polymerase I, Large (Klenow) Fragment (NEB, M0210)] and incubated on Eppendorf thermomixer at 37 °C for 1 h and 15 min with shaking speed at 900 rpm. Ligation of proximity ends was carried out by adding 946 μL of ligation master mix [651.5 μL of water, 150 μL of 10X NEB T4 DNA ligase buffer (NEB, B0202), 125 μL of 10% Triton X-100, 7.5 μL of 20 mg/mL bovine serum albumin (NEB, B9000S), 12 μL of 400 U/μL T4 DNA ligase (NEB, M0202L)] and incubating on an Eppendorf thermomixer at 16 °C overnight with shaking speed at 900 rpm. Nuclei were collected by centrifugation, washed, and resuspended in Covaris shearing buffer plus PI and sheared in a Covaris milli tube at 30% duty factor, 200 cycles/burst for 10 min in a Covaris LE220-plus. Samples were clarified by centrifuge at 2500 rcf for 5 min at 4 °C, then the supernatant was transferred to a 15-mL DNA LoBind Conical Tube (Eppendorf, 0,030,122,208). About 3 mL of freshly made 1X ChIP dilution buffer + PI (21 mM Tris pH 8.0, 167 mM NaCl, 1 mM EDTA, 1.1% Triton X, 0.1% NP-40) was added to the pellet. Then, 60-μL washed DiaMag protein A/G magnetic beads were added and incubated at 4 °C for 1 h to preclear the lysates. Lysates and beads were separated by placing them on a magnetic stand and lysates were transferred to a new tube. CTCF antibody [8 μg Diagenode CTCF rabbit polyclonal antibody (C15410210-50)] was added and incubated at 4 °C overnight with rotation. Sixty microliters of Diagenode DiaMag protein A/G-coated magnetic beads was washed twice in 200 μL freshly made DiaMag beads wash buffer (1X ChIP dilution buffer + 0.1% BSA) and then resuspended in 1 mL of DiaMag beads wash buffer and rotated at 4 °C overnight. Blocked protein beads were added to the sample lysates and incubated with rotation at 4 °C for 3 h. Beads were washed one time with Cold Low Salt Wash Buffer (20 mM Tris pH 8.0, 150 mM NaCl, 2 mM EDTA, 1% Triton X, 0.1% SDS), 2 times with Cold High Salt Wash Buffer (20 mM Tris pH 8.0, 500 mM NaCl, 2 mM EDTA, 1% Triton X, 0.1% SDS), 1 time with Cold LiCl Wash Buffer (10 mM Tris pH 8.0, 250 mM LiCl, 1 mM EDTA, 1% NP-40, 1% Na deoxycholate), and 2 times with Cold TE Buffer, pH 8.0. DNA was eluted by resuspending the beads in 100 μL of DNA elution buffer (50 mM NaHCO3, 1% SDS) and incubating at room temperature for 10 min with rotation, followed by 3 min at 37 °C with shaking. Samples were placed on a magnetic stand and the supernatant was transferred to a new tube. The elution was repeated, and eluates combined. Samples were de-crosslinked by adding 10 μL of 20 mg/mL proteinase K and incubating at 55 °C for 45 min with shaking. The temperature was increased to 67 °C and samples were incubated for 1.5 h with shaking. DNA was extracted and eluted in 23 μL elution buffer using the Qiagen MinElute kit (Qiagen MinElute PCR Purification Kit, 28,006). For biotin pull-down, 15 μL of 10 mg/mL Dynabeads MyOne Streptavidin T1 beads (Life Technologies, 65,602) was washed with 150 μL of 1X Tween Washing Buffer [1X TWB: 5 mM Tris–HCl (pH 7.5); 0.5 mM EDTA; 1 M NaCl; 0.05% Tween 20]. After washing, the beads were resuspended in 150 μL of 2X binding buffer (2X BB: 10 mM Tris–HCl (pH 7.5); 1 mM EDTA; 2 M NaCl) and added to the DNA followed by incubation at room temperature for 15 min with rotation to bind biotinylated DNA to the streptavidin beads. Beads were separated on a magnetic stand and washed by adding 400 μL of 1X TWB and transferring the mixture to a new tube. The tubes were heated on a Thermomixer at 55 °C for 2 min with 950 rpm mixing. The wash was repeated one more time followed by one wash using 200 μL of 1X Tris buffer. After washing, the beads were resuspended in 50 μL 1 × Tris buffer. The Roche Kapa HyperPrep Kit (KK8502/KK8504) and Illumina TruSeq DNA UD Indexes were used to prepare library DNA.

### HiChIP/HiC data analysis

For HiChIP, paired-end reads of 151 bp were confirmed for the enrichment of MboI ligation sites (GATCGATC) and trimmed for adapters by fastp (version 0.20.0, paired-end mode, parameter as “–detect_adapter_for_pe –trim_poly_x –cut_by_quality5 –cut_by_quality3 –cut_mean_quality 15 –length_required 20 –low_complexity_filter –complexity_threshold 30”) [56]. Then, trimmed reads were processed by HiC-Pro (version 2.11.4) [57] using human genome hg19 (GRC37 from GENCODE) and MboI fragment file (cut site GATC). Bowtie2-2.2.4, samtools-1.2, R-3.4.0, and Python-2.7.12 were configured for HiC-Pro. allValidPairs files from the HiC-Pro pipeline were then used to generate.hic file for visualization. Both samples have 2 biological replicates with good depth (~ 150 M pairs) and comparable metrics with the published data (GEO id: GSE80820) [55] such as a valid interaction rate (95.6 to 97.8% compared to GEO 78.65 to 80.53%). After confirmation of reproducibility by HiC-Spector [58] and HiCRep [59], contacts from replicates were merged and call loops using FitHiChIP (Stringent mode using Coverage for normalization) [60] based on CTCF peaks from GSM3312803 [61]. We also confirmed loops mostly overlapped CTCF peaks and convergent CTCF motif patterns.

For HiC, the pair-end reads of 76-bp reads were processed by Juicer (v1.5, default parameters) [62] based on hg19 and MboI fragment (ligation sites GATCGATC). Eight replicates were processed separately, and reproducibility was confirmed by HiC-Spector [58] and HiCRep [59] (see code repository https://doi.org/10.6084/m9.figshare.21002533 for details). Then, we merged all replicates to reach the highest resolution of HiC data for SEM cells (2.7 billion reads, 2 billion contacts). We called 6720 loops by HiCCUPS from the Juicer package (parameters: “-r 5000,10,000,25,000 -f 0.2,0.2,0.2 -p 4,2,1 -i 7,5,3 -t 0.05,1.25,1.25,1.5 -d 20,000,20,000,50,000”) and confirmed they mostly overlapped CTCF peaks and convergent CTCF motif pattern.

APA scores refer to the ratio of the mean central pixels to the mean of pixels in the lower left corner (a.k.a P2LL) in Aggregate Peak Analysis [6]. We used the APA function from Juicer pipeline [62] for the analysis at 5-kb resolution and then plotted using normalized signal (“normedAPA” version from Juicer APA output, https://github.com/aidenlab/juicer/wiki/APA). For APA analysis in Fig. 1m, we included the CTCF HiChIP signal for − 5-Ph-IAA and + 5-Ph-IAA loops at all 7220 loops called in CTCF HiChIP of − 5-Ph-IAA.

### SLAM-seq

The SLAM-seq protocol was adapted from Herzog et al. 2018 Protocol Exchange and Muhar et al. [38, 63]. CTCF^AID1^ or CTCF^AID2^ cells were treated with either 500 μM IAA, 1 μg/mL doxycycline (AID1), or 10 μM 5-Ph-IAA (AID2) for 2, 4, 6, 12, or 24 h. For the last hour of treatment, 250 μM 4SU was added to the media. Cells not treated with auxin received a 1-h treatment of 250 μM 4SU for labeling. After 4SU labeling, cells were collected under restricted light. Total RNA was extracted by Trizol in a dark room. The reaction conditions for thiol modification were 3 μg RNA, 10 mM iodoacetamide (made fresh by dilution in 100% ETOH), 50 mM NaPO_4_, and 50% DMSO. The reaction was incubated at 50 °C for 15 min and 1 μL 1 M DTT was added to stop the reaction. RNA was purified using the RNA clean and concentrator kit from Zymo Research and libraries were prepared using the Quant-seq 3′ end mRNA library prep kit for Illumina (Lexogen).

### SLAM-seq data analysis

Single-end 51-cycle sequencing was performed using the NovaSeq 6000 platform following the manufacturer’s instructions (Illumina). Quantifying 3′ UTR T > C reads was performed using SlamDunk (v0.4.2) [38] software according to the protocol (http://t-neumann.github.io/slamdunk/docs.html, using the command “slamdunk all” with default options) with primary assembly GRCh38.p12 and Gencode annotation v31 3′ UTR definitions. Only non-overlapping 3′ UTRs > 10 bp were retained. For each sample, T > C counts were collapsed by the gene of origin, using the SlamDunk module “alleyoop collapse”; genes with CPM ≤ 10 were removed from downstream analyses. Conversion rates were quantified by slamdunk module “alleyoop rates.” Package edgeR (v3.24.3) [64] in the R environment (v3.5.1) was used to normalize gene expression with function calcNormFactors (method = “TMM”). Differentially expressed genes were called using the trimmed mean of *M* values (TMM) normalization factors and raw counts in a Limma-voom analysis using the “voom,” “lmFit,” and “eBayes” functions from the limma (v3.42.2) R package, with statistical significance threshold *p *value ≤ 0.05 and |log_2_(FC)|≥ 1 [65]. Gene set enrichment analyses were performed using gseapy (version 10.4; method = “signal_to_noise”), a pythonic wrapper/implementation of GSEA [66], with the normalized gene expression values from samples of two treatment arms and the hallmark gene sets from MSigDB (v7.2) [67]. The principal component analysis (PCA) was performed using the TMM normalized log_2_(CPM) (counts per million) and ranked (descending) using the median absolute deviation as implemented by the “mad” function in R. The top 3000 most variable genes were used to perform the analysis. Both the PCA and the visualization were performed using the R package PCAtools v2.3.7, first using the function “pca” with default values. The first two principal components (PC1 and PC2) were visualized using the function “biplot.”

### CTCF domain library design and drop-out screen

A total of 512 20-bp sgRNAs were designed by FlashFry (version 1.12) [68] to span all CTCF exons. Low-quality sgRNAs (extreme GC content, polyT, non-unique, Hsu2013 score ≤ 55, or DoenchCFD_specificityscore ≤ 0.02) were excluded [69, 70]. One hundred and twenty positive control sgRNAs and 100 negative control sgRNAs were also included. The oligonucleotides for the sgRNAs designed were synthesized by CustomArray. Forward library PCR primer (5′-GGCTTTATATATCTTGTGGAAAGGACGAAACACC-3′) (10 μM) and reverse library PCR primer (5′-CTAGCCTTATTTTAACTTGCTATTTCTAGCTCTAAAAC-3′) (10 μM) were used to amplify the sgRNA oligonucleotides using 2X HiFi CloneAmp PCR mixture (Clontech) under the following PCR conditions: 98 °C 3 min, 98 °C 10 s, 55 °C 10 s, 72 °C 10 s, 72 °C 5 min, and 4 °C hold for 12 cycles. The amplified product was run on a SybrGreen stained 2% agarose gel and bands were excised for gel purification by a Qiagen Gel Purification kit. The amplified sgRNAs (10 ng) were cloned into the LentiGuide-Puro (#52,963) backbone cut by BsmB1 (100 ng) using the NEbuilder HiFi DNA assembly master mix (NEB) at 50 °C for 1 h. Eight 50-μL vials of NEB stable competent *E.* *coli* high-efficiency cells (NEB, C3030H) were thawed on ice and 2 μL of the assembled reaction was added to each. Cells were incubated on ice for 30 min, heat shocked at 42 °C for 30 s, and then placed on ice. NEB 10-beta/stable outgrowth medium was added to the heat-shocked cells (950 mL per vial) and incubated at 30 °C for 60 min at 250 rpm. Recovered cells were plated at 2.5 mL per square LB + ampicillin dish (245 mm × 245 mm) and incubated at 30 °C overnight. Bacterial colonies were counted to ensure good library coverage and collected for DNA extraction of the pooled sgRNA library by Qiagen Maxi prep (Qiagen).

CTCF^AID2^ cells stably expressing lentiviral Cas9-blasticidin were infected with the pooled sgRNA library at low M.O.I (~ 0.3). Infected cells were selected with blasticidin and puromycin for 3 days. On days 7 and 14 post-antibiotic selection, cells were collected and the sgRNA sequences were recovered by genomic PCR analysis (Additional file 2: Table S1, Nextera primers), indexed (Nextera, FC-131–1096), and sequenced using NovaSeq 6000 for single-end 151 bp read length (Illumina). The sgRNA sequences are described in Additional file 3: Table S2. High-titer lentivirus stocks were generated in 293 T cells as previously described [71].

### CRISPR-Cas9 tiling-sgRNA knockout screen data analysis

Raw data 151-bp reads were obtained from Illumina NovaSeq and trimmed for adapters. Then, we counted 20mer by bbmap (version 37.28, “kmercountexact.sh fastadump = f mincount = 1 k = 20 rcomp = f”) and assigned to sgRNAs. MAGeCK (version 0.5.9.4, default parameters) [72] was used for statistical analysis and results were then extracted to make protein domain-based plots using Protiler (version 1.0.0) [39].

### RNA-seq

Total RNA was extracted by Trizol (Thermo Fisher Scientific, 15,596,026) from replicate samples of SEM^WT^ and CTCF^AID1^ cells treated with either DMSO or 500 μM IAA, 1 μg/mL Dox for 1 day, and CTCF^AID2^ cells treated with either DMSO or 10 μM 5-Ph-IAA for 1, 4, and 7 days. About 200 ng total RNA was treated using Kapa rRNA depletion reagents to remove ribosomal RNA, then converted into cDNA libraries using Kapa RNA HyperPrep Kit with RiboErase (HMR). After end repair, dA-tailing, and adapter ligation, each cDNA library was purified and enriched by 11 cycles of PCR amplification.

### RNA-seq data analysis

Paired-end 101-cycle sequencing was performed on the NovaSeq 6000 sequencer following the manufacturer’s instructions (Illumina). Raw reads were first trimmed using TrimGalore (v0.6.3) with parameters “–paired –retain_unpaired.” Filtered reads were then mapped to the *Homo sapiens* reference genome GRCh37.p13 using STAR (v2.7.9a) [73]. Gene-level read quantification was done using RSEM (v1.3.1) [74] on the Gencode annotation v19 [75]. To identify the differentially expressed genes, normalization factors were first estimated using the TMM and genes with CPM ≤ 1 in all samples were removed. Next, the TMM normalization factors and raw counts were then used for the Limma-voom analysis using the “voom,” “lmFit,” and “eBayes” functions from the limma R package [65]. Gene set enrichment analysis (GSEA) was performed using the MsigDB database (v7.1). Differentially expressed genes were ranked based on their log_2_(FC) [76]. The principal component analysis (PCA) plots were generated from the TMM normalized data. Based on the log_2_(CPM) data, we ranked the genes based on their median absolute deviation (using the “mad” function in R) as it is a more robust statistic against outliers. The log_2_(CPM) of the top 3000 variable genes was passed to the “prcomp” function to do PCA analysis. The first two principal components were used to generate the PCA plots.

### ChIP-seq data analysis

Single-end 51-cycle sequencing was performed on the NovaSeq 6000 sequencer following the manufacturer’s instructions (Illumina). Raw reads were first trimmed using TrimGalore (v0.6.3). Filtered reads were then aligned using BWA (v0.7.17-r1198) [77] to the *Homo sapiens* reference genome GRCm37.p13 or to a hybrid-genome between the human GRCm37.p13 genome and the *Drosophila melanogaster* (dm6) genome if they have spike-in materials. Duplicated reads were marked using the “bamsormadup” function from the biobambam2 tool (v2.0.87) (https://gitlab.com/german.tischler/biobambam2). PCR duplicates and low mapping quality reads (MAPQ ≤ 1) were removed using samtools (version 1.9, parameter “-q 1 -F 1024”) [78]. Human and *Drosophila* reads were then extracted into two separated bam files. The uniquely mapped reads in the human genome were then used to estimate the average fragment length in each sample based on the cross-correlation profile calculated from SPP (v1.11) [79]. The smallest fragment size estimated by SPP was used to center and extend reads to generate bigwig files. If the samples did not contain spike-in materials, the bigwig signals were scaled to 15 million reads (i.e., scaling-factor = 15e6/libSize). If the samples contained spike-in, the bigwigs were generated by scaling the number of uniquely mapped spike-in reads to 1 million reads (i.e., scaling-factor = 1e6/spike-in_counts). Macs2 (v 2.1.1) was used to call peaks using parameters “-g hs –nomodel –extsize < SPP_fragmentSize > ” [80]. Two sets of peaks were generated: (i) “high confidence peaks”: called with FDR ≤ 0.05 (parameter “-q 0.05”) and (ii) “low confidence peaks” called with FDR ≤ 0.5 (parameter “-q 0.5”). We consider a peak to be reproducible if it was called as a high confidence peak in at least one replicate that also overlapped with a low confidence peak in the other replicates.

### Differential ChIP-seq peak analysis

Reproducible peaks from treated and untreated cells were merged together. For each peak, we counted the number of overlapping ChIP-seq fragments generated for the paired reads based on the estimated fragment size from SPP (slopBed -s -l 0 -r < SPP_fragmentSize >) in each sample (bedtools v2.24.0) [81]. For the HA-CTCF-ZF mutants, counts were normalized using the trimmed mean of *M* values (TMM) method. For the CTCF AID2 samples, were normalized based on the median of the ratios of observed counts of spike-in [82]. We identified the differential peaks using the empirical Bayes method (eBayes function from the limma R package) [65]. For downstream analyses, heatmaps were generated by deepTools [83]. Peaks were annotated based on Gencode v19 genes coordinates following this priority: “Promoter.Up”: if they fall within TSS − 2 kb, “Promoter.Down”: if they fall within TSS − 2 kb, “Exonic” or “intronic”: if they fall within an exon or intron of any isoform, “TES peaks”: if they fall within TES ± 2 kb, “distal5” or “distal3” if they are with 50 kb upstream of TSS or 50 kb downstream of TES, respectively, and they are classified as “intergenic” if they do not fit in any of the previous categories.

For CTCF flanking motif analysis, we first scanned the CTCF motif (TRANSFAC M01259) by FIMO (MEME suite 5.3.3, “–thresh 1e-4”) and retrieved sequence ± 20 bp flanking the motif matches. We then generated a heatmap by seaborn (v0.11.1) using hierarchical clustering (hamming distance) from scipy (v1.6.2), and the motif consensus logo was generated by logomaker (v0.8).

### Integrative analysis of RNA-seq and ChIP-seq changes

To identify differential CTCF peaks correlated with gene expression changes after CTCF depletion and exogenous CTCF HA mutant expression, we adapted some ideas from the intePareto method [84]. For each gene $g$, we converted its RNA-seq log_2_(FC) to a *z*-score by scaling the log_2_(FC) to the standard deviation of all fold changes in the sample using the following formula:

$${z}_{g}=\frac{{\mathrm{log}}_{2}\mathrm{FC}\left(g\right)}{sd({\mathrm{log}}_{2}\mathrm{FC})}$$

Instead of associating a single peak to each gene as it was done in the original intePareto method, we associated a gene to all peaks in its genomic neighborhood defined as [TSS_g_ − 50 kb, TES_g_ + 50 kb] to be able to unbiasedly identify the most correlated peak. Similarly, we converted the fold change value of each peak $p$ in [TSS_g_ − 50 kb, TES_g_ + 50 kb] to a *z*-score using the same formula but using ChIP-seq fold change values:

$${z}_{p}=\frac{{\mathrm{log}}_{2}\mathrm{FC}\left(p\right)}{sd({\mathrm{log}}_{2}\mathrm{FC})}$$

For each gene-peak pair, we calculated a combined *z*-score by multiplying their *z*-scores as follows (Fig. 5a):

$${z}_{g,p}={z}_{g}\times {z}_{p}$$

The multi-objective Pareto optimization [https://ieeexplore.ieee.org/abstract/document/1599245] was then calculated using the “psel” function from the “rPref” R/package (v1.3) [https://journal.r-project.org/archive/2016/RJ-2016-054/index.html]. The peaks from the top 10 best Pareto levels were selected as the most correlated/anti-correlated.

Statistical analysis was done using R (v4.0.1), python 3.6, or GraphPad Prism software version 9. Heatmaps were generated using the pheatmap R/package. The ChIP-seq heatmaps were generated using deepTools (v3.5.0).

## Supplementary Information

**Additional file 1.**

**Additional file 2.**

**Additional file 3.**

**Additional file 4.**

**Additional file 5.**

## Data Availability

Data generated in this study, including total RNA-seq, nascent RNA-seq, ChIP-seq, and HiChIP, were deposited at NCBI GEO (Super-series GSE205218) [85]. Publicly available datasets (GSE80820 and GSM3312803) were referenced [86, 87]. Code repositories collected at https://doi.org/10.6084/m9.figshare.c.6186670 included ChIP-seq QC (https://doi.org/10.6084/m9.figshare.21002533), Integrative analysis ChIP-seq and RNA-seq (https://doi.org/10.6084/m9.figshare.21045889), Hi-C and HiChIP analysis (https://doi.org/10.6084/m9.figshare.21002533), and SLAM-seq analysis (https://doi.org/10.6084/m9.figshare.21259278) [88–92].
