# Supplementary material for: Auxin-inducible degron 2 system deciphers functions of CTCF domains in transcriptional regulation
Source: Genome Biol. 2023 Jan 26;24:14. doi: 10.1186/s13059-022-02843-3 (PMC9878928; doi:10.1186/s13059-022-02843-3)
Supplement: Supplementary file 1 — Additional file 1. [file 13059_2022_2843_MOESM1_ESM.pdf]

Auxin-inducible degron 2 system deciphers functions of CTCF domains in transcriptional regulation

Judith Hyle[1, *], Mohamed Nadhir Djekidel[2, *], Justin Williams[1], Shaela Wright[1], Ying Shao[3], Beisi Xu[2, #], Chunliang Li[1, #]

[1]Department of Tumor Cell Biology, St. Jude Children's Research Hospital, 262 Danny Thomas Place, Memphis, TN 38105, USA.

[2]Center for Applied Bioinformatics, St. Jude Children's Research Hospital, 262 Danny Thomas Place, Memphis, TN 38105, USA.

[3]Department of Computational Biology, St. Jude Children's Research Hospital, 262 Danny Thomas Place, Memphis, TN 38105, USA.

* Contributed equally

[#] Correspondence should be addressed to Dr. Chunliang Li (chunliang.li@stjude.org) or Dr. Beisi Xu (beisi.xu@stjude.org)

Fig. S1. Characterization of protein degradation of CTCF^{AID2} cells and impact on survival.

(a) Immunoblot analysis from CTCF^{AID2} cells following 0-24 hours 10 μM 5-Ph-IAA treatment; CTCF^{AIDmClover3} was detected by a CTCF antibody and EGFP^{AID2} was detected by a miniAID antibody.

(b) Immunoblot analysis of CTCF^{AID2} cells treated with a 1 μM 5-Ph-IAA titration for 6 hours. (

(c) Immunoblot analysis of CTCF^{AID2} cells co-treated with 1 μM 5-Ph-IAA and proteasome inhibitor MG132 for 5 hours.

(d) Immunoblot analysis of SEM^{WT} and CTCF^{AID1} cells treated with 500 μM IAA, 1μg/ml doxycycline, or a combination of both for 24 hours. The lower CTCF band represents untagged endogenous CTCF. The higher CTCF band represents the miniAID-tagged endogenous CTCF (CTCF^{AIDmClover3}).

(e) Growth assay of SEM^{WT} cells treated with DMSO, 500 μM IAA, 1μg/ml doxycycline, or a combination of both over 4 days. Samples were set up in triplicate and cell counts for each replicate were collected daily. Bars represent cell numbers (x millions).

(f) Growth assay of CTCF^{AID1} cells treated with DMSO, 500 μM IAA, 1μg/ml doxycycline, or a combination of both over 4 days. Samples were set up in triplicate and cell counts for each replicate were collected daily. Bars represent cell numbers (x millions).

(g) Growth assay of SEM^{WT}, SEM$^{OsTIR1(F74G)}$ and CTCF^{AID2} cells treated with DMSO or 1μM 5-Ph-IAA over 4 days. Samples were set up in triplicate and cell counts for each replicate were collected daily. Bars represent cell numbers (x millions).

Fig. S2. Characteristics of CTCF lost and resistant peaks after AID2 depletion.

(a) CTCF binding enrichment heatmap centered at CTCF peaks showing the binding intensity of CTCF peaks before and after 5-Ph-IAA treatment. Two main groups are identified: i) 27,262 CTCF

peaks that significantly reduced in intensity but were not completely lost, and ii) 19,291 CTCF peaks that were completely lost after 5-Ph-IAA treatment. An additional 67 peaks identified in AID2-treated cells are not shown in the heatmap due to low confidence.

(b) Meta plot showing the mean CTCF peak intensity in untreated cells of the lost and significantly reduced peaks.

(c) Stacked Bar-plot showing the genomic distribution of CTCF peaks from panel a.

(d) Genomic distribution of CTCF peaks from c) with a more stringent cutoff (FDR $\leqslant$ 0.05, ($|\log_2(FC)| \geqslant 1$).

(e) Boxplot plot showing the genomic distance of lost and significantly reduced CTCF peaks to nearby domain boundaries identified by HiChIP.

Fig. S3. QC analysis of HiC and HiChIP.

(a) Stratum-adjusted correlation coefficient at 100-kb resolution of six SEM Hi-C replicates [12], two publicly available SEM Hi-C data sets (GSM4120559, GSM4120560), and CTCF HiChIP with and without 6 hours 10 μM 5-Ph-IAA treatment (2 replicates each). The values plotting was median across all chromosomes calculated by HiCRep R package. The higher value indicates more reproducible events.

(b) Venn-diagram overlapping HiC (called by HiCCUPS from Juicer pipeline, see method) and CTCF HiChIP loops with and without 10 μM 5-Ph-IAA treatment for 6 hours (called by FitHiChIP, see method).

(c) Stacked Bar-plot showing the loops based on anchors distribution for retained (368), lost (6,852) or new (355) loops. Each loop anchor was assigned to one out of three groups, with the

following annotation, as Promoter (TSS +/- 2kb), Enhancer (SEM H3K27ac peak summit from GSM1934089) or CTCF.

(d) Boxplot with jitter for log2(fold change) of CTCF ChIP signal (+5-Ph-IAA versus -5-Ph-IAA) at CTCF peaks either overlap lost loops anchors or retained loops anchors. T-tests were used for the statistical test.

Fig. S4. QC analysis and differential gene analysis of SLAM-seq.

(a) Box plot of the percentage conversion rate of A>G (orange box, negative strand) and T>C (blue box, positive strand) quantified in each SLAM-seq experiment by SlamDunk using either AID^1 or AID^2 for CTCF depletion.

(b) Volcano plot representing the differential gene distribution for each time point in the AID^1 and AID^2 SLAM-seq time course. The horizontal dotted line represents a *p-value* of 0.05, and vertical lines represent the $\log_2$FC of –1.0 and 1.0. TCCPM = T>C CPM reads.

Fig. S5. Characterization of CTCF-independent effect in CTCF^{AID1} system.

(a) MA-plot of SEM^{WT} cells following AID1 treatment (500 μM IAA; 1 μg/ml doxycycline) for 24 hours. The $\log_2$ average mean expression is shown on the x-axis and Log_2(FC) is shown on the y-axis. The most significant differentially expressed genes ($|\log_2(\text{FC})| \geqslant 1$, FDR $\leqslant 0.05$) are shown as red dots.

(b) Differential expression of the *ASNS*, *DDIT3* and *TRIB3* genes by RNA-seq (FKPM) in response to 24 hours AID1 treatment in SEM^{WT} cells.

(c) GSEA plot of the top three enriched pathways following 24 hours of AID1 treatment in SEM^{WT} cells.

(d) Scatter plot showing the correlation between the 24h transcriptional changes of treated SEM^{WT} (WT) and AID1-treated $CTCF^{AID1}$. The x-axis shows the $log_{2(}FC)$ of SEM^{WT} cells after IAA treatment for 24 hours. The y-axis represents the log_2(FC) of $CTCF^{AID1}$ cells after IAA treatment for 24 hours.

(e) Scatter plot showing the low correlation between 24h transcriptional change of treated AID1 SEM^{WT} (WT) and AID2-treated (1 μM 5-Ph-IAA) $CTCF^{AID2}$ cells. The x-axis shows the log_2(FC) of SEM^{WT} cells after IAA treatment for 24 hours. The y-axis represents the log_2(FC) of $CTCF^{AID2}$ cells after 5-Ph-IAA treatment for 24 hours.

(f) Scatterplots showing the correlation between 24h transcriptional change of AID1-treated $CTCF^{AID1}$ cells and AID2-treated $CTCF^{AID2}$ cells. The y-axis represents the log_2(FC) of $CTCF^{AID1}$ cells after IAA treatment for 24 hours. The y-axis represents the log_2(FC) of $CTCF^{AID2}$ cells after 5-Ph-IAA treatment for 24 hours. d-f) Both Spearman and R^2 correlation values are shown.

Fig. S6. Characterization of CTCF binding signature of WT and mutants.

(a) Heatmap of normalized CTCF signal centered at CTCF peaks identified from 2 replicates of HA ChIP-seq of $CTCF^{AID2/WT}$, $CTCF^{AID2/dZF1}$, $CTCF^{AID2/dZF10}$, and $CTCF^{AID2/dRBR}$ cells following 10 μM 5-Ph-IAA treatment for 24 hours and 18 hours of 1 µg/mL doxycycline. A combined count of 27,896 peaks was identified in all conditions.

(b) Genomic distribution of all CTCF peaks from $CTCF^{AID2/WT}$. A total of 27,923 peaks were called.

(c) Genomic distribution of CTCF binding peaks decreased (⩾2 fold, FDR ⩽ 0.05) comparing $CTCF^{AID2/dZF10}$ to $CTCF^{AID2/WT}$.

(d) Genomic distribution of CTCF binding peaks increased (⩾2 fold, FDR ⩽ 0.05) comparing $CTCF^{AID2/dZF10}$ to $CTCF^{AID2/WT}$.

(e) Genomic distribution of CTCF binding peaks decreased ($\geqslant$2 fold, FDR $\leqslant$ 0.05) comparing $CTCF^{AID2/dZF1}$ to $CTCF^{AID2/WT}$.

(f) Genomic distribution of CTCF binding peaks increased ($\geqslant$2 fold, FDR $\leqslant$ 0.05) comparing $CTCF^{AID2/dZF1}$ to $CTCF^{AID2/WT}$.

(g) Genomic distribution of CTCF binding peaks decreased ($\geqslant$2 fold, FDR $\leqslant$ 0.05) comparing $CTCF^{AID2/dRBR}$ to $CTCF^{AID2/WT}$.

(h) Genomic distribution of CTCF binding peaks increased ($\geqslant$2 fold, FDR $\leqslant$ 0.05) comparing $CTCF^{AID2/dRBR}$ to $CTCF^{AID2/WT}$.

Fig. S7. Correlation analysis of DNA-binding and transcriptome changes in $CTCF^{AID2}$ cells.

(a)-(c) Schematic diagram of the integrative analysis of RNA-seq and differential binding of HA-CTCF-ZF mutants ChIP-seq. For each gene, we considered all the peaks within 50kb of TSS or TES. The RNA-seq and ChIP-seq fold-changes were converted to z-score, then multiplied together for each gene-peak pair to get a combined score. Pareto optimization was performed to determine correlated peak-gene pairs. Gene cutoff: DB: $|\log_2(FC)| \geqslant 1$ and FDR $\leqslant$ 0.05; DE: $|FC| \geqslant 2$ and FDR $\leqslant$ 0.05. The DE/DB pairs that passed $|\log_2(FC)| \geqslant 1$ FDR$\leqslant$0.05 were highlighted in orange.

(d) Boxplot showing the absolute differential binding $\log_2(FC)$ of the peaks of each HA-CTCT-ZF mutant identified by the integrative analysis. P-values were calculated using the Wilcoxon rank test.

(e) Venn-diagram illustrating the DE/DB pair genes in $CTCF^{AID2/dZF1}$, $CTCF^{AID2/dRBR}$ and $CTCF^{AID2/dZF10}$ compared with $CTCF^{AID2/WT}$ had little overlap.

Fig. S1

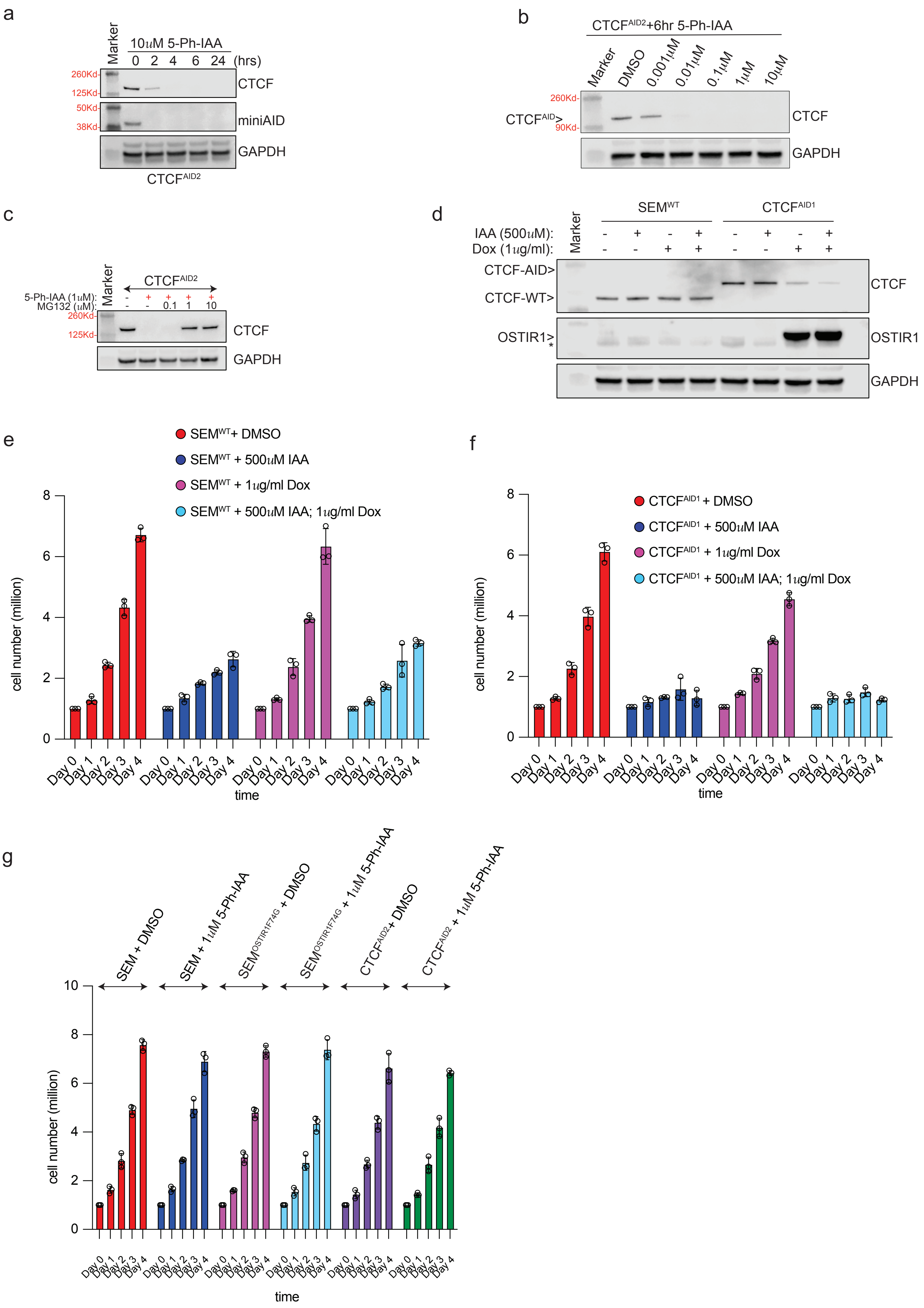
a
Marker
10uM 5-Ph-IAA
0 2 4 6 24 (hrs)
260Kd-
125Kd-
50Kd-
38Kd-
CTCF
miniAID
GAPDH
CTCFAID2
b
CTCFAID2+6hr 5-Ph-IAA
Marker DMSO 0.001uM 0.01uM 0.1uM 1uM 10uM
260Kd-
CTCFAID>
90Kd-
CTCF
GAPDH
c
Marker
CTCFAID2
5-Ph-IAA (1uM): - + + + +
MG132 (uM): - - 0.1 1 10
260Kd-
125Kd-
CTCF
GAPDH
d
SEMWT
CTCFAID1
Marker
IAA (500uM): - + - + - + - +
Dox (1ug/ml): - - + + - - + +
CTCF-AID>
CTCF-WT>
CTCF
OSTIR1>
OSTIR1
GAPDH
e
SEMWT+ DMSO
SEMWT + 500uM IAA
SEMWT + 1ug/ml Dox
SEMWT + 500uM IAA; 1ug/ml Dox
cell number (million)
time
f
CTCFAID1 + DMSO
CTCFAID1 + 500uM IAA
CTCFAID1 + 1ug/ml Dox
CTCFAID1 + 500uM IAA; 1ug/ml Dox
cell number (million)
time
g
SEM + DMSO
SEM + 1uM 5-Ph-IAA
SEMOSTIR1F74G + DMSO
SEMOSTIR1F74G + 1uM 5-Ph-IAA
CTCFAID2+ DMSO
CTCFAID2 + 1uM 5-Ph-IAA
cell number (million)
time
Day 0 Day 1 Day 2 Day 3 Day 4

Fig. S2

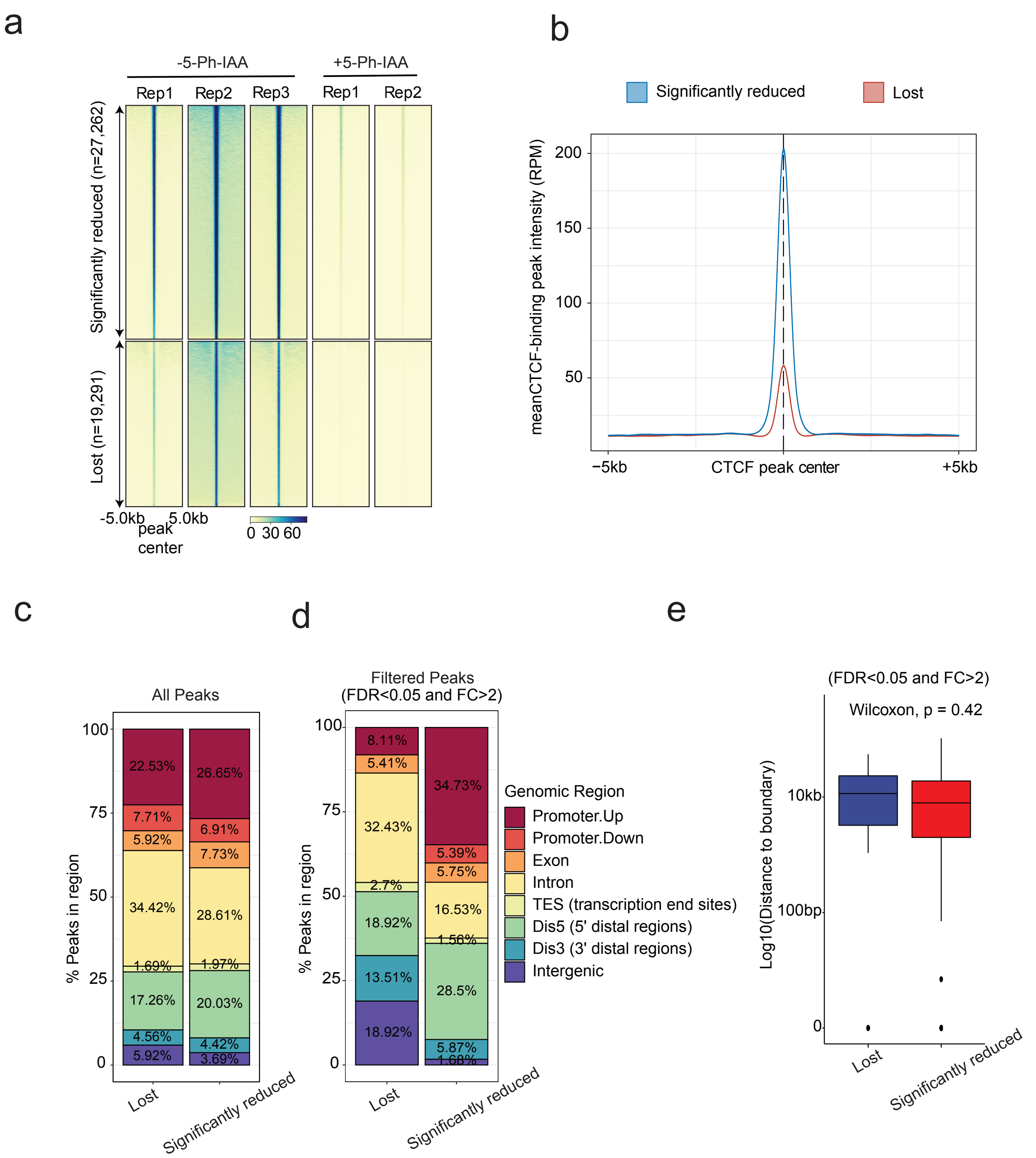

a
-5-Ph-IAA
+5-Ph-IAA
Rep1
Rep2
Rep3
Rep1
Rep2
Significantly reduced (n=27,262)
Lost (n=19,291)
-5.0kb
peak center
5.0kb
0 30 60
b
Significantly reduced
Lost
meanCTCF-binding peak intensity (RPM)
200
150
100
50
−5kb
CTCF peak center
+5kb
c
All Peaks
% Peaks in region
100
75
50
25
0
22.53%
7.71%
5.92%
34.42%
1.69%
17.26%
4.56%
5.92%
26.65%
6.91%
7.73%
28.61%
1.97%
20.03%
4.42%
3.69%
Lost
Significantly reduced
d
Filtered Peaks
(FDR<0.05 and FC>2)
% Peaks in region
100
75
50
25
0
8.11%
5.41%
32.43%
2.7%
18.92%
13.51%
18.92%
34.73%
5.39%
5.75%
16.53%
1.56%
28.5%
5.87%
1.68%
Lost
Significantly reduced
Genomic Region
Promoter.Up
Promoter.Down
Exon
Intron
TES (transcription end sites)
Dis5 (5' distal regions)
Dis3 (3' distal regions)
Intergenic
e
(FDR<0.05 and FC>2)
Wilcoxon, p = 0.42
Log10(Distance to boundary)
10kb
100bp
0
Lost
Significantly reduced

Fig. S3

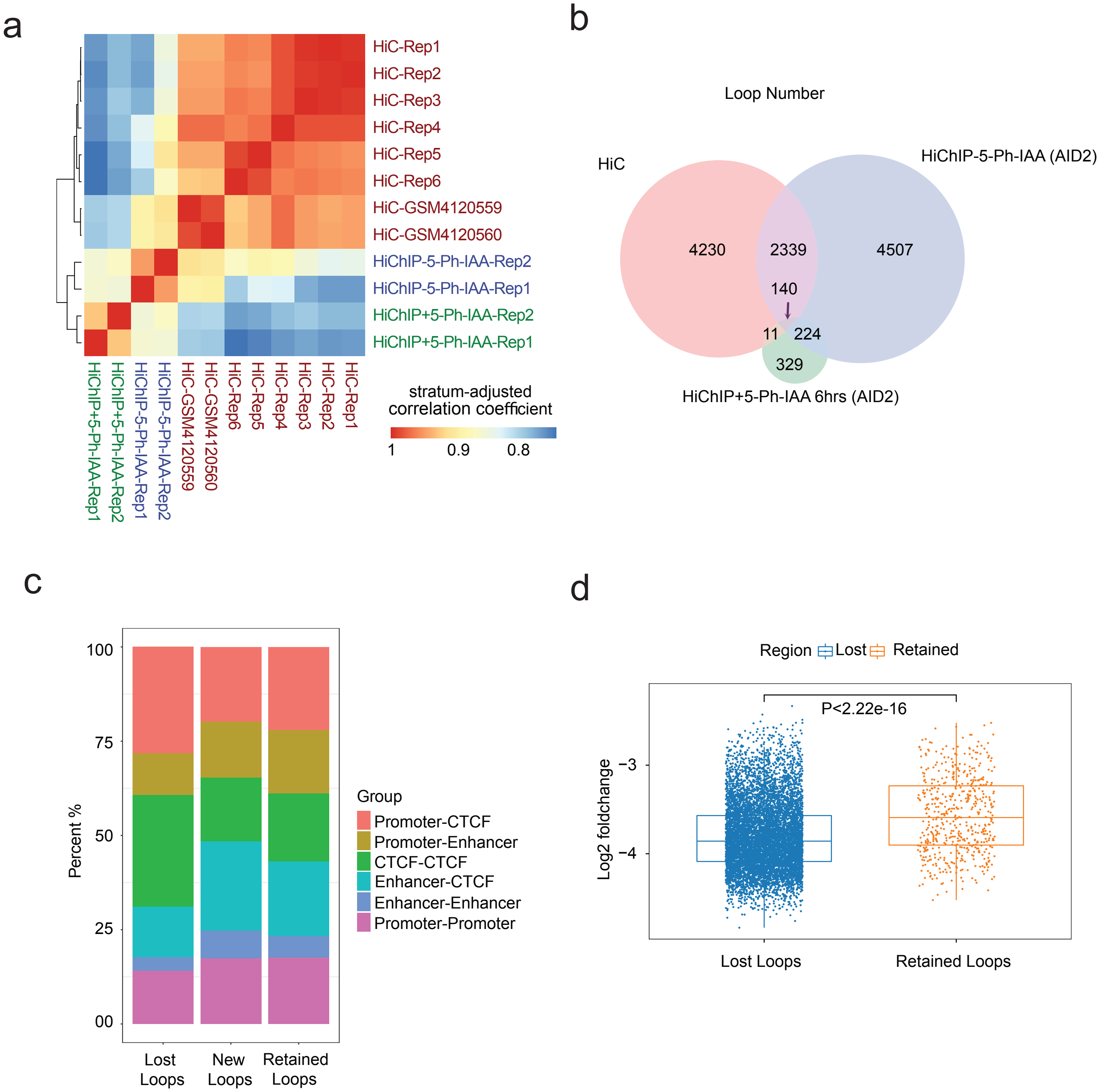
a
HiC-Rep1
HiC-Rep2
HiC-Rep3
HiC-Rep4
HiC-Rep5
HiC-Rep6
HiC-GSM4120559
HiC-GSM4120560
HiChIP-5-Ph-IAA-Rep2
HiChIP-5-Ph-IAA-Rep1
HiChIP+5-Ph-IAA-Rep2
HiChIP+5-Ph-IAA-Rep1
stratum-adjusted
correlation coefficient
1
0.9
0.8
b
Loop Number
HiC
HiChIP-5-Ph-IAA (AID2)
4230
2339
4507
140
11
224
329
HiChIP+5-Ph-IAA 6hrs (AID2)
c
Percent %
100
75
50
25
00
Lost Loops
New Loops
Retained Loops
Group
Promoter-CTCF
Promoter-Enhancer
CTCF-CTCF
Enhancer-CTCF
Enhancer-Enhancer
Promoter-Promoter
d
Region
Lost
Retained
P<2.22e-16
Log2 foldchange
−3
−4
Lost Loops
Retained Loops

Fig. S4

a

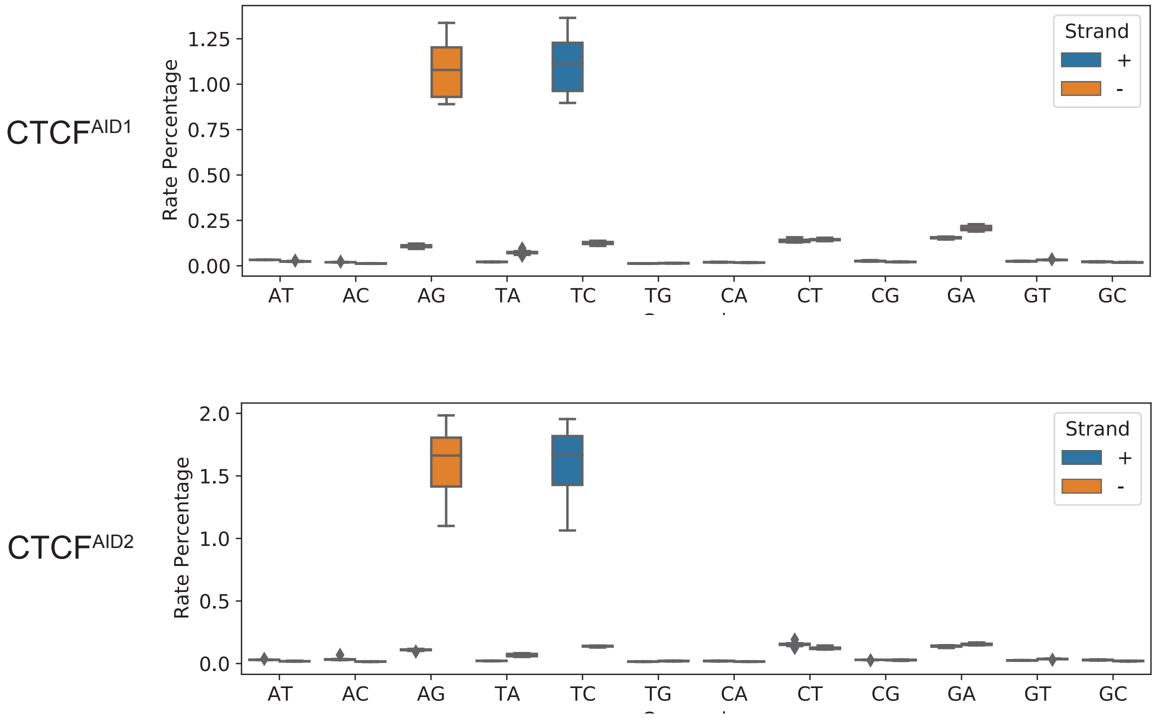
CTCF^AID1
Rate Percentage
Strand
+
-
AT AC AG TA TC TG CA CT CG GA GT GC
CTCF^AID2
Rate Percentage
Strand
+
-
AT AC AG TA TC TG CA CT CG GA GT GC

b

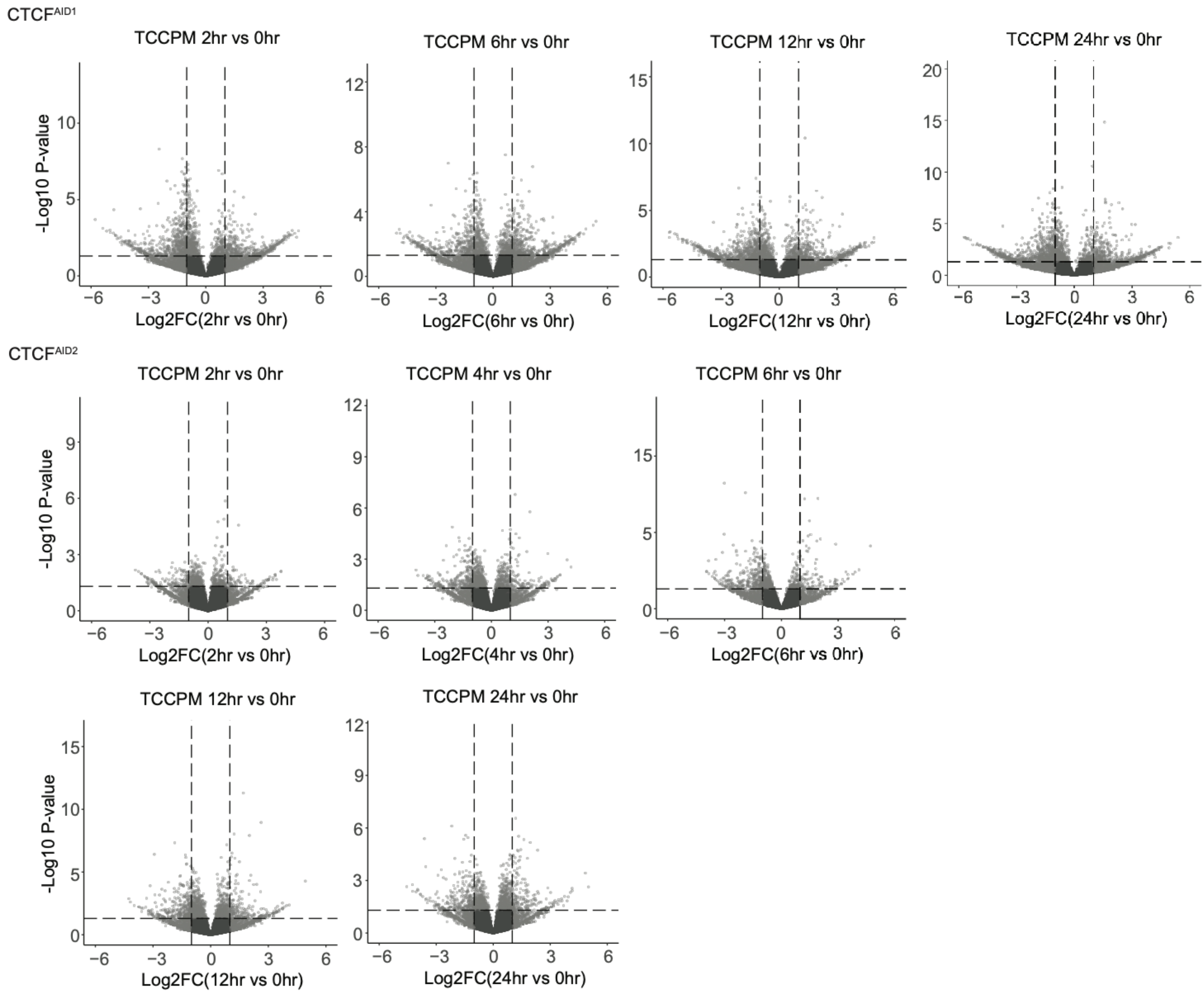
CTCF^AID1
TCCPM 2hr vs 0hr
TCCPM 6hr vs 0hr
TCCPM 12hr vs 0hr
TCCPM 24hr vs 0hr
-Log10 P-value
Log2FC(2hr vs 0hr)
Log2FC(6hr vs 0hr)
Log2FC(12hr vs 0hr)
Log2FC(24hr vs 0hr)
CTCF^AID2
TCCPM 2hr vs 0hr
TCCPM 4hr vs 0hr
TCCPM 6hr vs 0hr
Log2FC(2hr vs 0hr)
Log2FC(4hr vs 0hr)
Log2FC(6hr vs 0hr)
TCCPM 12hr vs 0hr
TCCPM 24hr vs 0hr
Log2FC(12hr vs 0hr)
Log2FC(24hr vs 0hr)

Fig. S5

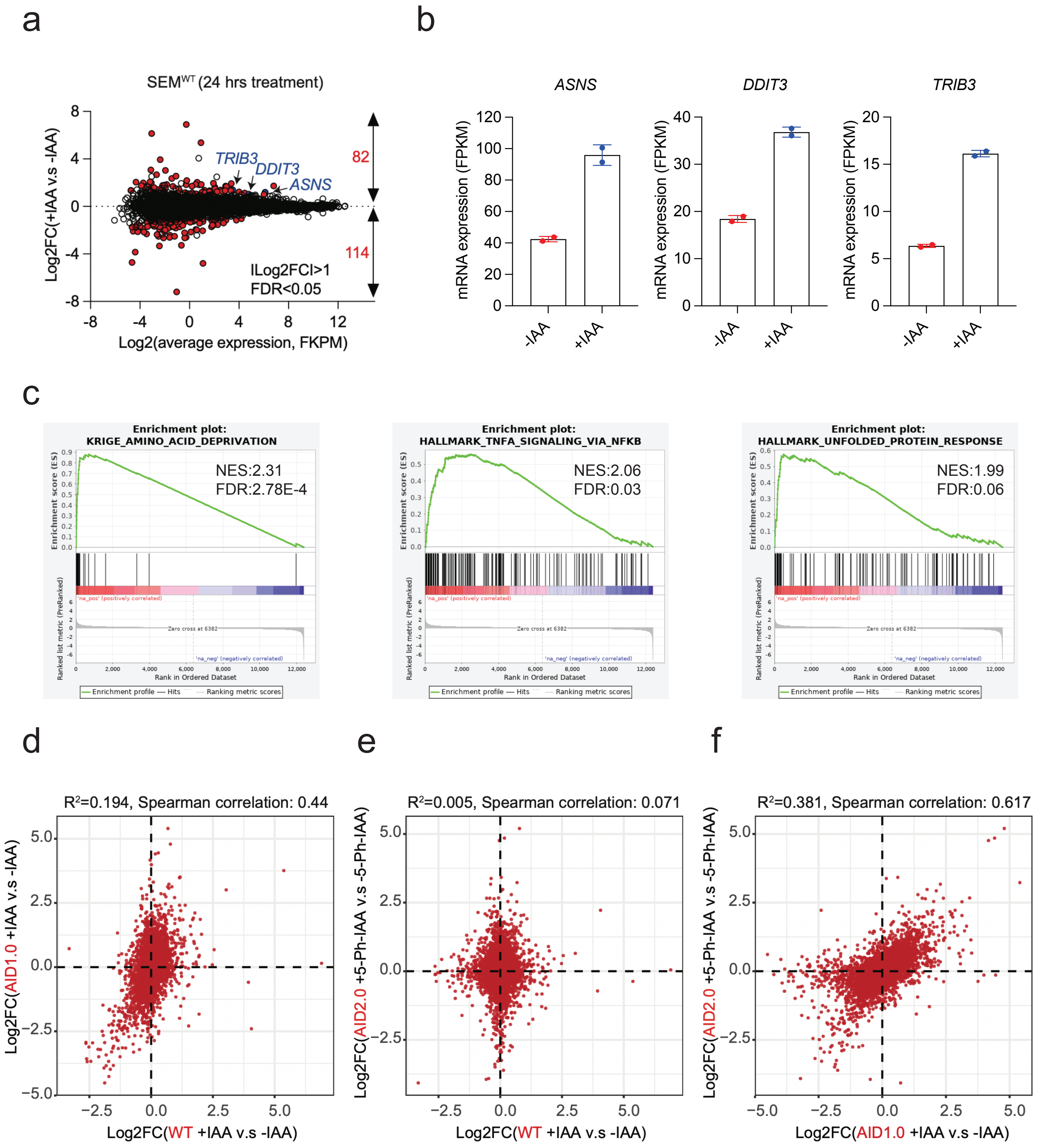
a
SEM^{WT} (24 hrs treatment)
Log2FC(+IAA v.s -IAA)
Log2(average expression, FKPM)
TRIB3
DDIT3
ASNS
82
114
ILog2FCI>1
FDR<0.05
b
ASNS
DDIT3
TRIB3
mRNA expression (FPKM)
-IAA
+IAA
c
Enrichment plot: KRIGE_AMINO_ACID_DEPRIVATION
NES:2.31
FDR:2.78E-4
Enrichment plot: HALLMARK_TNFA_SIGNALING_VIA_NFKB
NES:2.06
FDR:0.03
Enrichment plot: HALLMARK_UNFOLDED_PROTEIN_RESPONSE
NES:1.99
FDR:0.06
Enrichment score (ES)
Ranked list metric (PreRanked)
Rank in Ordered Dataset
Zero cross at 6382
'na_pos' (positively correlated)
'na_neg' (negatively correlated)
Enrichment profile — Hits — Ranking metric scores
d
R²=0.194, Spearman correlation: 0.44
Log2FC(AID1.0 +IAA v.s -IAA)
Log2FC(WT +IAA v.s -IAA)
e
R²=0.005, Spearman correlation: 0.071
Log2FC(AID2.0 +5-Ph-IAA v.s -5-Ph-IAA)
Log2FC(WT +IAA v.s -IAA)
f
R²=0.381, Spearman correlation: 0.617
Log2FC(AID2.0 +5-Ph-IAA v.s -5-Ph-IAA)
Log2FC(AID1.0 +IAA v.s -IAA)

Fig. S6

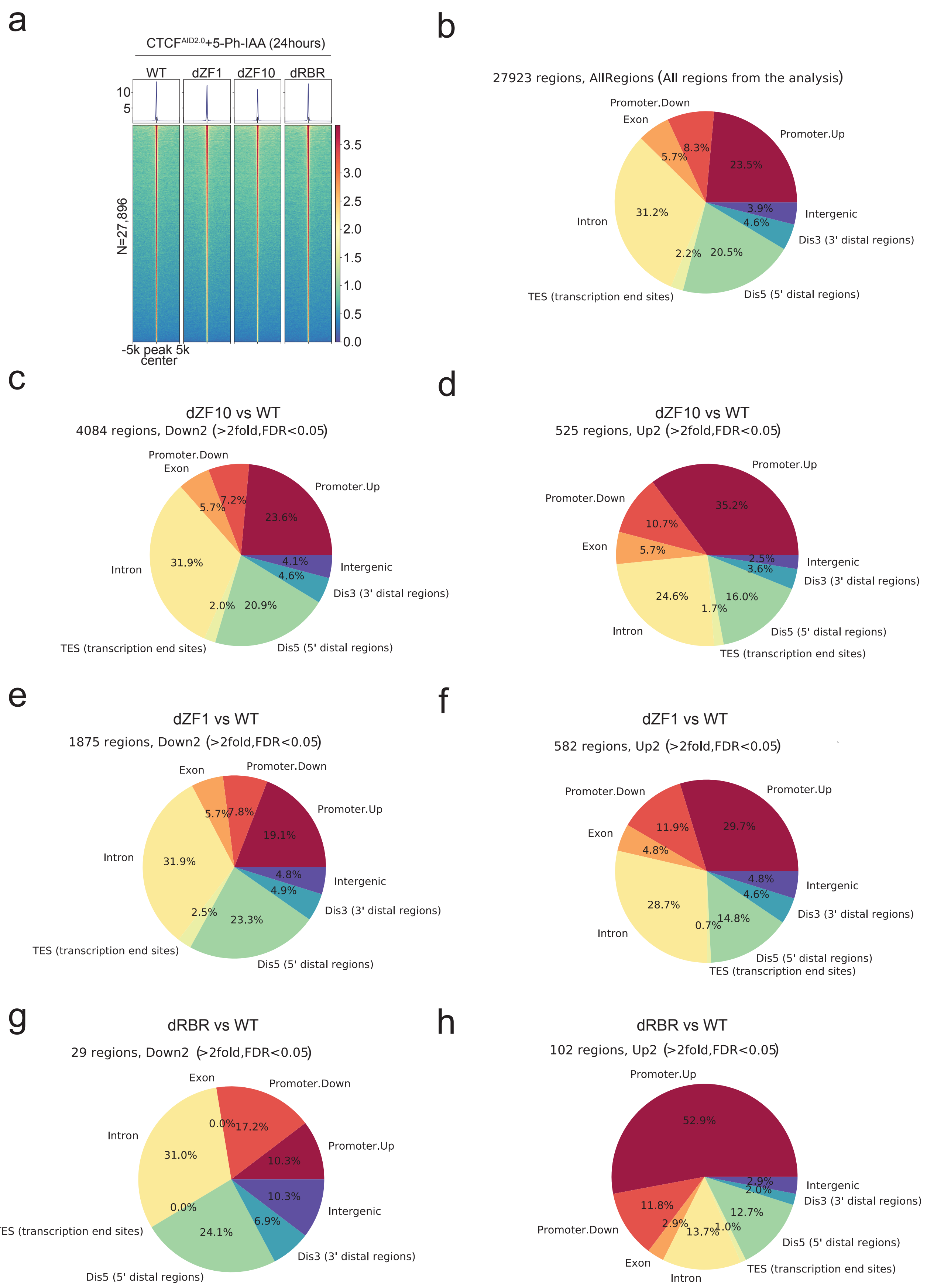

a
CTCF^AID2.0+5-Ph-IAA (24hours)
WT
dZF1
dZF10
dRBR
10
5
N=27,896
3.5
3.0
2.5
2.0
1.5
1.0
0.5
0.0
-5k peak 5k
center
b
27923 regions, AllRegions (All regions from the analysis)
Promoter.Down
Exon
Promoter.Up
8.3%
5.7%
23.5%
31.2%
Intron
3.9%
Intergenic
4.6%
Dis3 (3' distal regions)
2.2%
20.5%
TES (transcription end sites)
Dis5 (5' distal regions)
c
dZF10 vs WT
4084 regions, Down2 (>2fold,FDR<0.05)
Promoter.Down
Exon
7.2%
5.7%
Promoter.Up
23.6%
Intron
31.9%
4.1%
Intergenic
4.6%
Dis3 (3' distal regions)
2.0%
20.9%
TES (transcription end sites)
Dis5 (5' distal regions)
d
dZF10 vs WT
525 regions, Up2 (>2fold,FDR<0.05)
Promoter.Up
Promoter.Down
35.2%
10.7%
Exon
5.7%
2.5%
Intergenic
3.6%
Dis3 (3' distal regions)
24.6%
16.0%
1.7%
Intron
Dis5 (5' distal regions)
TES (transcription end sites)
e
dZF1 vs WT
1875 regions, Down2 (>2fold,FDR<0.05)
Exon
Promoter.Down
5.7%
7.8%
Promoter.Up
19.1%
Intron
31.9%
4.8%
Intergenic
4.9%
Dis3 (3' distal regions)
2.5%
23.3%
TES (transcription end sites)
Dis5 (5' distal regions)
f
dZF1 vs WT
582 regions, Up2 (>2fold,FDR<0.05)
Promoter.Down
Promoter.Up
11.9%
29.7%
Exon
4.8%
4.8%
Intergenic
4.6%
28.7%
Dis3 (3' distal regions)
0.7%
14.8%
Intron
Dis5 (5' distal regions)
TES (transcription end sites)
g
dRBR vs WT
29 regions, Down2 (>2fold,FDR<0.05)
Exon
Promoter.Down
0.0%
17.2%
Intron
31.0%
Promoter.Up
10.3%
10.3%
0.0%
Intergenic
6.9%
TES (transcription end sites)
24.1%
Dis3 (3' distal regions)
Dis5 (5' distal regions)
h
dRBR vs WT
102 regions, Up2 (>2fold,FDR<0.05)
Promoter.Up
52.9%
2.9%
Intergenic
2.0%
Dis3 (3' distal regions)
11.8%
12.7%
2.9%
13.7%
1.0%
Promoter.Down
Dis5 (5' distal regions)
Exon
Intron
TES (transcription end sites)

Fig. S7

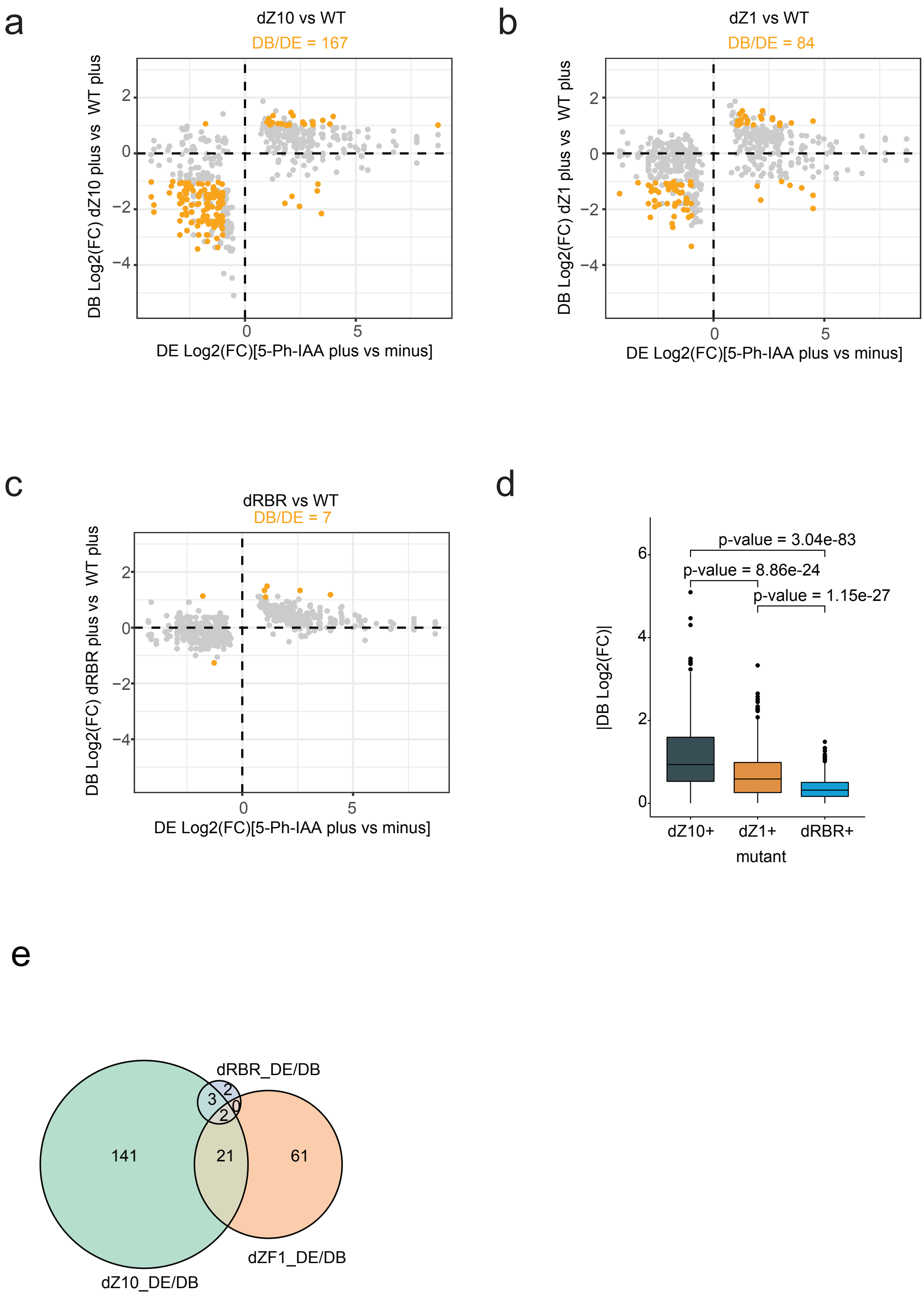
a
dZ10 vs WT
DB/DE = 167
DB Log2(FC) dZ10 plus vs WT plus
DE Log2(FC)[5-Ph-IAA plus vs minus]
b
dZ1 vs WT
DB/DE = 84
DB Log2(FC) dZ1 plus vs WT plus
DE Log2(FC)[5-Ph-IAA plus vs minus]
c
dRBR vs WT
DB/DE = 7
DB Log2(FC) dRBR plus vs WT plus
DE Log2(FC)[5-Ph-IAA plus vs minus]
d
p-value = 3.04e-83
p-value = 8.86e-24
p-value = 1.15e-27
|DB Log2(FC)|
dZ10+
dZ1+
dRBR+
mutant
e
dRBR_DE/DB
141
21
61
3
2
2
0
dZ10_DE/DB
dZF1_DE/DB
