# Supplementary material for: Auxin-inducible degron 2 system deciphers functions of CTCF domains in transcriptional regulation
Source: Genome Biol. 2023 Jan 26;24:14. doi: 10.1186/s13059-022-02843-3 (PMC9878928; doi:10.1186/s13059-022-02843-3)

Image ID: 0000671_01
Acquire Time: Dec 21, 2021 9:18:10 AM

Acquisition Information

#	Image ID	Acquire Time	Channels	Integration Times	Analysis	Image Name	Comment	Image Modifications
1	0000671_01	Dec 21, 2021 9:18:10 AM	Chemi	06:00	Western	0000671_01		

Image Display Values

Channel	Color	Minimum	Maximum	K
Chemi	Gray Scale (Black on White)	0.00000376	0.00132	0

2.0 time course

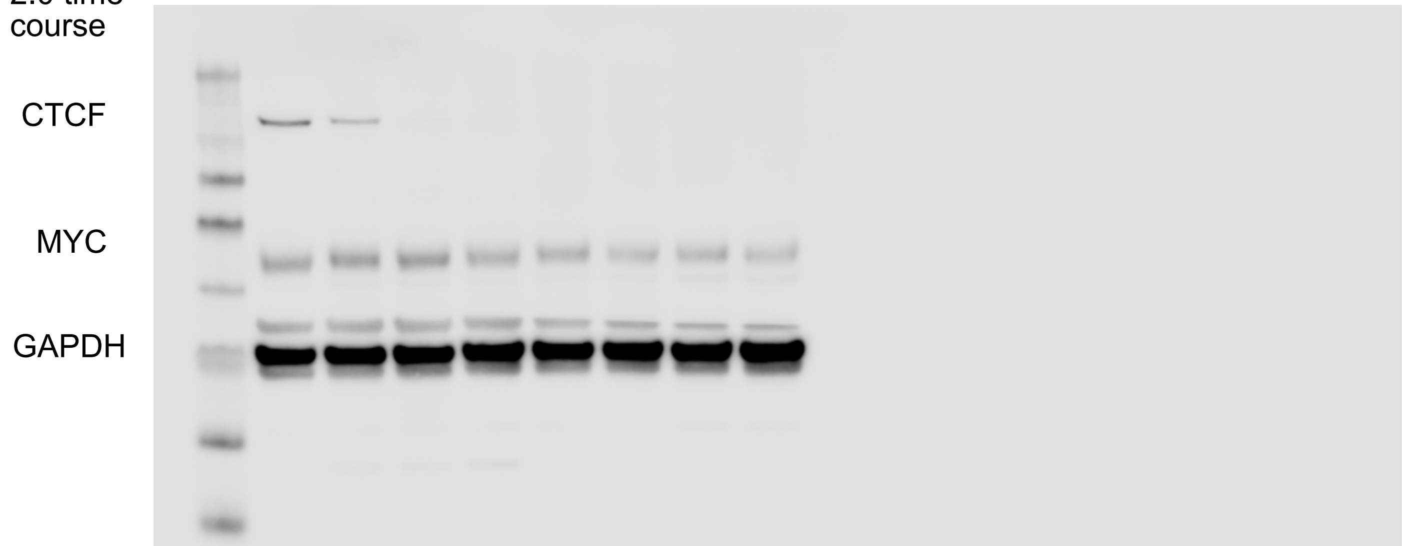

1.0 time course

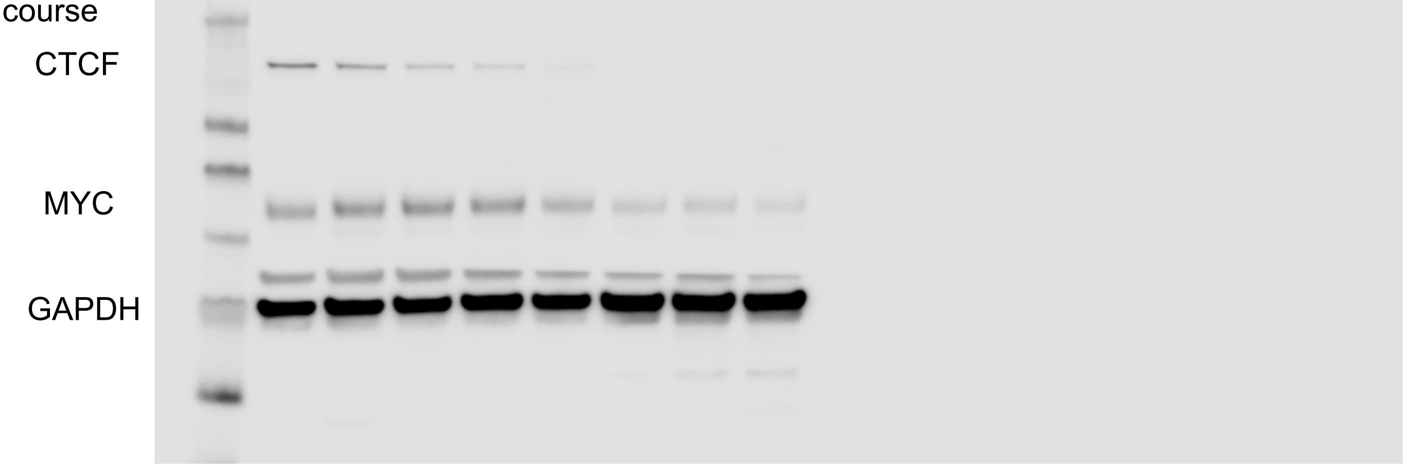

Image ID: 0000612_02
Acquire Time: Nov 10, 2021 11:05:16 AM

Acquisition Information

#	Image ID	Acquire Time	Channels	Integration Times	Analysis	Image Name	Comment
1	0000612_02	Nov 10, 2021 11:05:16 AM	Chemi	02:00	Manual	0000612_02	

Image Display Values

Channel	Color	Minimum	Maximum	K
Chemi	Gray Scale (Black on White)	0.00000668	0.000841	0.31

Fig 4B

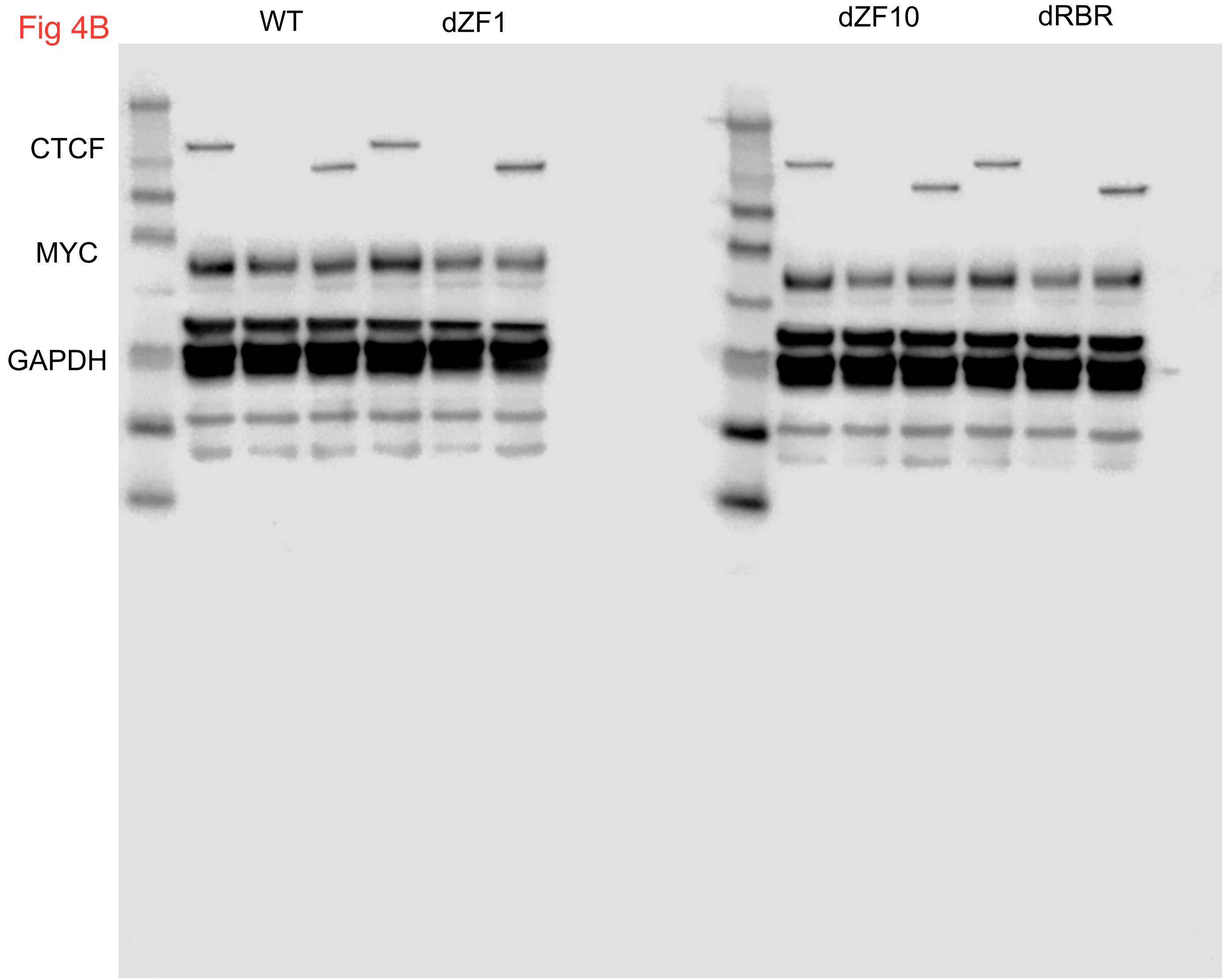

Image ID: 0000612_01
Acquire Time: Nov 10, 2021 11:05:16 AM

Acquisition Information

#	Image ID	Acquire Time	Channels	Integration Times	Analysis	Image Name	Comment	Image Modifications
1	0000612_01	Nov 10, 2021 11:05:16 AM	Chemi	02:00	Western	0000612_01		

Image Display Values

Channel	Color	Minimum	Maximum	K
Chemi	Gray Scale (Black on White)	0.00000167	0.00195	0

Fig 4B

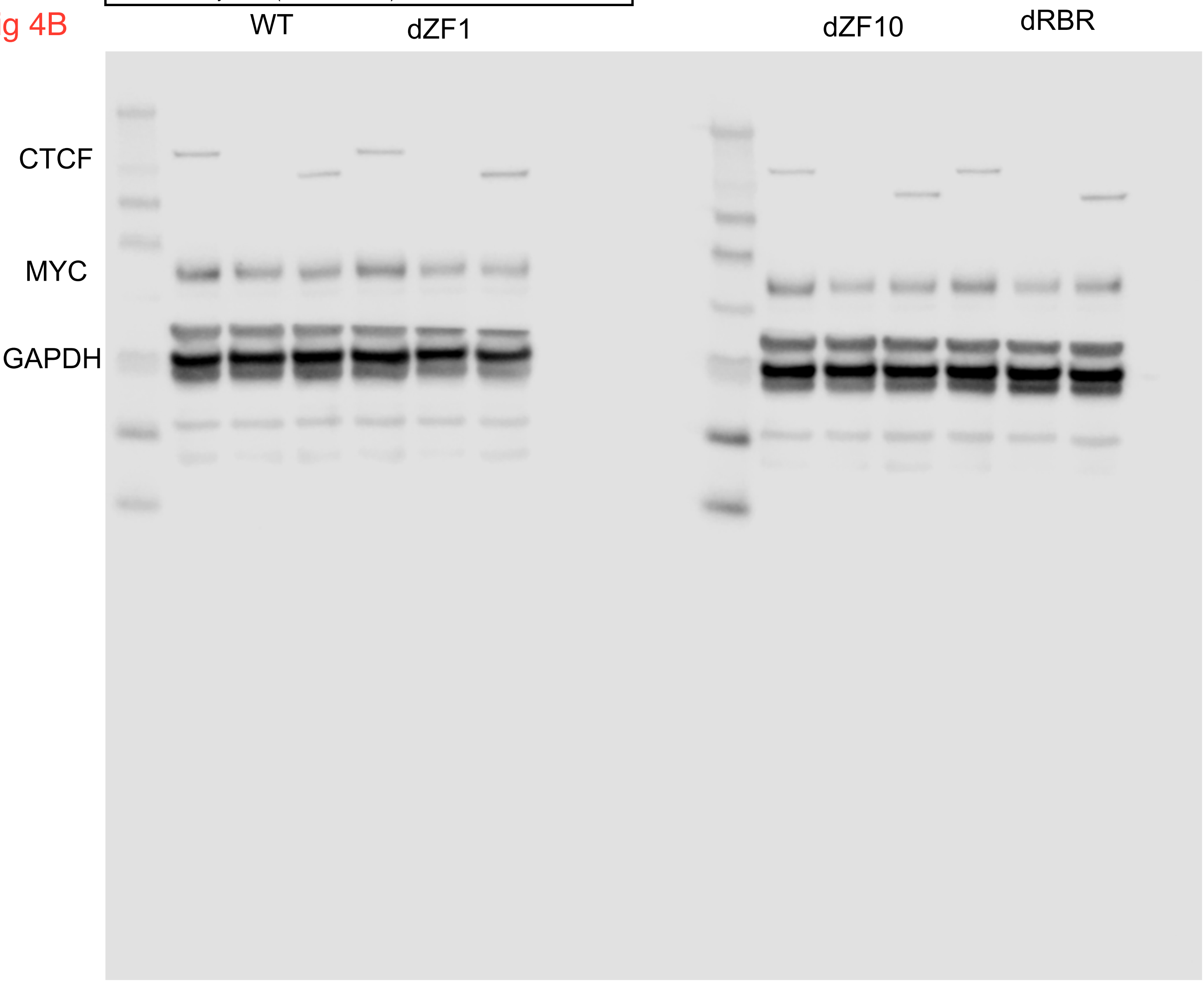

Image ID: 0000580_01
Acquire Time: Oct 27, 2021 9:26:35 AM

Acquisition Information

#	Image ID	Acquire Time	Channels	Integration Times	Analysis	Image Name	Comment	Image Modifications
1	0000580_01	Oct 27, 2021 9:26:35 AM	Chemi	02:05	Western	0000580_01		

Image Display Values

Channel	Color	Minimum	Maximum	K
Chemi	Gray Scale (Black on White)	0.00000131	0.000376	0

Fig S1a

CTCF

GFP
miniAID

not for this
paper

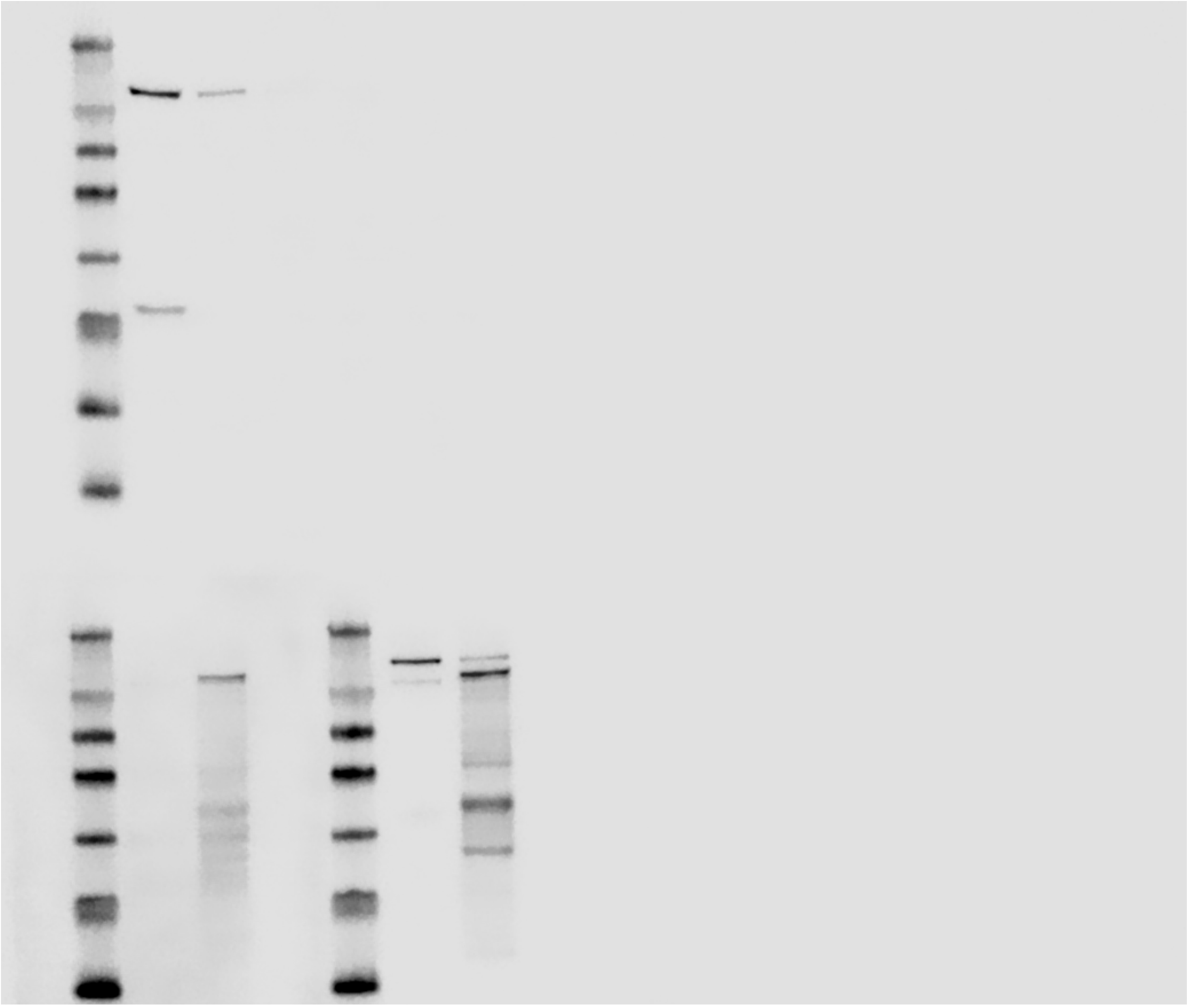

Image ID: 0000588_04
Acquire Time: Oct 28, 2021 11:34:21 AM

Acquisition Information

#	Image ID	Acquire Time	Channels	Integration Times	Analysis	Image Name	Comment
1	0000588_04	Oct 28, 2021 11:34:21 AM	Chemi	02:00	Manual	0000588_04	

Image Display Values

Channel	Color	Minimum	Maximum	K
Chemi	Gray Scale (Black on White)	0.0000000596	0.00147	0.21

Fig S1 a

GAPDH

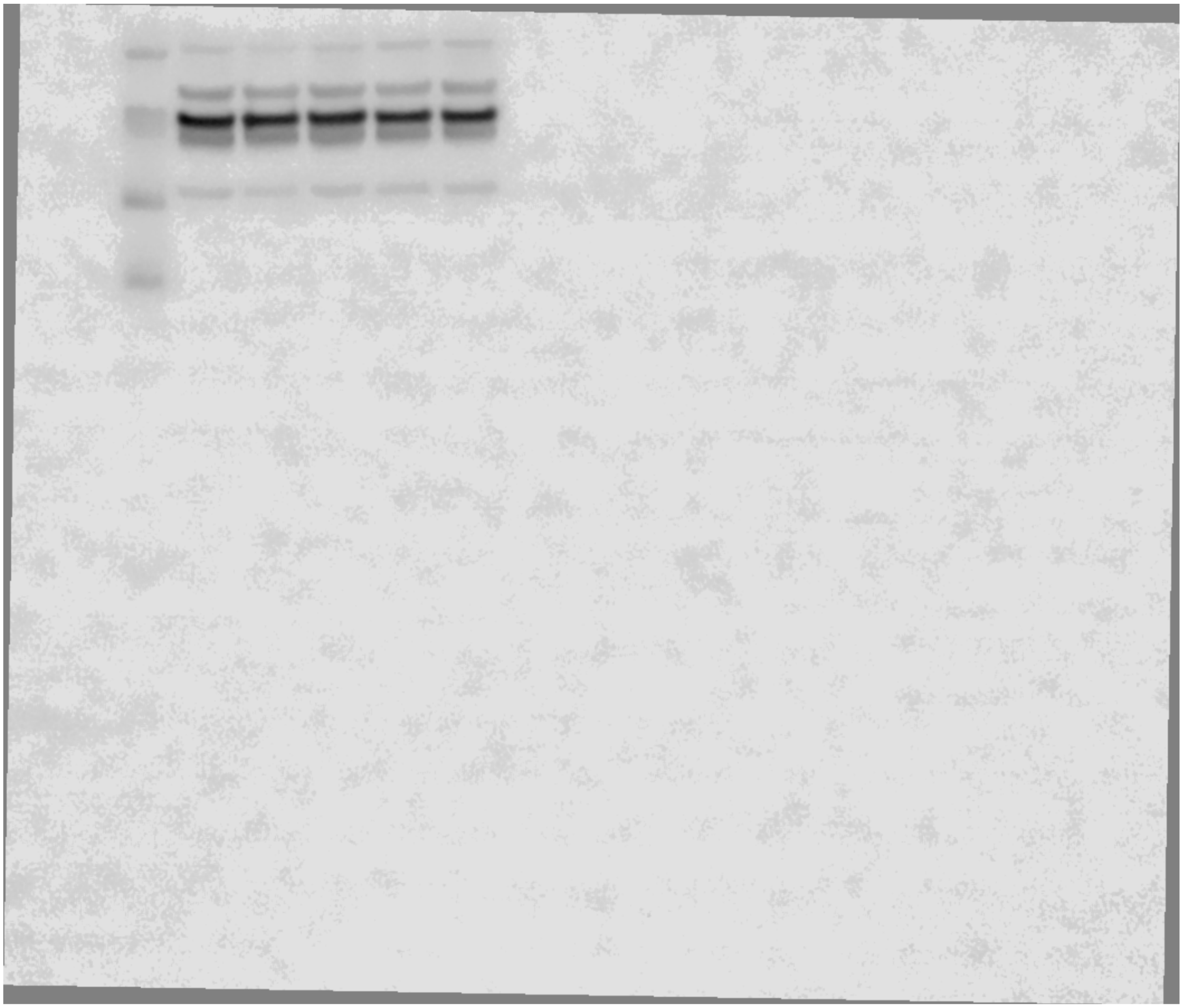

Acquisition Information (continued)

#	Image Modifications
1	Free Rotate 1 Image ID: 0000588_01; Duplicate Image ID: 0000588_02; Duplicate Image ID: 0000588_03

LI-COR®

Image ID: 0000644_01
Acquire Time: Nov 23, 2021 11:14:50 AM

Acquisition Information

#	Image ID	Acquire Time	Channels	Integration Times	Analysis	Image Name	Comment	Image Modifications
1	0000644_01	Nov 23, 2021 11:14:50 AM	Chemi	02:05	Western	0000644_01		

Image Display Values

Channel	Color	Minimum	Maximum	K
Chemi	Gray Scale (Black on White)	0.00000125	0.00142	0

Fig S1 b

GAPDH

not in this paper

not for this paper

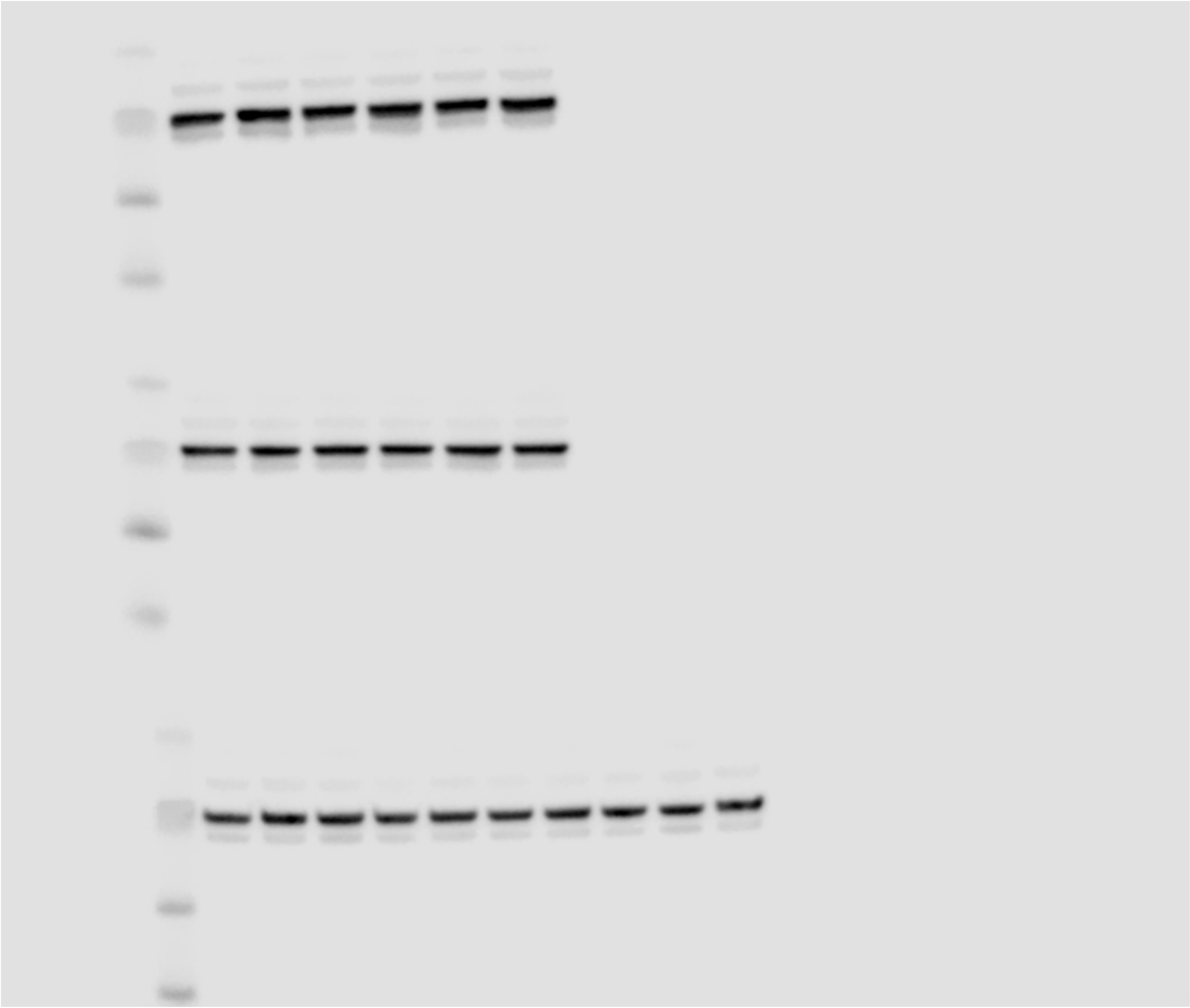

Acquisition Information

#	Image ID	Acquire Time	Channels	Integration Times	Analysis	Image Name	Comment
1	0001293_02	Aug 24, 2022 12:53:09 PM	Chemi	02:00	Manual	0001293_02	

Image Display Values

Channel	Color	Minimum	Maximum	K
Chemi	Gray Scale (Black on White)	0.000000954	0.00436	0

Fig S1 c

GAPDH

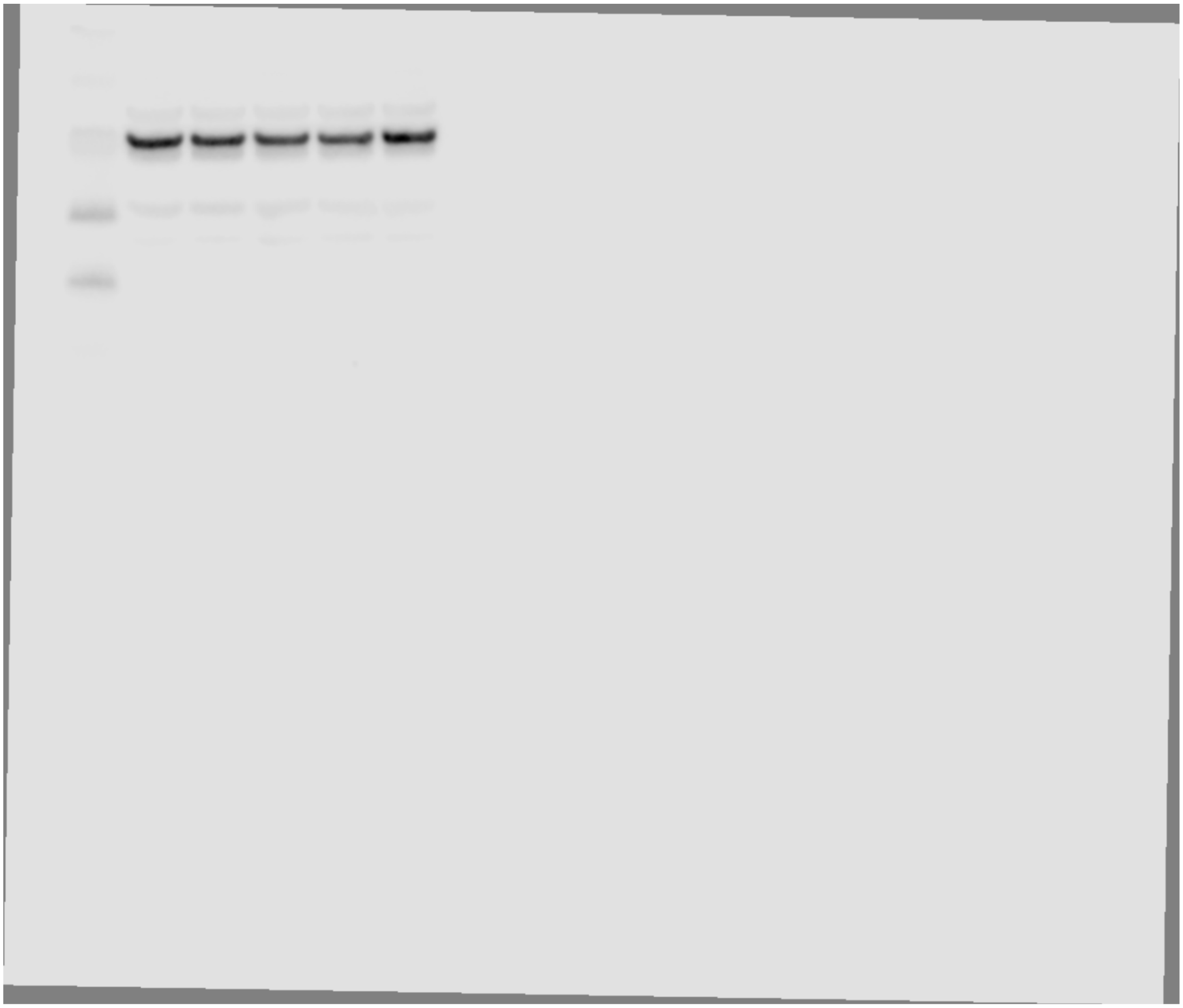

Acquisition Information (continued)

#	Image Modifications
1	Free Rotate 1 Image ID: 0001293_01

Acquisition Information

#	Image ID	Acquire Time	Channels	Integration Times	Analysis	Image Name	Comment
1	0000643_02	Nov 23, 2021 11:08:54 AM	Chemi	02:05	Manual	0000643_02	

Image Display Values

Channel	Color	Minimum	Maximum	K
Chemi	Gray Scale (Black on White)	0.0000000596	0.000534	0

Fig S1 b

CTCF

not in this paper

not in this paper

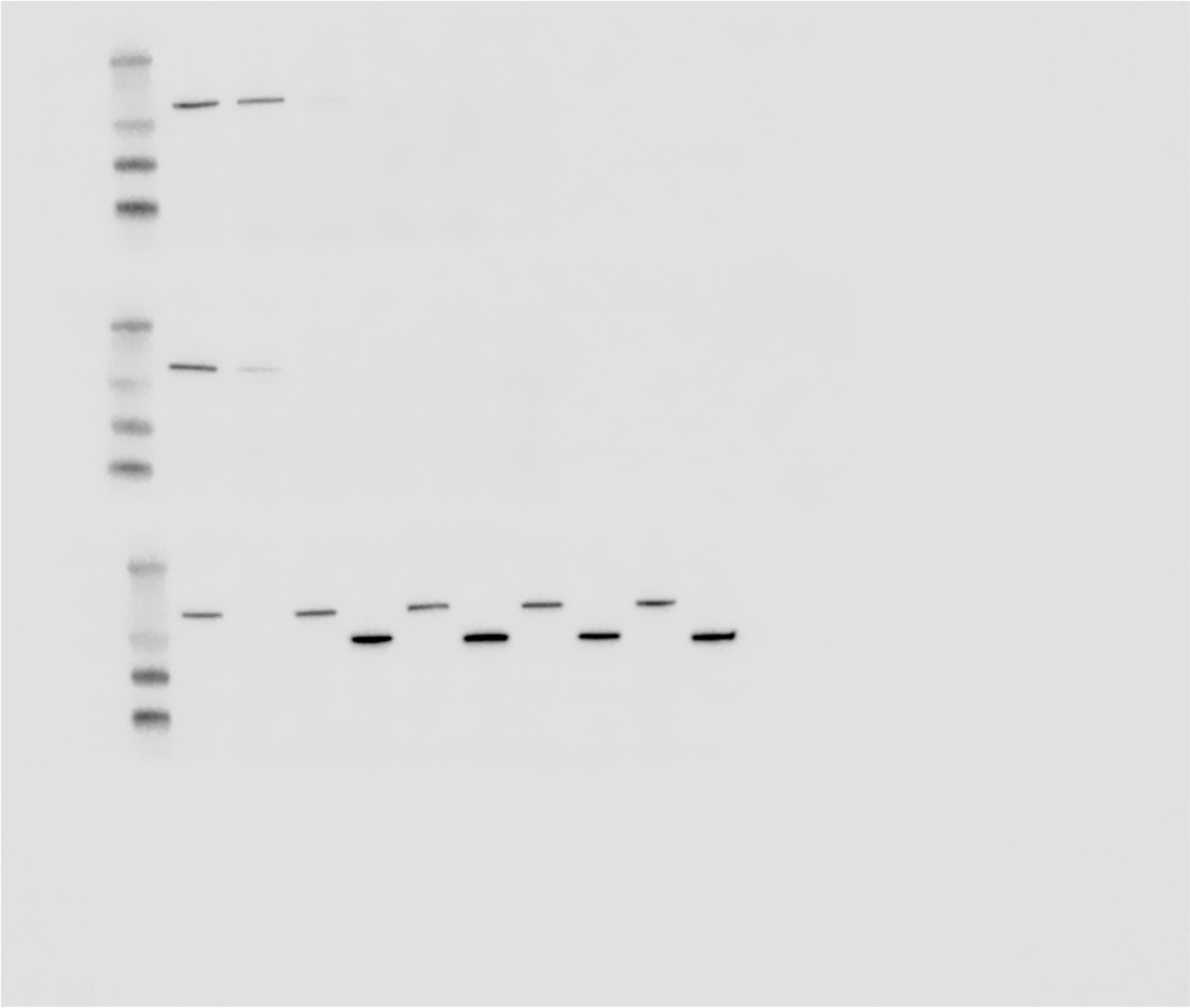

Acquisition Information (continued)

#	Image Modifications
1	Duplicate Image ID: 0000643_01

Image ID: 0001292_01
Acquire Time: Aug 24, 2022 11:20:57 AM

Acquisition Information

#	Image ID	Acquire Time	Channels	Integration Times	Analysis	Image Name	Comment	Image Modifications
1	0001292_01	Aug 24, 2022 11:20:57 AM	Chemi	02:00	Western	0001292_01		

Image Display Values

Channel	Color	Minimum	Maximum	K
Chemi	Gray Scale (Black on White)	0.00000131	0.00135	0

Fig S1 c

CTCF

not for this paper

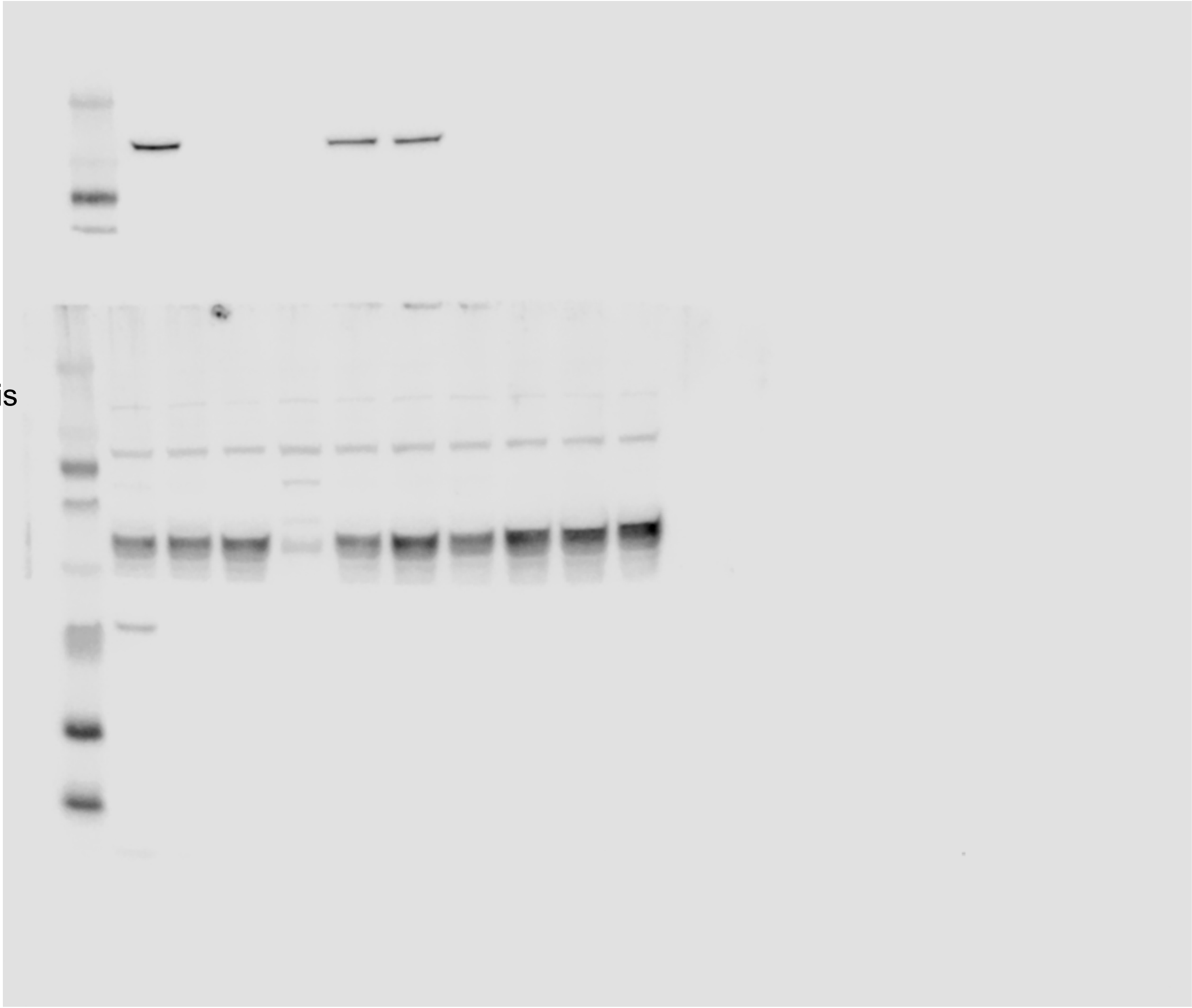

Image ID: 0001365_01
Acquire Time: Sep 14, 2022 1:02:42 PM

Acquisition Information

#	Image ID	Acquire Time	Channels	Integration Times	Analysis	Image Name	Comment	Image Modifications
1	0001365_01	Sep 14, 2022 1:02:42 PM	Chemi	02:00	Western	0001365_01		

Image Display Values

Channel	Color	Minimum	Maximum	K
Chemi	Gray Scale (Black on White)	0.0000733	0.00222	0

Fig S1 d

CTCF

OsTIR1

GAPDH

Fig 1C

CTCF

OsTIR1

GAPDH

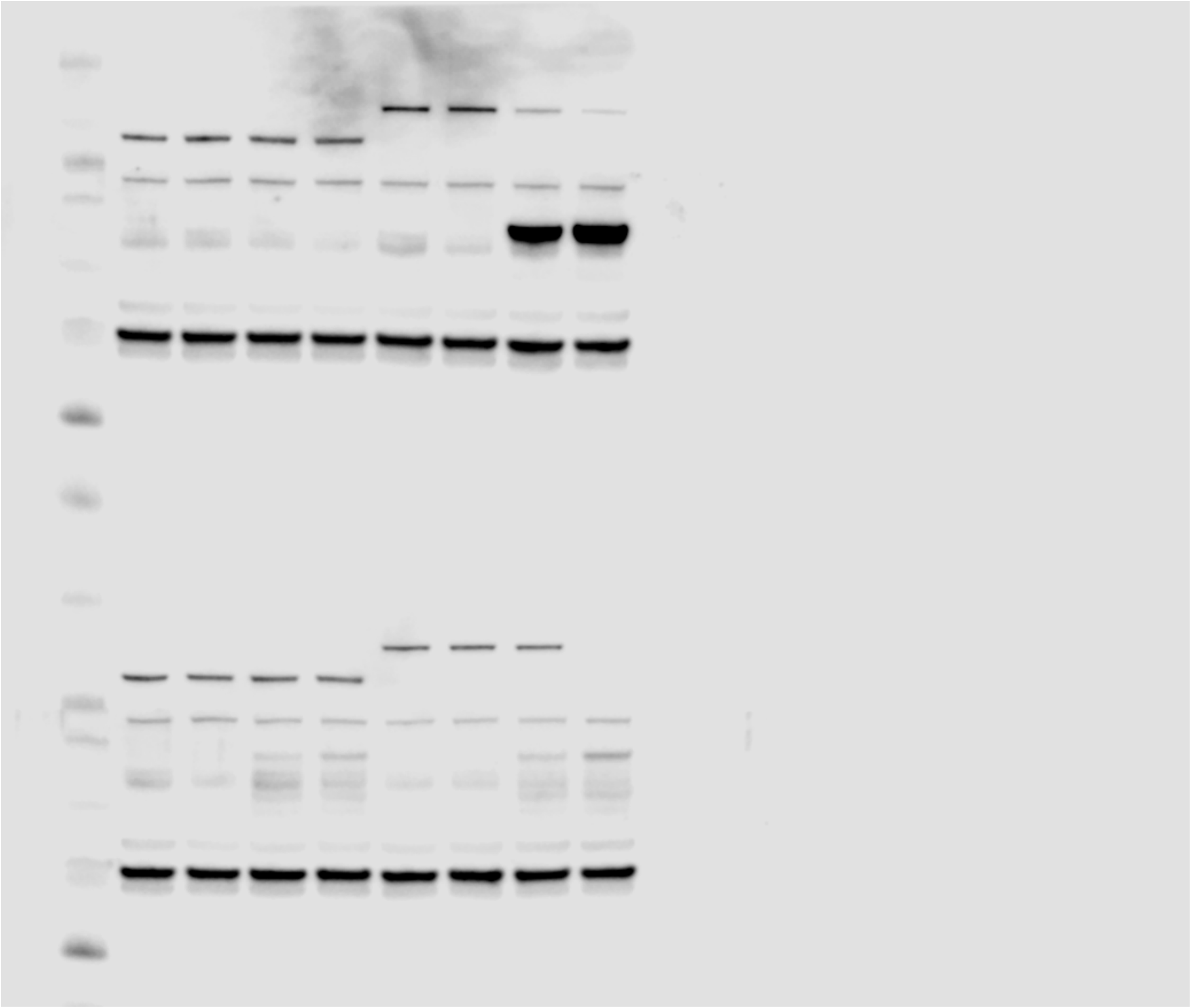

Supplement: Supplementary file 4 — Additional file 4. [file 13059_2022_2843_MOESM4_ESM.pdf]
