# Supplementary material for: Auxin-inducible degron 2 system deciphers functions of CTCF domains in transcriptional regulation
Source: Genome Biol. 2023 Jan 26;24:14. doi: 10.1186/s13059-022-02843-3 (PMC9878928; doi:10.1186/s13059-022-02843-3)
Supplement: Supplementary file 5 — Additional file 5. [file 13059_2022_2843_MOESM5_ESM.docx]

Review History

**First round of review**

**Reviewer 1**

**Were you able to assess all statistics in the manuscript, including the appropriateness of statistical tests used?**

No, I do not feel adequately qualified to assess the statistics.

**Comments to author:**

Unfortunately a number of serious conceptual and technical issues in this manuscript prevent me from recommending its publication.

Below I explain my reservations.

1. Lack of proper characterization of degron systems.

a) unclear if tagged cells are leaky

While authors do an intricate job comparing AID1 and AID2, not including a comparison to Tir1-expressing UNTAGGED CTCF in any of their assay prevents them from measuring to what extent AID1/2 tagging (BEFORE depletion) may destabilize CTCF - a well known problem reported in all papers using degron-tagging of CTCF (AID1 or dTAG).

b) unclear is depletion is total

-Figure 1b is missing untagged cells - without this it is impossible to conclude about how complete the depletion is. Would the Western-blot pick up 10-20% leftover?

-The fact that long-term CTCF AID2 depletion (4 days) is not lethal I surprising given the fact that CTCF is essential. Is it possible this cell line expresses CTCF isoforms that do not include the AID tag and escape detection by the Western blot assay? This has been observed before - e.g. Valton et al. Biorxiv 2021.

c) unclear to what extent proliferation / gene expression changes are contributed to by Tir1 as opposed to degrading CTCF

Authors did not include Tir1-expression CTCF-untagged cells in any of their experiments. Therefore it is very difficult to estimate to what extent AID1/2 tagging in itself (before inducing depletion) may disrupt CTCF expression or functions. Authors also do not provide data regarding auxin/ 5-Ph-IAA treatment in TIr1 expressing cells. Therefore, it is not possible to conclude that proliferation or cell cycle defects are solely due to CTCF depletion and not ectopic degradation of non-AID targets upon induction.

2. Poor reporting of gene expression data

- In figure 2 how does authors quantify total RNA changes? It is not possible to understand Figure 2C with the information provided. please explain in the legend what "steady-state" / "total" means. mRNA-seq separately in AID1 and AID2? After depleting for how long?

- Authors do not report the fold change of dysregulated genes

- In figure 5a what does "RNA-seq"mean? SLAM-seq nascent transcripts? Other data not described in the methods section?

- It is very unusual to detect more genes dysregulated with TOTAL RNA vs nascent (fig 2) - without more information on where the numbers behind "TOTAL" comes from it is impossible to provide further advice.

3. Biological insight into CTCF biology largely redundant with previous studies

- Previous work already demonstrated that the entire N and C termini of CTCF appear dispensable for cell viability in culture (e.g. Nishana et al. Genome Biology 2019, Nora et al. 2020 Nature Comms), albeit serving biological functions (e.g. RNA-binding, cohesin binding, trans-activation etc…). Same for ZF1 and ZF10 (Saldana-Meyer et al Mol cell 2019). Saldana-Meyer et al Mol cell 2019 and Nakahashi et al Cell Reports 2014 also already showed that CTCF binding is disrupted in these mutants. Hansen et al. 2019 Mol Cell and Nora et al. 2020 Nature Comms already showed that deleting the RBRi also disrupts CTCF binding.

- Nakahashi et al Cell Reports 2014 already reported that disruption of ZF1 or ZF10 disrupts CTCF binding depending on the underlying DNA motif. The analysis presented here in figure 6 does move beyond, by reporting specific nucleotide dependency in different classes of sites. However, experiment presented do not address whether the functional importance of ZF1/ZF10 detected in the genetic screen is actually mediated by DNA binding, as opposed to other processes (such as the ability of these domains to mediate RNA binding - Saldana-Meyer et al Mol Cell 2019).

Additional technical limitations of the study

- ChIP-seq experiments did not have spike-in controls. Exact quantification of binding strength is therefore not possible and conclusions remain qualitative when comparing the various mutants. While this is OK to state that CTCF binding is reduced after depletion it prevents stating how reduced it is and the effect in the various mutants is likely under/over estimated.

- Figure 1m. Doing Hi-ChIP for CTCF after CTCF degradation is uninformative. Since the protein is degraded it is of course expected that the CTCF HiChIP signal will be dramatically reduced. This approach therefore does not allow concluding anything about chromatin looping. I do not think this experiment is meaningful in any way. Hi-C of Hi-ChIP for a factor that is demonstrated to remain stable upon CTCF depletion could be used to address changes in genome folding but certainly not CTCF Hi-ChIP.

I am sorry I cannot be more positive. I encourage authors to consider solidifying the characterization of the AID2 system with controls detailed above, clarify the presentation and exploration of the transcription data and, if possible, use their data to design additional experiments/analyses that are less confirmatory to yield more novel insight on CTCF biology.

**Reviewer 2**

**Were you able to assess all statistics in the manuscript, including the appropriateness of statistical tests used?**

Yes, and I have assessed the statistics in my report.

**Comments to author:**

In this manuscript, Hyle et al. introduced an improved auxin-inducible degron (AID) technology, AID2, to interrogate CTCF protein level and its genome-wide binding. They found that the AID2 system was better than the previous AID1 system for rapid degrading CTCF with reduced cellular toxicity. They used CRISPR screen combined with an induced exdogenous CTCF mutant model, to study the function of zinc-finger (ZF) domains ZF1 and ZF10 in CTCF DNA binding and transcriptional regulation in the B-ALL SEM cell line system. The authors showed convincing evidence that AID2 can be a useful assay to study CTCF protein function, and provide insights into the functional relevance of ZF domains to CTCF molecular and regulatory functions, as demonstrating a broad interests in the field. The manuscript is overall well written, with data presented clearly. I have some specific comments as follows:

Major points:

1. The authors stated that 27,727 out of the 46,556 CTCF peaks "were completely lost following 24 hours treatment" (lines 128-129). This is a very important result that should be better presented in the figure. Fig 1l shows 46,620 peaks. Is it true that only 64 peaks in the 46,620 "combined peaks" are not "reproducible peaks"? How are "combined peaks" defined and what is the difference? Also, it would be better if the 27,727 lost peaks can be clearly labeled in Fig. 1l. How are the peaks ranked in Fig 1l? A more detailed description should be included. More importantly, what are the differences between the 27,727 lost peaks and the remaining insensitive peaks? Do they tend to be stronger peaks or weaker peaks in untreated cells? Do they show different distribution in the genome? i.e., are they more enriched in TAD boundaries, promoters, enhancers, or evenly distributed across all peaks?

2. In the HiChIP analysis shown in Fig. 1m, what do the APA numbers (2.684 & 1.338) mean? The detailed procedure of the aggregate peak analysis needs to be included in the manuscript. How many loop regions were included in the analysis for each of the two plots? How were normalizations and quantitative comparisons were done? Also, for the lost and retained loops, are they primarily enhancer-promoter (E-P) loops, E-E loops, or P-P loops? How many loop anchors colocalize with CTCF binding sites? Are the lost chromatin loops colocalized and correlated with lost CTCF binding sites? Also, it is unclear how the analysis was done in Extended Fig. S1c-d, especially the Hi-C data. Why the Hi-C and HiChIP (untreated) have so few overlapped loops? These analyses are essential to demonstrate the effect of CTCF degradation to chromatin conformation changes.

3. Detailed PCA procedures that were used to generate Fig. 1k (ChIP-seq) and Fig. 2b (SLAM-seq) should be described in the Methods section. It is unclear how ChIP-seq peak signals were calculated and whether/how normalization was done. For Fig. 2b (AID2), it is unclear why PC1 (with only 16.45% variance explained) can indicate "gradual and constant shift in transcript variability over time" (line 153).

4. In lines 171-173, the authors claim that "some of the transcription dysregulation observed in the MYC target pathways using the AID1 system may be attributed to off-target effects of combined high concentration auxin treatment and CTCF depletion." How was this conclusion made based on the pathway enrichment analysis? This statement seems unfounded and needs more evidence to support and more elaboration.

5. The authors use DESeq2, and RNA-seq differential analysis tool, to identify differentially represented sgRNAs. However, MAGeCK (PMID: 25476604) is more appropriate for analyzing CRISPR screen data. Some justification is needed to support the authors' choice.

6. In lines 240-241, the authors state that "CTCF binding can regulate transcription either proximally to the transcription start site (TSS) or distally within the target's TAD boundaries." Additionally, in fact, CTCF can also regulate transcription by binding at distal enhancer regions within a TAD but distinct from TAD boundary regions (e.g., Schuijers et al 2018 PMID: 29641996, Fang et al 2020 PMID: 32933554). I would suggest that the authors revise the statement and cite these references.

7. In lines 241-243, the authors state "The 50kb region upstream and downstream of the TSS and transcription end site (TES), respectively, were considered when assigning CTCF binding to DE genes." This contradicts to the description in the Methods section (lines 606-608), where they say "TSS+/-50kb". This should be clarified.

8. For the statistical analysis shown in Fig. 5b and Extended Fig. S4, p-values should be presented in addition to F-statistics to properly demonstrate the statistical significance. For the dRBR control (Fig. 5b right and Fig. S4c), F-statistic should also be presented even if the p-value does not meet the significance threshold. Also, R^2 is not an appropriate metric to use, as the data points were preselected as differential genes and peaks, not reflecting the whole sample.

Minor points:

9. Lines 225 and 601: Citations of PMIDs need to be included in the references.

10. In addition to a more detailed description of computational analysis methods, it would be helpful to include the complete code and scripts in a code repository, to ensure reproducibility of the results.

**Authors Response**

**Point-by-point responses to the reviewers’ comments:**

Reviewer #1:

Unfortunately a number of serious conceptual and technical issues in this manuscript prevent me from recommending its publication.

Below I explain my reservations.

Major Comments:

1. Lack of proper characterization of degron systems.

a) unclear if tagged cells are leaky

While authors do an intricate job comparing AID1 and AID2, not including a comparison to Tir1-expressing UNTAGGED CTCF in any of their assay prevents them from measuring to what extent AID1/2 tagging (BEFORE depletion) may destabilize CTCF - a well-known problem reported in all papers using degron-tagging of CTCF (AID1 or dTAG).

*Response: Thank you for this suggestion. We agree with the reviewer that a more thorough examination of CTCF expression and function between cell lines expressing tagged and untagged CTCF and in OsTIR1 expressing cells, with and without CTCF tagging, would be informative. To address protein expression in AID1 settings, we treated SEMWT and CTCFAID1 cells (carrying a miniAID tagged CTCF and a doxycycline-inducible WT OsTIR1) with either individual or a combination of 500 M IAA and 1 g/ml doxycycline for 24 hours (see next page: Additional File 1: Fig. S1d). As expected, endogenous CTCF expression in SEMWT cells was unaffected by all treatment conditions. Tagged CTCF expression in CTCFAID1 cells was comparable to SEMWT levels without treatment and with IAA alone, which showed tagging CTCF did not destabilize its expression. However, induction of OsTIR1 by doxycycline did cause leaky degradation of CTCF, a known limitation of the AID system using WT OsTIR1 [1-3].*

*To examine CTCF expression levels in the AID2 system, we compared the expression of CTCF between SEMWT, SEMOsTIR1(F74G) [SEMWT cells expressing OsTIR1(F74G)], CTCFAID1 and CTCFAID2 cells with and without 1 M 5-Ph-IAA treatment for 24 hours (see below: New Fig. 1c). CTCF expression was consistent between SEMWT, SEMOsTIR1(F74G) and CTCFAID1 regardless of OsTIR1(F74G) expression, tagging of CTCF, or 5-Ph-IAA. In addition, no leaky degradation of CTCF was observed in untreated CTCFAID2 cells, which have tagged CTCF and OsTIR1(F74G). CTCF degradation was only observed in CTCFAID2 cells after the addition of 5-Ph-IAA. These data support the AID2 degradation system overcomes the limitation of leaky degradation observed in the previous AID1 system. We also provide data to confirm the 5-Ph-IAA-induced degradation of CTCFAID through the proteasome can be completely rescued with MG132 treatment (see next page: Additional File 1: Fig. S1c).*

*New Fig. 1c. Immunoblot analysis from SEMWT, SEMOsTIR1(F74G), CTCFAID1 and CTCFAID2 cells following 24 hours of DMSO or 1 M 5-Ph-IAA treatment. The lower CTCF band represents untagged endogenous CTCF (CTCF-WT). The higher CTCF band represents CTCFAIDmClover3 (CTCF-AID). GAPDH was used as a loading control.*

*To determine what effect the various treatment conditions would have on cell proliferation, we set up a four-day growth assay in AID1 or AID2 settings. Under AID1 treatment conditions, SEMWT cells showed a marked reduction in cell proliferation with 500 M IAA alone, demonstrating high auxin treatment was not well tolerated by SEM cells (see below: Additional File 1: Fig. S1e). Cell proliferation was further reduced in AID1 cells when high auxin treatment was combined with CTCF loss (see below: Additional File 1: Fig. S1f). In contrast, SEM cells grew similarly regardless of 5-Ph-IAA or OsTIR1(F74G) expression (see below: Additional File 1: Fig. S1g). Even in CTCFAID2 cells with 5-Ph-IAA treatment, no significant growth defect was observed. These observations support high auxin treatment can be toxic to SEM cells, and the ability to use less auxin with OsTIR1(F74G) overcomes this additional limitation of the AID1 system. The immunoblotting and growth assays presented here will be added to Additional File 1: Fig. S1 and discussed in the revised manuscript.*

*Additional File 1: Fig. S1. a) Immunoblot analysis from CTCFAID2 cells following 0-24 hours 10 M 5-Ph-IAA treatment; CTCFAIDmClover3 was detected by a CTCF antibody and EGFPAID2 was detected by a miniAID antibody. b) Immunoblot analysis of CTCFAID2 cells treated with a 1 M 5-Ph-IAA titration for 6 hours. c) Immunoblot analysis of CTCFAID2 cells co-treated with 1 M 5-Ph-IAA and proteasome inhibitor MG132 for 5 hours. d) Immunoblot analysis of SEMWT and CTCFAID1 cells treated with 500 M IAA, 1g/ml doxycycline, or a combination of both for 24 hours. The lower CTCF band represents untagged endogenous CTCF. The higher CTCF band represents the miniAID tagged endogenous CTCF (CTCFAIDmClover3). e) Growth assay of SEMWT cells treated with DMSO, 500 M IAA, 1g/ml doxycycline, or a combination of both over 4 days. Samples were set up in triplicate and cell* counts for *each replicate were collected daily. Bars represent cell numbers (x millions). f) Growth assay of CTCFAID1 cells treated with DMSO, 500 M IAA, 1g/ml doxycycline, or a combination of both over 4 days. Samples were set up in triplicate and cell counts for each replicate were collected daily. Bars represent cell numbers (x millions). g) Growth assay of SEMWT, SEMOsTIR1(F74G) and CTCFAID2 cells treated with DMSO or 1M 5-Ph-IAA over 4 days. Samples were set up in triplicate and cell counts for each replicate were collected daily. Bars represent cell numbers (x millions).*

b) unclear is depletion is total

-Figure 1b is missing untagged cells - without this it is impossible to conclude about how complete the depletion is. Would the Western-blot pick up 10-20% leftover?

-The fact that long-term CTCF AID2 depletion (4 days) is not lethal I surprising given the fact that CTCF is essential. Is it possible this cell line expresses CTCF isoforms that do not include the AID tag and escape detection by the Western blot assay? This has been observed before - e.g. Valton et al. Biorxiv 2021.

*Response: Thank you for bringing this important point to our attention. Fig. 1c has been updated to include untagged cells as requested (see above).*

*Although no severe lethality was observed after the degradation of CTCF for 4 days, we have the following explanation for the phenotype discrepancy as CTCF is essential based on genetic knockout results. First, our previous dropout transcription factor (TF) CRISPR screen suggested CTCF is a survival dependency gene but showed a less strong effect when compared with PAX5, DOT1L, YY1, etc. in SEM cells [4]. Therefore, the context-dependent role may vary when compared with other cells. Second, we and others have observed that after CTCF depletion by either AID or RNAi, a very small fraction of CTCF remains bound to the chromatin at “persistent” CTCF binding sites [5-8], which demonstrate reduced but maintained binding. In CTCF ChIP seq of CTCFAID2 cells, 43.4% of CTCF-binding sites (CBSs) exhibit complete loss of CTCF binding (see below: Additional File 1: Fig. S2a-S2b). The remaining CBSs retain persistent but significantly reduced binding, as observed in the heatmap in Fig. 1l and Additional File 1: Fig. S2a-b. In addition, CTCF looping was retained in ~3% of CTCF-dependent loops identified by HiChIP (Additional File 1: Fig. S3b). It is possible that this minimal representation of CTCF is sufficient to maintain survival. The observations of persistent CTCF binding following depletion were referenced in the manuscript, but we will add that while CTCF appears completely degraded by immunoblot (unless greatly overexposed; see Response Figure R1a at page 6 below), the cells maintain a fraction of CTCF with reduced, persistent binding at a subset of CBSs that may be sufficient to partially support survival. Based on this evidence, we will also carefully clarify descriptions of CTCF degradation in the text, so it will be understood the levels are undetectable by immunoblot, not 100% degraded.*

*Additional File 1: Fig. S2. a) CTCF binding enrichment heatmap centered at CTCF peaks showing the binding intensity of CTCF peaks before and after 5-Ph-IAA treatment. Two main groups are identified: i) CTCF peaks that significantly reduced in intensity but were not completely lost, and ii) CTCF peaks that were completely lost after 5-Ph-IAA treatment. An additional 67 peaks identified in AID2 treated cells are not shown in the heatmap due to low confidence. b) Meta plot showing the mean CTCF peak intensity in untreated cells of the lost and significantly reduced peaks. c) Stacked Bar-plot showing the genomic distribution of CTCF peaks from panel a. d) Genomic distribution of CTCF peaks from c) with a more stringent cutoff (FDR<0.05, |log2(FC)|>1). e) Boxplot plot showing the genomic distance of lost and significantly reduced CTCF peaks to nearby domain boundaries identified by HiChIP.*

*In a separate but related study to see how continuous CTCF depletion would affect cells, we carried out 1 M 5-Ph-IAA treatment in CTCFAID2 cells for a 21-day time course. Surprisingly, not only were the cells able to survive, but the tagged CTCF became resistant to auxin depletion (Response Figure R1a). However, when CTCF-WT was concurrently exogenously expressed, tagged CTCF remained sensitive to degradation. Indeed, even at a long exposure, no miniAID tagged CTCF was observed in CTCF WT expressing cells, which suggests tagged CTCF was fully sensitive to auxin degradation with exogenous CTCF rescue. Increasing the auxin treatment did not restore sensitivity to degradation (Response Figure R1b). Cells under continuous 5-Ph-IAA challenge for 19 days that had developed auxin-resistant CTCF were removed from 5-Ph-IAA for 48hrs or 6 days and challenged again with 5-Ph-IAA to see if CTCF would regain sensitivity to auxin. After both washout conditions, CTCF remained insensitive to auxin degradation (Response Figure R1c). However, an internal GFP-miniAID control did not develop auxin resistance after 19 days of 5-Ph-IAA treatment, suggesting the resistance in CTCF arose from a survival dependency on the protein itself. We are currently performing more studies to uncover the mechanism which leads to auxin resistance. Still, we feel these observations support that CTCF is essential for survival, and that when cells are stressed with CTCF depletion, they find a way to compensate by maintaining CTCF occupation at persistent CBSs and eventually evading degradation. As the studies into the mechanism behind CTCF’s auxin resistance are ongoing, we would like to present this evidence to reviewers but reserve this data for publication until this story is complete, if that is acceptable. In this manuscript, we will highlight the reduced, persistent binding of CTCF at a subset of CBSs may be sufficient to support survival.*

*Response Figure R1. a) Immunoblot analysis of CTCFAID2 cells (lanes 1-7 after the marker) and CTCFAID2/WT cells (lanes 8-11 after the marker) treated with 1 M 5-Ph-IAA over a 21-day time course. CTCF-AID corresponds to CTCFAIDmClover3 and HA-WT-CTCF corresponds to exogenously induced HA-tagged WT-CTCF. b) Immunoblot analysis of auxin sensitive (parental CTCFAID) or auxin resistant (resistant CTCFAID) CTCFAID2 cells challenged with increasing amounts of 5-Ph-IAA. Auxin resistant cells were generated after continuous treatment of 1 M 5-Ph-IAA for 19 days. c) Immunoblot analysis of CTCFAID2 cells with auxin sensitive (lanes 1-2 after the marker) or auxin resistant (lanes 3-7 after the marker) CTCFAID2 cells. Auxin was washed out for 48 hours (lane 4; red arrow), and cells were challenged with 1 M 5-Ph-IAA for 6 hours (lane 5). In lane 6, auxin was washed out for 6 days, and cells were rechallenged with 1 M 5-Ph-IAA for 24 hours (lane7). The internal EGFP-AID control was blotted against miniAID antibody and remained sensitive to all auxin treatment conditions.*

*According to reviewer’s suggestion, we also did a literature search and found that Valton et al. (doi: https://doi.org/10.1101/2021.07.29.454218) concluded that CTCF-C term-AID degradation following auxin treatment was inefficient in their cell line, which they inferred from the persistence of looping interactions following treatment (data was not shown in their preprint). They suggested that CTCF isoforms that did not contain the AID C terminal tag were expressed but did not provide evidence. In our experiments, auxin depletion of C-terminal tagged CTCF in SEM cells does not have the same limitation. CTCF HiChIP data showed a global reduction in CTCF looping (Fig. 1m), and ChIP seq showed a marked decrease in CTCF binding across all CTCF binding sites (Fig. 1l). However, some CTCF does remain in the cells at a low level that is not easily detectable by immunoblot. As the reviewer suggested, to exclude the possibility that other isoforms of CTCF could be present or arise upon 5-Ph-IAA challenge, we ran Kallisto analysis [9] on total stranded RNA-seq of CTCFAID2 samples with and without 5-Ph-IAA treatment for 1, 4 and 7 days to estimate the genome-wide isoform expression (these samples correspond to those described in the methods section titled ‘RNA-seq’). The abundance of each CTCF isoform was calculated as the transcripts per million mapped reads (TPM) of the isoform divided by the sum of the TPM values of all CTCF isoforms in the same sample. The mean value between replicates was calculated. The top three abundant isoforms of CTCF are shown below in Response Figure R2a-b. Isoform X-1, which corresponds to the fully transcribed CTCF, was the most dominant isoform and represented in all samples. Isoforms X-2 and X-3 were minimally detectable, and no isoforms changed expression patterns following auxin treatment. Moreover, suppose the reviewer also refers to the hypothesis that “other unknown proteins” may compensate for the loss of CTCF. In that case, it is an open question that would require a genetic screening and extensive study in the future.*

*Response Figure R2. No isoform shift was detected after AID2 depletion of CTCF. a) Bar plot showing the proportion of the most abundant CTCF isoforms in CTCFAID2 cells with and without 5-Ph-IAA treatment as estimated by RNA-Seq by Expectation-Maximization (RSEM). b) Plot showing the structure of the isoforms identified by RSEM.*

c) unclear to what extent proliferation / gene expression changes are contributed to by Tir1 as opposed to degrading CTCF

Authors did not include Tir1-expression CTCF-untagged cells in any of their experiments. Therefore it is very difficult to estimate to what extent AID1/2 tagging in itself (before inducing depletion) may disrupt CTCF expression or functions. Authors also do not provide data regarding auxin/ 5-Ph-IAA treatment in TIr1 expressing cells. Therefore, it is not possible to conclude that proliferation or cell cycle defects are solely due to CTCF depletion and not ectopic degradation of non-AID targets upon induction.

*Response: Based on the cell growth assays presented above, proliferation defects observed using AID1 conditions of high auxin and doxycycline induction of OsTIR1 WT showed that high auxin treatment alone reduced cellular proliferation in SEM cells, even without CTCF degradation (Additional File 1: Fig. S1e). Induction of OsTIR1 WT in CTCFAID1 cells also led to leaky degradation of CTCF (Additional File 1: Fig. S1d). In contrast, SEM cells with OsTIR1(F74G) expression and untagged CTCF (SEMOsTIR1(F74G)) proliferated comparably to SEMWT, even with 5-Ph-IAA treatment (Additional File 1: Fig. S1g). In addition, immunoblot analysis showed that CTCF protein expression was not destabilized following miniAID tagging, and CTCF degradation in CTCFAID2 cells was only observed after 5-Ph-IAA treatment (Fig. 1c). The data supports that the AID2 system, which uses hundreds of folds less auxin and integrated OsTIR1(F74G), is superior to achieve miniAID tagged protein degradation without the limitations of drug toxicity or leaky degradation.*

*To determine to what extent AID1 treatment conditions contributed to transcriptional dysregulation, differential gene expression was analyzed between SEMWT and CTCFAID1 cells following AID1 treatment, and CTCFAID2 cells following AID2 treatment. Total RNA was collected after 24 hours* *treatment and total-stranded RNA-seq was performed. When comparing SEMWT and CTCFAID1 cells following AID1 treatment, 196 genes (82 up, 114 down) were differentially expressed, including genes associated with amino acid metabolism, apoptosis, and ER stress (see below: Additional File 1: Fig. S5a-b). Pathways enriched for DE following treatment included the krige amino acid deprivation, hallmark TNFA signaling, and hallmark unfolded protein response (see below: Additional File 1: Fig. S5c). Spearman’s correlation between AID1 treated SEMWT and CTCFAID1 cells showed some correlation in DE genes, suggesting dysregulation of these genes resulted from auxin treatment alone (see below: Additional File 1: Fig. S5d). When SEMWT cells treated with AID1 conditions were compared to CTCFAID2 cells treated with AID2 conditions, no significant correlation was observed, highlighting that CTCFAID2 cells treated with low auxin did not develop transcriptional dysregulation attributed to high auxin toxicity (see below: Additional File 1: Fig. S5e). CTCFAID1 and CTCFAID2 cells treated with either high auxin and doxycycline (AID1) or 5-Ph-IAA (AID2) demonstrated a correlation of DE genes that corresponded to those genes dysregulated by CTCF loss (See below: Additional File 1: Fig. S5f). This information has been added to the manuscript and included in Additional File 1: Fig. S5.*

*Additional File 1: Fig. S5. a) MA-plot of SEMWT cells following AID1 treatment (500 M IAA; 1 g/ml doxycycline) for 24 hours. The log2 average mean expression is shown on the x-axis and Log2(FC) is shown on the y-axis. The most significant differentially expressed genes (|log2(FC)|>1, FDR < 0.05) are shown as red dots. b) Differential expression of the ASNS, DDIT3 and TRIB3 genes by RNA-seq (FKPM) in response to 24 hours AID1 treatment in SEMWT cells. c) GSEA plot of the top three enriched pathways following 24 hours AID1 treatment in SEMWT cells. d) Scatter plot showing the correlation between the 24h transcriptional changes of treated SEMWT (WT) and AID1-treated CTCFAID1. The x-axis shows the log2(FC) of SEMWT cells after IAA treatment for 24 hours. The y-axis represents the log2(FC) of CTCFAID1 cells after IAA treatment for 24 hours. e) Scatter plot showing the low correlation between 24h transcriptional change of treated AID1 SEMWT (WT) and AID2-treated (1 M 5-Ph-IAA) CTCFAID2 cells. The x-axis shows the log2(FC) of SEMWT cells after IAA treatment for 24 hours. The y-axis represents the log2(FC) of CTCFAID2 cells after 5-Ph-IAA treatment for 24 hours. f) Scatterplots showing the correlation between 24h transcriptional change of AID1-treated CTCFAID1 cells and AID2-treated CTCFAID2 cells. The y-axis represents the log2(FC) of CTCFAID1 cells after IAA treatment for 24 hours. The y-axis represents the log2(FC) of CTCFAID2 cells after 5-Ph-IAA treatment for 24 hours. d-f) Both Spearman and R2 correlation values are shown.*

2. Poor reporting of gene expression data

- In figure 2 how does authors quantify total RNA changes? It is not possible to understand Figure 2C with the information provided. please explain in the legend what "steady-state" / "total" means. mRNA-seq separately in AID1 and AID2? After depleting for how long?

*Response: We apologize for the confusion. By “total RNA changes”, we were referring to the total reads (the superset of reads including t>c converted and unconverted reads), also described as steady-state in the original SLAM-seq paper [10]. Treatment was carried out for various time points (example T0= no treatment, T2=2-hour treatment, T4= 4 hours treatment, etc.) using AID1 (500 M IAA and 1g/ml doxycycline) and AID2 (10 M 5-Ph-IAA) treatment conditions separately. We have made changes to the main text and figure legend to better represent this point.*

- Authors do not report the fold change of dysregulated genes

*Response: Our threshold for determining DEGs has been updated to p-value < 0.05 and |log2FC| > 1. Thus, all genes presented in Fig. 2c are |log2FC| > 1 in each comparison.*

- In figure 5a what does "RNA-seq"mean? SLAM-seq nascent transcripts? Other data not described in the methods section?

*Response: RNA-seq here measures steady-state transcription levels detected by total RNA-seq. These methods were described in the methods section and were titled “RNA-seq” and “RNA-seq data analysis.” We also clarified this point in the text.*

- It is very unusual to detect more genes dysregulated with TOTAL RNA vs nascent (fig 2) - without more information on where the numbers behind "TOTAL" comes from it is impossible to provide further advice.

*Response: We thank the reviewer for this comment. Our original normalization scheme did not take into account of global changes in the t>c converted read analyses. To correct this, we optimized our normalization scheme by using the normalization factors calculated from the steady state reads and applying this to the t>c converted reads. As mentioned above, “total RNA changes”, were referring to the total reads, also called steady state (the superset of reads including t>c converted and unconverted reads). The number of called steady-state DEGs versus t>c converted read DEGs is very sensitive to the thresholds used; originally, we used a simple threshold of FDR<0.05, which causes a large number of DEGs called in steady-state analyses. Taking the reviewer’s comment into consideration along with the optimized normalization scheme, we now call DEGs using a cutoff as p < 0.05 and Ilog2FCI > 1. Changes to these results can be seen in Fig. 2b, Fig. 2c, Fig. 2d, and Additional File 1: Fig. S4b. For the interest of reproducibility, commands and code have been made available (see the code link at the end).*

3. Biological insight into CTCF biology largely redundant with previous studies

Previous work already demonstrated that the entire N and C termini of CTCF appear dispensable for cell viability in culture (e.g. Nishana et al. Genome Biology 2019, Nora et al. 2020 Nature Comms), albeit serving biological functions (e.g. RNA-binding, cohesin binding, trans-activation etc…). Same for ZF1 and ZF10 (Saldana-Meyer et al Mol cell 2019). Saldana-Meyer et al Mol cell 2019 and Nakahashi et al Cell Reports 2014 also already showed that CTCF binding is disrupted in these mutants. Hansen et al. 2019 Mol Cell and Nora et al. 2020 Nature Comms already showed that deleting the RBRi also disrupts CTCF binding.

Nakahashi et al Cell Reports 2014 already reported that disruption of ZF1 or ZF10 disrupts CTCF binding depending on the underlying DNA motif. The analysis presented here in figure 6 does move beyond, by reporting specific nucleotide dependency in different classes of sites. However, experiment presented do not address whether the functional importance of ZF1/ZF10 detected in the genetic screen is actually mediated by DNA binding, as opposed to other processes (such as the ability of these domains to mediate RNA binding - Saldana-Meyer et al Mol Cell 2019).

*Response: We would like to thank the reviewer for this comment. We are fully aware of the similar studies from other groups and agree that CTCF biology has been extensively studied. However, we are confident to address the novelty of our findings based on the following aspects.*

*First, many previous investigations were performed in mice [11-13] but not human cells. Even within the mouse species, conservation of CTCF binding sites was found to be only 27% [14].*

*Second, Nakahashi et al. [15] performed intricate studies to extend the understanding of the DNA binding properties of CTCF’s zinc finger domains. However, their study was conducted using exogenously expressed CTCF mutants in WT mouse B cells without adjusting endogenous CTCF expression, which differs from our systems. Here, our human cell lines were induced by doxycycline to express CTCF WT or mutants at levels comparable to endogenous CTCF but with degradation of endogenous CTCF from the background. This is the first “swap system” study of CTCF that excludes the potential cross-communication between CTCF mutants and the endogenous WT form.*

*Moreover, looking into the technical details, we found more advanced findings from our system. Nakahashi et al. reported three binding regions corresponding to the ZF domain: the CTCF upstream (U), core (C) and downstream (D) motifs. While our observations regarding the (U) motif complement their work and others [15-19], the (D) motif reported does not overlap the motif we identified associated with ZF1 binding. For example, their analysis found the (D) motif to be located 6-8 bp from the 20 bp core motif, but the (D)-like motif we identified overlapped the 20 bp core motif. The differing locations of (D)-like motifs could be due to discrepancies in binding between human and mice, or it could be due to the nature in which CTCF binds LTR since Nakahashi et al. could not associate the (D) motif to any ZF and our analysis found it highly conserved. Interestingly, our finding about the ZF1 signature was validated by another independent study that was published through fast track [20]. However, since this finding was published after our submission, the editorial board has agreed this won’t compromise our novelty.*

*Hansen et al. and Saldana-Meyer et al. reported the RNA binding properties of the C-terminus of CTCF [13, 21]. However, in our study, deletion of the C terminal RNA binding region (RBR) did not cause many changes to CTCF DNA binding (Fig. 4c and 4f). Additionally, the CRISPR screen did not identify the RBR region as essential for survival. Fig. 5b also showed no correlation between genes DE following CTCF loss and genes with CTCFAID2/dRBR disrupted binding. In another study, the ZF1 and ZF10 have also been reported to be associated with RNA binding affinity [12]. To further examine how RNA may affect CTCF binding affinity in our model, we performed ChIP-seq using CTCFAID2/WT cells treated with either Triptolide in culture for 4 hours to halt RNA Pol II transcription or rChIP [22] using RNase A treatment (Response Figure R3a). Under both treatment conditions, very few differential binding peaks were found when compared to untreated samples (Response Figure R3b-c). We concluded RNA does not appear to be as essential for CTCF binding to DNA in our cell model, and RNA dependence for binding could be a tissue-specific effect as the previous functions were identified in mES cells. A more thorough study into the RNA-binding capability of CTCF is warranted in the future. If possible, we want to provide these data only to the reviewer but not include them in the manuscript.*

*Response Figure R3. a) Schematic diagram showing how RNA depletion by either Triptolide to inhibit RNA Pol II or RNase A would affect chromatin interactions dependent or independent of RNA. DNA binding events dependent on RNA would not enrich in ChIP-seq samples, whereas DNA binding events independent of RNA would enrich. b-c) Smoothed scatter plot showing the correlation between the log2 average CTCF ChIP-seq enrichment after RNase-A or Triptolide RNA depletion and the log2 average CTCF ChIP-seq enrichment in untreated samples.*

*The novelty of our study was the ability to link the dependence of ZF1 and ZF10 binding to function. By comparing steady-state RNA-seq data from CTCFAID2 cells with and without 5-Ph-IAA treatment to differential binding patterns observed from ChIP-seq in CTCFAID2/dZF1 and CTCFAID2/dZF10, we were able to identify genes that correlated a dependency of CTCF binding to ZF1 or ZF10 with transcription regulation (Fig. 5). Additionally, gene sets regulated by either ZF1 or ZF10 were mutually exclusive (Additional File 1: Fig. S7e). This is the first study to demonstrate peripheral ZF binding specifically regulates the transcription of a subset of genes, providing in vivo evidence to link function to the “CTCF code” of multivalent binding [15].*

*Additional File 1: Fig. F7e. Venn-diagram illustrating the DE/DB pair gene numbers identified in CTCFAID2/dZF1, CTCFAID2/dZF10, CTCFAID2/dRBR and CTCFAID2/WT.*

*In summary, our dRBR mutant swap, Triptolide and RNase A treatment experiments collaboratively suggest RNA-binding affinity do not seem to impact on CTCF’s DNA-binding affinity. However, whether there are other RNA-binding domains of CTCF, or RNA-binding affinity plays other functional roles in CTCF biology, still request further extensive investigation.*

Additional technical limitations of the study

- ChIP-seq experiments did not have spike-in controls. Exact quantification of binding strength is therefore not possible and conclusions remain qualitative when comparing the various mutants. While this is OK to state that CTCF binding is reduced after depletion it prevents stating how reduced it is and the effect in the various mutants is likely under/over estimated.

*Response: Thank you for raising this point. Our understanding is that the spike-in control is essential for scenarios when the protein levels between samples are dramatically different [23, 24]. For this reason, we did include a spike-in control for CTCFAID2 CTCF ChIP-seq with and without 5-Ph-IAA treatment (Fig. 1l). However, for HA-ChIP seq (Fig. 4d-f), we did not include a spike-in control because we didn’t observe an obvious protein level difference between samples (Fig. 4b). Additionally, others, such as Nakahashi et al. [15], observed reasonable results without added spike-in. Therefore, it was excluded for HA-ChIP seq to avoid introducing unnecessary variations. We also applied the “ChIPseqSpikeInFree” [25] method to 1) confirm there was no genome-wide change of binding; 2) reanalyze using the scaling-factor detected by ChIPseqSpikeInFree. In Response Figure R4a below, we plotted the cumulative distribution of the different CTCF mutants' ChIP-seq signal as estimated by the ChIPseqSpineInFree method. No difference in distribution between WT and the AID2 systems applied to CTCF, or the other mutants was observed. Additionally, the cumulative plot doesn’t show a hallmark of very severe depletion that needs spike-in (see next page: Response Figure R4b), which supports our decision not to use spike-in for this setting. While we mention that spike-in is added to the chromatin in the ChIP-seq methods, we will make the text clear as to which samples included the spike-in control.*

*Response Figure R4. a) Plot showing the proportion of reads as a function of the count per million for each 1-kb window (CPMW). No hallmark of severe depletion was observed after AID2 treatment in WT CTCF or CTCF mutants. b) Cartoon from the original ChIPseqSpikeInFree method [25] illustrates the typical cumulative distribution after total depletion. In this example, H3.3 K27M mutation globally affects H3K27me3 binding occupancy on chromatin.*

- Figure 1m. Doing Hi-ChIP for CTCF after CTCF degradation is uninformative. Since the protein is degraded it is of course expected that the CTCF HiChIP signal will be dramatically reduced. This approach therefore does not allow concluding anything about chromatin looping. I do not think this experiment is meaningful in any way. Hi-C of Hi-ChIP for a factor that is demonstrated to remain stable upon CTCF depletion could be used to address changes in genome folding but certainly not CTCF Hi-ChIP.

*Response: We would like to thank the reviewer for giving us the opportunity to explain our rationale for performing Hi-ChIP following CTCF degradation. In our previous work using AID1 [7], we observed genome-wide loop loss upon CTCF depletion using Hi-C. However, we realized that Hi-C doesn’t have enough resolution to pinpoint the precise loop anchors because of technique limitations, and it is also not possible to distinguish the CTCF-dependent loops versus loops that likely rely on other looping factors. Thus, we sought to perform HiChIP to help improve our and the communities’ ability to answer such questions in the future. In this manuscript, we used CTCF HiChIP to validate that the AID2 system worked not only to degrade CTCF protein levels, but also to disrupt known functions, including looping. Thus, CTCF HiChIP with 5-Ph-IAA treatment is an important positive control to show the signal reduction following 5-Ph-IAA treatment as the reviewer expected.*

I am sorry I cannot be more positive. I encourage authors to consider solidifying the characterization of the AID2 system with controls detailed above, clarify the presentation and exploration of the transcription data and, if possible, use their data to design additional experiments/analyses that are less confirmatory to yield more novel insight on CTCF biology.

*Response: We appreciate the insightful critiques this expert reviewer provided. We believe the additional experimental results and revision have significantly strengthened the manuscript's scientific content.*

Reviewer #2:

In this manuscript, Hyle et al. introduced an improved auxin-inducible degron (AID) technology, AID2, to interrogate CTCF protein level and its genome-wide binding. They found that the AID2 system was better than the previous AID1 system for rapid degrading CTCF with reduced cellular toxicity. They used CRISPR screen combined with an induced exdogenous CTCF mutant model, to study the function of zinc-finger (ZF) domains ZF1 and ZF10 in CTCF DNA binding and transcriptional regulation in the B-ALL SEM cell line system. The authors showed convincing evidence that AID2 can be a useful assay to study CTCF protein function, and provide insights into the functional relevance of ZF domains to CTCF molecular and regulatory functions, as demonstrating a broad interests in the field. The manuscript is overall well written, with data presented clearly. I have some specific comments as follows:

Major points:

1. The authors stated that 27,727 out of the 46,556 CTCF peaks "were completely lost following 24 hours treatment" (lines 128-129). This is a very important result that should be better presented in the figure. Fig 1l shows 46,620 peaks. Is it true that only 64 peaks in the 46,620 "combined peaks" are not "reproducible peaks"? How are "combined peaks" defined and what is the difference?

*Response: For each sample, we identified “high confidence” (FDR<0.05) and “low confidence” peaks (FDR<0.5). We considered a peak reproducible in each condition if it was called a “high confidence” peak in one or more replicates and overlapped “low confidence” peaks in the other replicates. The figure below illustrates our definition (Response Figure R5). We then merged the reproducible peaks from both conditions to create a reference peak set (i.e., “combined peaks”) for counting reads and performed differential binding analysis. In summary, we called 46,620 reproducible peaks from -5-Ph-IAA and + 5-Ph-IAA combined, 46,556 reproducible CTCF peaks in -5-Ph-IAA only, and 27,727 reproducible CTCF peaks in +5-Ph-IAA only. It is worth to note that although 64 peaks were called reproducible only in +5-Ph-IAA only, most of them could still be called in one or two replicates from -5-Ph-IAA samples. Therefore, we are confident there are no gained CTCF peaks in +5-Ph-IAA.*

*Response Figure R5. Schematic diagram showing the reproducible peak definition strategy. For CTCF TF peak calling, we focus on sharp peaks. As shown for 3 replicates in each experiment, “High-confidence peaks” are shown as large peaks with a darker blue color and “Low-confidence peaks” as small peaks with a light blue color. According to our experience, the reproducible peaks strategy (shown with the green bar) gives less false-positive calls compared to merely just doing the peak union between replicates.*

Also, it would be better if the 27,727 lost peaks can be clearly labeled in Fig. 1l.

How are the peaks ranked in Fig 1l? A more detailed description should be included. More importantly, what are the differences between the 27,727 lost peaks and the remaining insensitive peaks? Do they tend to be stronger peaks or weaker peaks in untreated cells? Do they show different distribution in the genome? i.e., are they more enriched in TAD boundaries, promoters, enhancers, or evenly distributed across all peaks?

*Response: We thank the reviewer for the suggestion. We double-checked the results, and it turns out there was a typo in the original manuscript. In total, we detected 27,727 reproducible CTCF peaks in +5-Ph-IAA samples, thus, 19,291 reproducible peaks were lost. As suggested, we re-plotted the heatmap to separate lost peaks and significantly reduced peaks (Additional File 1: Fig. S2a). All our heatmaps were ranked by the sum of intensity as a default of deeptools [26]. Interestingly, when compared within the untreated cells, we observed that the totally lost peaks had a weaker CTCF binding affinity compared to the peaks that were significantly reduced (Additional File 1: Fig. S2b). In terms of genomic distribution, we didn’t see a significant difference between the lost and significantly reduced peaks (Additional File 1: Fig. S2c). However, when we focused on the lost or significantly reduced CTCF peaks with a stringent cutoff (FDR<0.05, |log2(FC)|>1), we saw that the most CTCF peaks tend to be located at promoters. In contrast, the depleted peaks are located in non-promoter regions (Additional File 1: Fig. S2d). Additionally, while the most significantly reduced peaks (FDR < 0.05, |log2(FC)|>1) tend to be slightly closer to a TAD boundary, they didn’t show any statistically significant difference to the total lost peaks (kolmogorov-smirnov test) (Additional File 1: Fig. S2e). We will provide this more thorough analysis of the ChIP-seq data in the updated Additional File 1: Fig. S2, along with a corresponding explanation in the manuscript.*

*Additional File 1: Fig. S2. Characteristics of CTCF lost and resistant peaks after AID2 depletion. a) CTCF binding enrichment heatmap centered at CTCF peaks showing the binding intensity of CTCF peaks before and after 5-Ph-IAA treatment. Two main groups are identified: i) CTCF peaks that decreased in intensity but were not completely lost, and ii) CTCF peaks that were completely lost after 5-Ph-IAA treatment. b) Meta plot showing the mean CTCF peak intensity in untreated cells of the totally lost and reduced peaks. c) Stacked Bar-plot showing the genomic distribution of CTCF peaks from panel a. d) Genomic distribution of CTCF peaks that are more significantly depleted or resistant (FDR<0.05, FC>2). e) Boxplot plot showing the genomic distance of lost and significantly reduced CTCF peaks to nearby domain boundaries identified by HiChIP.*

2. In the HiChIP analysis shown in Fig. 1m, what do the APA numbers (2.684 & 1.338) mean? The detailed procedure of the aggregate peak analysis needs to be included in the manuscript. How many loop regions were included in the analysis for each of the two plots? How were normalizations and quantitative comparisons were done? Also, for the lost and retained loops, are they primarily enhancer-promoter (E-P) loops, E-E loops, or P-P loops? How many loop anchors colocalize with CTCF binding sites? Are the lost chromatin loops colocalized and correlated with lost CTCF binding sites? Also, it is unclear how the analysis was done in Extended Fig. S1c-d, especially the Hi-C data. Why the Hi-C and HiChIP (untreated) have so few overlapped loops? These analyses are essential to demonstrate the effect of CTCF degradation to chromatin conformation changes.

*Response: To address this concern, we now have provided more details for clarification. APA scores refer to the ratio of the mean central pixels to the mean of pixels in the lower left corner (a.k.a P2LL) in Aggregate Peak Analysis [6]. We used APA function from Juicer pipeline (v1.5, [61]) for the analysis at 5kb resolution then plotted using normalized signal (“normedAPA” version from Juicer APA output, https://github.com/aidenlab/juicer/wiki/APA). We have amended the methods to make them more straightforward. For APA analysis in Fig. 1m, we included 4,731 (4,507+224) HiChIP-only loops as -5-Ph-IAA and 329 +5-Ph-IAA loops, both corresponding to Additional File 1: Fig. S3b. We previously excluded loops called in HiC to avoid the effect from lower resolution of anchors. To avoid confusion for readers, we now replace Fig. 1m with APA analysis of CTCF HiChIP signals for -5-Ph-IAA and +5-Ph-IAA loops for all 7,220 loops called in CTCF HiChIP of -5-Ph-IAA. In this revised figure, loop numbers have changed since now Hi-C loops information were not considered except for the Venn Diagram in Additional File 1 Fig. S3c. The numbers in Venn Diagram were re-calculated after merging loops, which would show a difference given one loop in Hi-C could overlap 2 loops in CTCF HiChIP due to resolution difference.*

*To answer the questions regarding how loop anchors colocalize (6,852 lost, 368 retained and 355 new loops), we downloaded and processed H3K27ac data for SEM from GEO(GSM1934089). Based on the common nomenclature, the peaks called for H3K27ac that were not associated with a TSS could be considered enhancer peaks. Using a 2kb window, we counted how many loops could be grouped based on the overlapping of anchors with TSS or H3K27ac (anchors overlapping both TSS and an H3K27ac peak were assigned to Promoter; all anchors without TSS and H3K27ac were assigned to CTCF). Interestingly, lost loops have a similar percentage for P-P(p=0.066)/E-E(p=0.045) but significantly less P-E loops (p=0.001245, Odds Ratio=1.636, Fisher’s Exact Test) (Additional File 1: Fig. S3c). We also want to emphasize that we should be careful to interpret the “new” loops because their APA signal was very low, and the percentage of both anchors overlapping CTCF peaks was much lower (~30%) compared to others (>60%).*

*Additional File 1: Fig. S3c. Stacked Bar-plot showing the loops based on anchors distribution for retained (368), lost (6852) or new (355) loops. Each loop anchor was assigned to one out of three groups, with the following annotation, as Promoter (TSS +/- 2kb), Enhancer (SEM H3K27ac peak summit from GSM1934089) or CTCF.*

*72.1% of lost loop anchors (out of 9,862) overlap our CTCF peaks while 72.4% of retained loop anchors overlap CTCF peaks, which demonstrates loop anchors colocalize with CTCF binding sites.*

*When we correlated CTCF binding status to loop anchor regions lost after treatment, we observed 65.4% of lost loop anchors overlapped CTCF retained peaks, while only 11.3% overlapped CTCF lost peaks. Although the small percentage of overlap seems unexpected, this could be explained by several reasons:*

*a) Because one loop will have two loop anchors, a lost loop doesn’t have to lose binding at both anchors. If we check loop numbers out of 6,852 lost loops, 6,431 (93.9%) loops have at least one anchor overlapping a retained CTCF peak. About 1,383 (20.2%) loops have at least one anchor overlapping a lost CTCF peak. Thus, 14.1% of loops could overlap both retained and lost CTCF peaks. In contrast, 322 (87.5%) out of 368 retained loops overlapped retained CTCF peaks, while 61 (16.6%) overlapped lost CTCF peaks.*

*b) A lost loop may not require complete loss of CTCF binding. A significantly decreased peak could also explain loss of a loop. To check the correlation of binding intensity to loop anchor loss, we compiled the log2 (fold change) for CTCF peaks and separated them into two groups dependent on whether they overlapped lost loop anchors or overlapped retained loop anchors. We then compared their log2 (fold change) as shown below (Additional File 1: Fig. S3d). The results indicated that all CTCF binding was decreased, while the binding at CTCF peaks overlapping the anchors for lost loops were significantly more decreased when compared to the peaks overlapping retained loops.*

*Additional File 1: Fig. S3d. Boxplot with jitter for log2(fold change) of CTCF ChIP-seq signals (+5-Ph-IAA versus -5-Ph-IAA) at CTCF peaks either overlap with lost loop anchors or retained loop anchors. The t-test was used for statistical analysis.*

*Additional File 1: Fig. S3a (previously Extended Data Figure S1c) shows correlation analysis using HiCRep. We amended the figure legend to include details. For both Fig. S3a-b, please find a detailed explanation of the analysis in the Methods section titled “HiChIP/HiC data analysis”. We also compiled the analysis code at https://doi.org/10.6084/m9.figshare.21002533.*

*We were also surprised that only about 30% of loops were conserved between HiC and HiChIP. We have already conducted a comprehensive review of all aspects of analysis, from sequencing quality, and parameter choices to biological knowledge (such as CTCF head-to-head motif pattern). We are confident this result was not simply due to artifacts. To the best of our knowledge, we think this could be explained as:*

*a) HiC-only loops could be CTCF-independent and YY1-dependent loops [29].*

*b) HiChIP-only loops still show enrichment in APA analysis for HiC (Response Figure R6), indicating they could be real loops, but HiC did not call them due to the technical and sequencing depth difference. This could be because HiChIP has a higher signal-to-noise ratio, or because those loops were more dynamic, and the signal would be diffused without further enrichment using ChIP-seq.*

*Response Figure R6. Aggregate peak analysis (APA) plots from Hi-C and CTCF HiChIP of CTCFAID2 cells without 5-Ph-IAA treatment. The x/y-axis was centered at loop anchors spanning from -10 to +10 windows (window size 10kb), and the z-axis indicates normalized aggregated contact frequency. Column “Common” contains 2,479 common loops between Hi-C and CTCF HiChIP without 5-Ph-IAA. “HiChIP.Only” contains 4,731 loops that was only called in CTCF HiChIP without 5-Ph-IAA.*

3. Detailed PCA procedures that were used to generate Fig. 1k (ChIP-seq) and Fig. 2b (SLAM-seq) should be described in the Methods section. It is unclear how ChIP-seq peak signals were calculated and whether/how normalization was done. For Fig. 2b (AID2), it is unclear why PC1 (with only 16.45% variance explained) can indicate "gradual and constant shift in transcript variability over time" (line 153).

*Response: We updated the methods section of RNA-seq and SLAM-seq to explain how the normalization and PCA were calculated. For ChIP-seq, we counted the number of fragments in each peak for each sample. Then we used Trimmed Means of M values normalization (TMM). For samples with spike-in, we used the percentage of uniquely mapped spike-in reads to normalize the data.*

*For original line 153, PC1 generally correlates with the time of treatment in PCA plots. However, for AID1, this correlation was predominantly treatment dependent as indicated by the clustering of untreated samples (on the left) versus treated samples (clustered together on the right), indicating the genes contributing to PC1 were in response to +IAA/Dox, not decreasing levels of CTCF. However, the treatment samples did not cluster together in AID2, indicating those genes contributing to PC1 were more correlated with treatment time and subsequent CTCF degradation instead of a binary response to +5-Ph-IAA.*

4. In lines 171-173, the authors claim that "some of the transcription dysregulation observed in the MYC target pathways using the AID1 system may be attributed to off-target effects of combined high concentration auxin treatment and CTCF depletion." How was this conclusion made based on the pathway enrichment analysis? This statement seems unfounded and needs more evidence to support and more elaboration.

*Response: We agree this is an assumption based on our current data and is not definitively proven. We reached this assumption based on the following observations:*

*1. In Fig. 2d, we utilized SLAM-seq’s ability to detect nascent RNA to better identify the direct transcriptional targets of CTCF following degradation by AID1 or AID2. We compared the GSEA enrichment results between the AID1 and AID2 systems. We found MYC TARGETS (both V1 and V2) were strongly enriched as early as 2 hours post-treatment in AID1. However, this would be at a time point when CTCF levels were not greatly diminished under AID1 settings (Fig. 1e). In contrast, only MYC TARGETS V2 were strongly enriched starting at 4 hours post-treatment in AID2, which does correspond with efficient CTCF degradation and supports this pathway change was a direct response of CTCF loss.*

*2. Additionally, growth assays in SEMWT and CTCFAID1 cells indicated high auxin treatment was not well tolerated, whereas AID2 treatment caused no proliferation defect (Additional File 1: Fig. S1e-g). From this observation, we expect transcriptional defects would also be present following high auxin treatment.*

*3. RNA-seq analysis of SEMWT and CTCFAID1 cells showed a clear correlation of genes dysregulated following high auxin treatment (Additional File 1: Fig. S5d). However, MYC TARGETS V1 and V2 were not enriched in SEMWT treated cells, only CTCFAID1 treated cells.*

*4. In Fig. 2d, for AID1, there are a few pathways that were enriched in AID1 but not or weakly enriched in AID2, such as INFLAMMATORY RESPONSE, P53 PATHWAY, APOPTOSIS. If we looked into the SLAM-seq data for MYC-AID (AID1) in HCT116 or K562 from Muhar et al. [10], we could not find these pathways enriched indicating they were not MYC direct targets.*

*Since CTCF is concurrently reduced in combination with high auxin, it seems plausible the combination of high auxin and CTCF depletion could be responsible for the early enrichment of MYC target pathways. We can adjust the text to make it clear this is our assumption and not a definitive conclusion.*

5. The authors use DESeq2, and RNA-seq differential analysis tool, to identify differentially represented sgRNAs. However, MAGeCK (PMID: 25476604) is more appropriate for analyzing CRISPR screen data. Some justification is needed to support the authors' choice.

*Response: Thank you for the comment. We indeed used the MAGeCK algorithm as described in the methods and the Figure 3d legend. We apologize for the error in the manuscript, and we will correct this in the revised version.*

6. In lines 240-241, the authors state that "CTCF binding can regulate transcription either proximally to the transcription start site (TSS) or distally within the target's TAD boundaries." Additionally, in fact, CTCF can also regulate transcription by binding at distal enhancer regions within a TAD but distinct from TAD boundary regions (e.g., Schuijers et al 2018 PMID: 29641996, Fang et al 2020 PMID: 32933554). I would suggest that the authors revise the statement and cite these references.

*Response: Thank you for this suggestion. We have updated the statement and references.*

7. In lines 241-243, the authors state "The 50kb region upstream and downstream of the TSS and transcription end site (TES), respectively, were considered when assigning CTCF binding to DE genes." This contradicts to the description in the Methods section (lines 606-608), where they say "TSS+/-50kb". This should be clarified.

*Response: Thank you for bringing this point to our attention. We have corrected the manuscript to define [TSS-50kb, TES+50kb] in the method section.*

8. For the statistical analysis shown in Fig. 5b and Extended Fig. S4, p-values should be presented in addition to F-statistics to properly demonstrate the statistical significance. For the dRBR control (Fig. 5b right and Fig. S4c), F-statistic should also be presented even if the p-value does not meet the significance threshold. Also, R^2 is not an appropriate metric to use, as the data points were preselected as differential genes and peaks, not reflecting the whole sample.

*Response: Thank you for the suggestion. We agree with the reviewer that the R2 is not the appropriate measure, thus we removed it from the figure. The p-values here can also be misleading for the interpretation as they measure the significance that the fitted line slope is different from zero and not the size of the effect. Thus, in the scatter plots we report the number of significant DB/DE interactions detected. To reliably measure the effect, we calculated the significance in the log2(FC) of the differential binding of the associated peaks identified in Additional File 1: Fig. S7 a-c (the difference in the effect on the y-axis).*

*Additional File 1: Fig. S7. a-c) Scatter plot showing the integrative analysis results of RNA-seq and differential binding of HA-CTCF-ZF mutants. For each gene, we initially select all the peaks within [TSS-50kb, TES+50kb]. The RNA-seq and ChIP-seq fold-changes were converted to z-score, then multiplied together for each gene-peak pair to get a combined score. Pareto optimization was performed to determine most correlated peak-gene pairs. Gene cutoff: DB:|FC|>2 and FDR <0.05; DE: |FC|>2 and FDR < 0.05. DE/DB pairs that passed |FC| >2 FDR<0.05 were highlighted by orange color>. d) Boxplot showing the absolute differential binding log2(FC) of the peaks of each HA-CTCT-ZF mutant identified by the integrative analysis. P-values were calculated using the Wilcoxon rank-test.*

Minor points:

9. Lines 225 and 601: Citations of PMIDs need to be included in the references.

*Response: Thank you for this comment. We have now included the references.*

10. In addition to a more detailed description of computational analysis methods, it would be helpful to include the complete code and scripts in a code repository, to ensure reproducibility of the results.

*Response: Thank you for this comment. We would like to provide complete end-to-end code and scripts for all the analysis; however, it’s quite challenging given all the pipelines heavily depend on computational clusters environments. For example, for the Hi-C processing pipeline, Juicer itself needs the user to install based on their job submission software (LSF, AWS, etc.) and reference files created based on genome build and enzymes used. Thus, we are doing our best to ensure the reproducibility of results by compiling code repositories collected at:*

*(https://doi.org/10.6084/m9.figshare.c.6186670), the collection included ChIP-seq QC (https://doi.org/10.6084/m9.figshare.21002533), Integrative analysis ChIP-seq and RNA-seq (https://doi.org/10.6084/m9.figshare.21045889), Hi-C and HiChIP analysis (https://doi.org/10.6084/m9.figshare.21002533), SLAM-seq analysis (https://doi.org/10.6084/m9.figshare.21259278).*

*The example commands and customized were also included to improve the reproducibility of our data.*

*Reference*

*1. Sathyan, K.M., et al., An improved auxin-inducible degron system preserves native protein levels and enables rapid and specific protein depletion. Genes Dev, 2019. 33(19-20): p. 1441-1455.*

*2. Yesbolatova, A., et al., The auxin-inducible degron 2 technology provides sharp degradation control in yeast, mammalian cells, and mice. Nat Commun, 2020. 11(1): p. 5701.*

*3. Natsume, T., et al., Rapid Protein Depletion in Human Cells by Auxin-Inducible Degron Tagging with Short Homology Donors. Cell Rep, 2016. 15(1): p. 210-218.*

*4. Zhang, H., et al., Functional interrogation of HOXA9 regulome in MLLr leukemia via reporter-based CRISPR/Cas9 screen. Elife, 2020. 9.*

*5. Luan, J., et al., Distinct properties and functions of CTCF revealed by a rapidly inducible degron system. Cell Rep, 2021. 34(8): p. 108783.*

*6. Khoury, A., et al., Constitutively bound CTCF sites maintain 3D chromatin architecture and long-range epigenetically regulated domains. Nat Commun, 2020. 11(1): p. 54.*

*7. Hyle, J., et al., Acute depletion of CTCF directly affects MYC regulation through loss of enhancer-promoter looping. Nucleic Acids Res, 2019. 47(13): p. 6699-6713.*

*8. Nora, E.P., et al., Targeted Degradation of CTCF Decouples Local Insulation of Chromosome Domains from Genomic Compartmentalization. Cell, 2017. 169(5): p. 930-944.e22.*

*9. Bray, N.L., et al., Near-optimal probabilistic RNA-seq quantification. Nat Biotechnol, 2016. 34(5): p. 525-7.*

*10. Muhar, M., et al., SLAM-seq defines direct gene-regulatory functions of the BRD4-MYC axis. Science, 2018. 360(6390): p. 800-805.*

*11. Nishana, M., et al., Defining the relative and combined contribution of CTCF and CTCFL to genomic regulation. Genome Biol, 2020. 21(1): p. 108.*

*12. Saldaña-Meyer, R., et al., RNA Interactions Are Essential for CTCF-Mediated Genome Organization. Mol Cell, 2019. 76(3): p. 412-422.e5.*

*13. Hansen, A.S., et al., Distinct Classes of Chromatin Loops Revealed by Deletion of an RNA-Binding Region in CTCF. Mol Cell, 2019. 76(3): p. 395-411.e13.*

*14. Kentepozidou, E., et al., Clustered CTCF binding is an evolutionary mechanism to maintain topologically associating domains. Genome Biol, 2020. 21(1): p. 5.*

*15. Nakahashi, H., et al., A genome-wide map of CTCF multivalency redefines the CTCF code. Cell Rep, 2013. 3(5): p. 1678-1689.*

*16. Rhee, H.S. and B.F. Pugh, ChIP-exo method for identifying genomic location of DNA-binding proteins with near-single-nucleotide accuracy. Curr Protoc Mol Biol, 2012. Chapter 21: p. Unit 21 24.*

*17. Yin, M., et al., Molecular mechanism of directional CTCF recognition of a diverse range of genomic sites. Cell Res, 2017. 27(11): p. 1365-1377.*

*18. Boyle, A.P., et al., High-resolution genome-wide in vivo footprinting of diverse transcription factors in human cells. Genome Res, 2011. 21(3): p. 456-64.*

*19. Schmidt, D., et al., Waves of retrotransposon expansion remodel genome organization and CTCF binding in multiple mammalian lineages. Cell, 2012. 148(1-2): p. 335-48.*

*20. Lebeau, B., et al., Single base-pair resolution analysis of DNA binding motif with MoMotif reveals an oncogenic function of CTCF zinc-finger 1 mutation. Nucleic Acids Res, 2022.*

*21. Saldana-Meyer, R., et al., CTCF regulates the human p53 gene through direct interaction with its natural antisense transcript, Wrap53. Genes Dev, 2014. 28(7): p. 723-34.*

*22. Long, Y.C., et al., RNA is essential for PRC2 chromatin occupancy and function in human pluripotent stem cells. Nature Genetics, 2020. 52(9): p. 931-+.*

*23. Nakato, R. and K. Shirahige, Recent advances in ChIP-seq analysis: from quality management to whole-genome annotation. Brief Bioinform, 2017. 18(2): p. 279-290.*

*24. Meyer, C.A. and X.S. Liu, Identifying and mitigating bias in next-generation sequencing methods for chromatin biology. Nat Rev Genet, 2014. 15(11): p. 709-21.*

*25. Jin, H., et al., ChIPseqSpikeInFree: a ChIP-seq normalization approach to reveal global changes in histone modifications without spike-in. Bioinformatics, 2020. 36(4): p. 1270-1272.*

*26. Ramirez, F., et al., deepTools: a flexible platform for exploring deep-sequencing data. Nucleic acids research, 2014. 42(Web Server issue): p. W187-91.*

*27. Rao, S.S., et al., A 3D map of the human genome at kilobase resolution reveals principles of chromatin looping. Cell, 2014. 159(7): p. 1665-80.*

*28. Durand, N.C., et al., Juicer Provides a One-Click System for Analyzing Loop-Resolution Hi-C Experiments. Cell Syst, 2016. 3(1): p. 95-8.*

*29. Weintraub, A.S., et al., YY1 Is a Structural Regulator of Enhancer-Promoter Loops. Cell, 2017. 171(7): p. 1573-1588.e28.*

**Second round of review**

**Reviewer 2**

The authors have thoroughly addressed all my previous comments in the revision. The revised manuscript has been greatly improved. I do not have any more major concerns except the following minor comments:

1. The authors clearly explain in the response to our previous comment #1 that 27,727 reproducible CTCF peaks were detected in +5-Ph-IAA samples. However, this number apparently becomes 27,262 in the revised manuscript's main text (lines 157 & 162). Could the authors further clarify the difference and report the final accurate numbers?

2. In Line 388-389, it reads "Out of 63 of 64 sites for CTCF^{AID2/dZF10}…". I assume they mean "63 out of the 64 sites". Please clarify and fix the language.

3. In the figure legend of Fig. 5d &5f, it should be clarified whether the p-values equals (=),less than or equal to (≤), or less then (<) 0.001 for d and 0.01 for f. "p value 0.01" lacks rigor.

Reviewer 3

Hyle et al have performed CTCF depletion using the AID2 system in SEM cells which is much less leaky than their previous AID line. The most interesting part of the manuscript is the reconstitution experiment (which they call swap experiment) in which a mutant version of CTCF is expressed in the absence of the endogenous CTCF. The authors find that dZF1 binds to a specific G upstream of the core motif and dZF10 binds and previously defined upstream motif.

The authors have addressed most of the comments of the reviewer. However, I have a few comments. I agree with reviewer 1 that Hi-ChIP cannot serve as a control for looping changes after CTCF depletion, because the ChIP is on CTCF. If the effect on looping is really drastic, one should be able to see it with a regular Hi-C, even shallowly sequenced, the effect should show up in an APA.

Other points:

A recent paper that showed similar results (Soochit et al. NCB PMID:34326481) is not cited.

Figure 6 naming is confusing: dZF1 vs WT decreased peaks, perhaps say WT CTCF binding strong than dZF1 CTCF.

**Authors Response**

**Point-by-point responses to the reviewers’ comments:**

Reviewer #2: The authors have thoroughly addressed all my previous comments in the revision. The revised manuscript has been greatly improved. I do not have any more major concerns except the following minor comments:

*Response: We appreciate the positive comments and insightful critiques this expert reviewer provided, which helped to significantly strengthen the scientific content of the manuscript. Thank you.*

1. The authors clearly explain in the response to our previous comment #1 that 27,727 reproducible CTCF peaks were detected in +5-Ph-IAA samples. However, this number apparently becomes 27,262 in the revised manuscript's main text (lines 157 & 162). Could the authors further clarify the difference and report the final accurate numbers?

*Response: We apologize for the typo and confirm that the correct number is presented in the manuscript now. The number 27,727 was a typo, and the correct number of reproducible peaks is 27,262. We have 27,727 reproducible peaks in three replicates of samples treated with 5-Ph-IAA. The typo happens due to the fact that at first, we wanted to describe the reproducible peaks. Later, we decided to describe the reduce peaks to as suggested but we forget change the number accordingly because the number was very close to the correct one by accident.*

2. In Line 388-389, it reads "Out of 63 of 64 sites for CTCF^{AID2/dZF10}…". I assume they mean "63 out of the 64 sites". Please clarify and fix the language.

*Response: Thank you for the comment. We want to change it to “The 63 out of the 64 sites for CTCFAID/dZF10”.*

3. In the figure legend of Fig. 5d &5f, it should be clarified whether the p-values equals (=), less than or equal to (≤), or less then (<) 0.001 for d and 0.01 for f. "p value 0.01" lacks rigor.

*Response: We apologize for the confusion. We updated the figure legend as “p≤0.001 for d), p≤0.01 for f”.*

Reviewer #3: Hyle et al have performed CTCF depletion using the AID2 system in SEM cells which is much less leaky than their previous AID line. The most interesting part of the manuscript is the reconstitution experiment (which they call swap experiment) in which a mutant version of CTCF is expressed in the absence of the endogenous CTCF. The authors find that dZF1 binds to a specific G upstream of the core motif and dZF10 binds and previously defined upstream motif.

*Response: Thank you for your nice comments.*

The authors have addressed most of the comments of the reviewer. However, I have a few comments. I agree with reviewer 1 that Hi-ChIP cannot serve as a control for looping changes after CTCF depletion, because the ChIP is on CTCF. If the effect on looping is really drastic, one should be able to see it with a regular Hi-C, even shallowly sequenced, the effect should show up in an APA.

*Response: We agree that Hi-C from both control and CTCF depletion samples will better represent genome-wide looping changes. In our previous work using AID1 (Hyle et al. Nucleic Acids Res. 2019 Jul 26;47(13):6699-6713. doi: 10.1093/nar/gkz462), we observed genome-wide loop loss upon CTCF depletion using Hi-C. Indeed, the APA plot shows that they were also decreased at Hi-ChIP loops (Response Figure R1) upon CTCF degradation. I reasonably speculate that similar results will be expected from the AID2 system.*

*Response Figure R1: Aggregate peak analysis (APA) plot from Hi-C. The x/y-axis was centered at loop anchors spanning from -10 to +10 windows (window size 10kb), and the z-axis indicates normalized aggregated contact frequency. HiC data were collected and analyzed from our previous work using AID1 (Hyle et al. Nucleic Acids Res. 2019 Jul 26;47(13):6699-6713. doi: 10.1093/nar/gkz462).*

*Moreover, as we know, Hi-C doesn’t have enough resolution to pinpoint the precise loop anchors because of technical limitations such as that both CTCF-dependent and independent loops will be identified. The primary purpose of using Hi-ChIP here in our current study is to identify the high-confidence CTCF-dependent loops in comparison with isogenic negative controls (+5-Ph-IAA). We also noticed the Hi-ChIP could pull a notable amount of DNA, while with similar depth and quality of data, negative controls (+5-Ph-IAA) only called few loops, likely because the loop signal in Hi-ChIP in +5-Ph-IAA was not strong enough to overcome the noise to be called as loops. Together, Hi-ChIP in +5-Ph-IAA 1) serves as a positive control to assure the AID2.0 system works well; 2) helps to further assure the loops called from Hi-ChIP in -5-Ph-IAA were CTCF-dependent; 3) helps to reduce false positive loops calling.*

Other points:

A recent paper that showed similar results (Soochit et al. NCB PMID:34326481) is not cited.

*Response: Thank you. We have added this reference now (Ref 50).*

Figure 6 naming is confusing: dZF1 vs WT decreased peaks, perhaps say WT CTCF binding strong than dZF1 CTCF.

*Response: Thank you. We have revised the naming as “dZF1 vs WT binding affinity decreased peaks”.*
